# Treatment Dose Increase Versus Co‐Medication in Allergic Rhinitis: Systematic Review With Dose–Response Network Meta‐Analysis

**DOI:** 10.1111/all.70372

**Published:** 2026-05-01

**Authors:** Bernardo Sousa‐Pinto, Rafael José Vieira, Sara Gil‐Mata, Ana Margarida Pereira, Vítor Henrique Duarte, Antonio Bognanni, Manuel Marques‐Cruz, Paweł Jemioło, Mattia Giovannini, Danilo di Bona, Arnav Agarwal, Mark S. Dykewicz, Mohamed H. Shamji, Maria Jose Torres, Torsten Zuberbier, Ludger Klimek, Alexandro W. L. Chu, Holger J. Schünemann, Guido Schwarzer, João A. Fonseca, Jean Bousquet, the ARIA 2024–2025 systematic reviews group, Ana Teixeira‐Ferreira, André Ferreira, António Cardoso‐Fernandes, Despo Ierodiakonou, Diana Portela, Ewa Borowiack, Ewelina Sadowska, Henrique Ferreira‐Cardoso, Hugo Viegas, Izabel Pera Calvi, João Castro‐Teles, José Laerte Boechat, Juliana Pereira‐Macedo, Maria Inês Torres, Marta Soares Pereira, Miguel Campos‐Lopes, Nuno Lourenço‐Silva, Pau Riera‐Serra, Paula Perestrelo, Raquel Albuquerque‐Costa, Renato Ferreira‐da‐Silva, Rita A. Silva Ribeiro, Tuuli Thomander

**Affiliations:** ^1^ MEDCIDS—Department of Community Medicine, Information and Health Decision Sciences; Faculty of Medicine University of Porto Porto Portugal; ^2^ CINTESIS@RISE—Health Research Network, Faculty of Medicine University of Porto Porto Portugal; ^3^ Allergy Unit, Instituto and Hospital CUF Porto Portugal; ^4^ Unit of Anatomy, Department of Biomedicine, Faculty of Medicine University of Porto Porto Portugal; ^5^ Clinical Epidemiology and Research Center (CERC) Humanitas University and Humanitas Research Hospital Milan Italy; ^6^ Department of Health Research Methods, Evidence, and Impact & Department of Medicine, Evidence in Allergy Group McMaster University Hamilton Ontario Canada; ^7^ AGH (Akademia Gorniczo‐Hutnicza) University of Krakow Krakow Poland; ^8^ Chair of Epidemiology and Preventive Medicine, Department of Hygiene and Dietetics Faculty of Medicine Jagiellonian University Medical College Krakow Poland; ^9^ Allergy Unit, Meyer Children's Hospital IRCCS Florence Italy; ^10^ Department of Health Sciences University of Florence Florence Italy; ^11^ Department of Medical and Surgical Science University of Foggia Foggia Italy; ^12^ Division of General Internal Medicine, Department of Medicine University of Alberta Edmonton Alberta Canada; ^13^ Department of Health Research Methods, Evidence and Impact McMaster University Hamilton Ontario Canada; ^14^ Section of Allergy and Immunology Saint Louis University School of Medicine Saint Louis Missouri USA; ^15^ National Heart and Lung Institute (NHLI) Imperial College London UK; ^16^ NIHR Imperial Biomedical Research Centre London UK; ^17^ Allergy Unit, Regional University Hospital of Málaga Malaga University, ARADyAL Malaga Spain; ^18^ Institute of Allergology, Charité—Universitätsmedizin Berlin Corporate Member of Freie Universität Berlin and Humboldt‐Universität Zu Berlin Berlin Germany; ^19^ Fraunhofer Institute for Translational Medicine and Pharmacology ITMP, Immunology and Allergology Berlin Germany; ^20^ Department of Otolaryngology, Head & Neck Surgery Universitätsmedizin Mainz Mainz Germany; ^21^ Center for Rhinology and Allergology Wiesbaden Germany; ^22^ Institute of Medical Biometry and Statistics Faculty of Medicine and Medical Center ‐ University of Freiburg Freiburg Germany; ^23^ ARIA (Allergic Rhinitis and Its Impact on Asthma) Montpellier France

**Keywords:** allergic rhinitis, dose–response network meta‐analysis, intranasal corticosteroids, oral antihistamines, systematic review

## Abstract

**Background:**

To achieve adequate symptom control, patients with allergic rhinitis (AR) often need to increase their medication dose or add other treatments (co‐medication). We aimed to perform a systematic review to compare the efficacy and safety of AR medications for increased dose versus co‐medication.

**Methods:**

We searched four bibliographic databases and three trial databases for randomised controlled trials assessing the effect of intranasal and/or oral medications in patients of all ages with seasonal or perennial AR. We performed pairwise meta‐analysis based on direct evidence to compare (i) non‐standard versus standard treatment doses, and (ii) co‐medication strategies versus monotherapy using standard doses. Furthermore, we fitted dose–response network meta‐analysis (NMA) to obtain projected estimates for comparisons involving two times the standard dose of AR medications in monotherapy versus co‐medication with the standard dose of the same medications. We assessed the certainty of evidence using GRADE for NMA.

**Results:**

We included 262 studies. Co‐medication schemes involving oral antihistamines (OAH) + intranasal corticosteroids (INCS) resulted in higher improvements of nasal symptoms and quality of life than doubling the dose of OAH. However, doubling the dose of intranasal medications led to better results than having intranasal medications + OAH. Doubling the dose of INCS was associated with higher efficacy than adding intranasal antihistamines (INAH). No relevant safety differences were found between treatment strategies.

**Conclusions:**

Results favoured (i) doubling the dose of intranasal medications versus adding OAH, and (ii) adding INCS to OAH over doubling the dose of OAH. This study will inform the ARIA‐EAACI 2024–2025 guidelines.

## 1 Introduction

In patients with allergic rhinitis (AR), the mainstay pharmacological treatment includes, among others, intranasal corticosteroids (INCS), intranasal antihistamines (INAH), fixed combinations of INAH + INCS and oral antihistamines (OAH). Guidelines on the pharmacological treatment of AR have focused on comparing these different pharmacological classes among themselves or versus no treatment [1, 2]. However, in more severe forms of the disease, the use of a standard dose of these treatments in monotherapy may be insufficient to adequately control AR symptoms. Treatment escalation may therefore involve either dose up‐titration or the addition of another agent from the same or a different pharmacological class. These approaches have indeed been reported for other mast cell and histamine‐depending diseases, such as urticaria, in which medication updosing is recommended [3]. However, it is unclear which of these two strategies is more effective, as there is a lack of randomised controlled trials (RCTs) comparing medication dose increase versus co‐medication/add‐on medication strategies in AR. Importantly, observational studies using mHealth data from MASK‐air have shown that patients frequently resort to co‐medication strategies when feeling insufficiently well controlled [4, 5]. These studies have found that co‐medication is associated with lower treatment satisfaction than monotherapy [6]. However, medication doses are not registered in MASK‐air, precluding a comparison between dose increase versus co‐medication. Finally, systematic reviews and meta‐analyses have not explored this question [7, 8, 9, 10, 11, 12]. In fact, traditional meta‐analytical methods have important limitations in dealing with different doses of the same medication, either (i) lumping together studies assessing different doses of a same intervention or (ii) treating each pair study‐dose as a separate intervention, resulting in disconnected or sparse networks. These methodological shortcomings can be overcome by the use of dose–response network meta‐analyses (NMA), which can compare different interventions while adequately fitting dose–response relationships for their different doses [13].

In this systematic review, we aim to compare the efficacy and safety of AR medications in increased dose (“dose escalation”) versus co‐medication. We start by evaluating direct evidence from head‐to‐head trials comparing (i) standard versus other doses of AR medications, and (ii) co‐medication versus monotherapy in standard doses. Subsequently, we perform dose–response NMA to compare intranasal and oral medications in double dose versus co‐medication. This systematic review will inform the Allergic Rhinitis and its Impact on Asthma (ARIA)—European Academy of Allergy and Clinical Immunology (EAACI) 2024–2025 guidelines [14], particularly the question “Should co‐medication vs. medication updosing be used in patients with allergic rhinitis that is poorly controlled despite pharmacologic treatment?” [15].

## 2 Methods

We performed a systematic review of RCTs evaluating intranasal or oral medications in patients with AR. We started by performing pairwise meta‐analyses of studies directly comparing non‐standard versus standard treatment doses, and (ii) co‐medication strategies versus monotherapy using standard doses. Considering the absence of studies comparing co‐medication versus treatment dose increase, we additionally performed dose–response NMA. This systematic review follows the Preferred Reporting Items for Systematic Reviews and Meta‐Analyses (PRISMA) extension for NMA (PRISMA‐NMA; Box S1) [16]. Its protocol has been published [17] and has been registered in PROSPERO (CRD420251113186). A full description of the methodology is available in the supplement.

### 2.1 Eligibility Criteria

We included RCTs with a parallel design assessing patients of any age with seasonal or perennial AR and evaluating the effect of (i) intranasal medications (INCS, INAH, or fixed combinations of INAH + INCS), (ii) oral medications (OAH or leukotriene receptor antagonists [LTRA]), or (iii) combinations of any intranasal and/or oral medications for AR. We considered studies in which these interventions were compared with placebo as well as those in which active interventions were compared among themselves.

We included studies reporting results on at least one of the following patient‐reported outcome measures: Total Nasal Symptom Score (TNSS), Total Ocular Symptom Score (TOSS), or Rhinoconjunctivitis Quality of Life Questionnaire (RQLQ). Considering the Food and Drug Administration recommendations, we only included RCTs with a follow‐up period of at least 2 weeks if assessing patients with seasonal AR or at least 4 weeks if assessing patients with perennial AR [18]. We did not exclude studies based on publication language, date, or status.

### 2.2 Information Sources and Search Strategy

We searched four bibliographic databases (MEDLINE and Embase via Ovid, Web of Science, and the Cochrane Central Register of Controlled Trials) and three databases of clinical trials (clinicaltrials.gov, the GSK clinical study dataset, and the AstraZeneca Clinical Trials Website). We considered the primary studies included in our previous systematic reviews that met the eligibility criteria [7, 8, 9, 10, 11, 12]. In addition, we updated the search in July 2025. The search strategy is available in Table S1.

### 2.3 Study Selection and Data Collection

For each record, study selection and data extraction were independently performed by two researchers. Disagreements between reviewers were resolved by consensus or by a third reviewer.

### 2.4 Risk of Bias and Certainty of Evidence Assessment

Two researchers independently evaluated the risk of bias of each included primary study (at an outcome level) using the Cochrane risk‐of‐bias tool [19]. Disagreements were resolved by consensus or by a third reviewer.

We evaluated the certainty of the evidence at an outcome level based on the GRADE approach for NMA [20, 21] and following the most recent GRADE guidance [22]. The adopted decision thresholds are displayed in Table S2. Considering the specificities of dose–response NMA, we adopted some special considerations when evaluating the certainty of evidence. In particular, (i) we assumed that a “comparison” is defined not only as a pair of treatments but rather as a pair of treatments and the respective doses, and (ii) we considered the doses of interventions evaluated in included primary studies when rating indirectness and intransitivity.

### 2.5 Quantitative Synthesis of the Evidence

Assessed efficacy outcomes (TNSS, TOSS and RQLQ) are continuous, having been described using mean (±standard‐deviation) baseline and change‐from‐baseline values. Assessed safety outcomes (development of at least one adverse event [AE], serious AE and withdrawals due to AE) are dichotomous and were described using absolute and relative frequencies. Both for pairwise and network approaches, we performed meta‐analysis of (i) mean differences in change‐from‐baseline values for efficacy outcomes, and (ii) risk ratios for safety outcomes. We did not perform meta‐analysis on the frequency of serious AE due to the scarce number of events.

Analyses were performed in R using the packages netdose and meta [23].

### 2.6 Pairwise Meta‐Analysis

As first steps in our analysis, we performed pairwise random‐effects meta‐analysis comparing (i) standard versus other doses of AR medications, and (ii) co‐medication versus monotherapy in standard doses. That is, for these meta‐analyses, we considered RCTs performing head‐to‐head comparisons between AR treatments in standard dose (doses in which the medications are most currently used; Table S3) versus either in different doses or in co‐medication schemes.

### 2.7 Dose–Response Network Meta‐Analysis

We performed a frequentist random‐effects NMA fitting linear dose–response models [13]. We performed main analyses at an individual medication level, considering the whole range of daily doses evaluated for each medication. We evaluated the comparative effect of individual medications per unit of standard daily medication dose (list of standard doses in Table S3). In particular, we presented results for comparisons involving two times the standard dose of intranasal and oral medications for AR in monotherapy versus co‐medication strategies involving the standard dose of the same intranasal and oral medications. For example, we identified primary studies evaluating a standard dose of fluticasone‐propionate + a standard dose of loratadine. We presented results for the comparison between this strategy and (i) double dose of fluticasone propionate, and (ii) double dose of loratadine.

In our main analyses, to ensure a larger number of studies and tested doses, we considered together primary studies performed in adults and children, as well as in patients with perennial and seasonal AR. In addition, we considered “placebo” as our reference category, irrespective of whether it was used intranasally or orally. Nevertheless, we performed sensitivity analyses (i) restricted to adults, (ii) restricted to patients with seasonal AR and (iii) with nasal and oral placebo being considered separate interventions.

## 3 Results

The flow diagram for primary study selection is depicted in Figure S1. The searches conducted in the context of the previous systematic reviews for the ARIA‐EAACI 2024–2025 guidelines have resulted in the identification of 259 different studies (out of a total of 10,301 records screened). In the search update, we retrieved 524 records. After screening 327 deduplicated records, we identified three additional eligible primary studies. Overall, a total of 262 primary studies were included for evidence synthesis, of which 256 were included in meta‐analyses.

A brief description of these studies is available in Table S4. A more detailed description can be found in the previous systematic reviews [7, 8, 9, 11, 12].

### 3.1 Risk of Bias

Figure S2 and Table S5 display the results of the RoB assessment (Figure S3 presents results by subgroup of participants). Overall, 11 (4.2%) studies were classified as having a low risk of bias, 160 (61.8%) had an unclear risk of bias, and 88 (34.0%) a high risk. Most studies had a low risk of bias for “blinding of outcome assessment” and “completeness of outcome data”. On the other hand, due to poor reporting, the majority of studies had unclear risk of bias in “random sequence generation” and “allocation concealment”. The domain most frequently classified as at high risk of bias was that of “selective reporting”.

### 3.2 Meta‐Analytical Results: Pairwise Meta‐Analyses

When considering direct evidence from RCTs comparing different doses of the same medication, we observed mixed results for efficacy outcomes (Table 1A). For several medications, we observed that decreases or increases in medication dose tended to be respectively associated with lower or higher improvements in the assessed outcomes. In particular, this was observed for azelastine, beclomethasone, ciclesonide, ebastine and rupatadine. On the other hand, for other medications (e.g., fluticasone propionate or mometasone), a change in the medication dose was not associated with relevant outcome trends.

**TABLE 1:** Results of the direct comparisons between (A) non‐standard versus standard treatment doses of the same medication, and (B) co‐medication strategies and medications in monotherapy (efficacy outcomes).

A. Comparisons between non‐standard versus standard treatment doses of the same medication				
Medication	Dose comparison^a^	Meta‐analytical results ‐ MD (95% CI) [*p*‐value] [*I* ^2^] [*N* studies; *N* participants]		
		TNSS	TOSS	RQLQ
Azelastine	840 versus 560 μg	−0.02 (−0.48; 0.44) [0.931] [−] [1; 327]	0.05 (−0.27; 0.37) [0.757] [−] [1; 327]	0.08 (−0.12; 0.28) [0.428] [−] [1; 327]
Azelastine	1120 versus 560 μg	−0.28 (−0.67; 0.11) [0.156] [−] [1; 560]	—	—
Beclomethasone	80 versus 320 μg	0.34 (−0.16; 0.84) [0.182] [−] [1; 240]	0.30 (−0.14; 0.74) [0.185] [−] [1; 196]	0.15 (−0.17; 0.47) [0.357] [−] [1; 240]
Beclomethasone	160 versus 320 μg	0.35 (−0.15; 0.85) [0.169] [−] [1; 245]	0.35 (−0.09; 0.79) [0.122] [−] [1; 203]	0.29 (−0.04; 0.62) [0.088] [−] [1; 245]
Ciclesonide	25 versus 200 μg	**0.40 (0.10; 0.70) [0.008] [−] [3; 689]**	—	—
Ciclesonide	50 versus 200 μg	**0.50 (0.03; 0.97) [0.039] [−] [1; 287]**	—	—
Ciclesonide	100 versus 200 μg	0.14 (−0.10; 0.38) [0.240] [−] [4; 1099]	—	—
Ciclesonide	160 versus 200 μg	−0.66 (−1.46; 0.14) [0.108] [−] [1; 108]	—	—
Ciclesonide	320 versus 200 μg	−0.62 (−1.42; 0.18) [0.131] [−] [1; 99]	—	—
Desloratadine	2.5 versus 5 mg	0.60 (−0.24; 1.44) [0.163] [−] [1; 345]	—	—
Desloratadine	7.5 versus 5 mg	−0.10 (−0.94; 0.74) [0.816] [−] [1; 345]	—	—
Desloratadine	10 versus 5 mg	0.20 (−0.64; 1.04) [0.642] [−] [1; 344]	—	—
Desloratadine	20 versus 5 mg	−0.30 (−1.14; 0.54) [0.485] [−] [1; 344]	—	—
Ebastine	20 versus 10 mg	−0.22 (−0.52; 0.08) [0.154] [0%] [3; 776]	—	—
Fexofenadine	120 versus 180 mg	—	—	0.07 (−0.11; 0.25) [0.457] [−] [1; 569]
FF	55 versus 110 μg	0.19 (−0.24; 0.62) [0.378] [36%] [3; 992]	0.07 (−0.25; 0.38) [0.680] [0%] [2; 622]	0.18 (−0.18; 0.54) [0.328] [−] [1; 254]
FF	220 versus 110 μg	**0.65 (0.07; 1.23) [0.029] [−] [1; 256]**	0.16 (−0.30; 0.62) [0.493] [−] [1; 256]	0.14 (−0.21; 0.49) [0.429] [−] [1; 256]
FF	440 versus 110 μg	−0.18 (−0.76; 0.40) [0.545] [−] [1; 257]	−0.35 (−0.82; 0.12) [0.145] [−] [1; 257]	0 (−0.35; 0.35) [1.000] [−] [1; 257]
FP	50 versus 200 μg	0 (−0.66; 0.66) [1.000] [−] [1; 215]	—	—
FP	100 versus 200 μg	−0.21 (−1.14; 0.72) [0.657] [−] [1; 161]	—	—
FP	800 versus 200 μg	0 (−0.67; 0.67) [1.000] [−] [1; 209]	—	—
Mometasone	25 versus 200 μg	0.30 (−0.20; 0.80) [0.241] [−] [1; 270]	—	—
Mometasone	100 versus 200 μg	−0.10 (−0.59; 0.39) [0.689] [−] [1; 268]	—	—
Montelukast	20 versus 10 mg	0.28 (−0.31; 0.87) [0.355] [−] [1; 185]	—	**0.21 (0.06; 0.36) [0.007] [−] [1; 185]**
Olopatadine	2660 versus 5320 μg	0.40 (−0.08; 0.88) [0.101] [−] [1; 319]	—	—
OM	2760 versus 5320 μg	0.10 (−0.46; 0.66) [0.727] [−] [1; 315]	−0.13 (−0.56; 0.30) [0.555] [−] [1; 315]	0.07 (−0.30; 0.44) [0.713] [−] [1; 257]
Rupatadine	20 versus 10 mg	−0.24 (−0.51; 0.03) [0.076] [0%] [3; 875]	—	—
Triamcinolone	110 versus 220 μg	−0.01 (−0.42; 0.40) [0.965] [0%] [3; 456]	0.05 (−0.20; 0.30) [0.694] [−] [1; 147]	—
Triamcinolone	440 versus 220 μg	−0.41 (−0.99; 0.16) [0.161] [0%] [2; 304]	—	—

B. Comparisons between co‐medication strategies versus medications in monotherapy			
Comparison	Meta‐analytical results (95% CI) [*p*‐value] [*I* ^2^] [*N* studies; *N* participants]		
	TNSS	TOSS	RQLQ
Azelastine‐fluticasone versus azelastine	**−0.69 (−1.00; −0.38) [< 0.001] [51%] [5; 2150]**	**−0.30 (−0.59; −0.02) [0.034] [63%] [5; 2148]**	**−0.37 (−0.58; −0.15) [< 0.001] [66%] [5; 2023]**
Azelastine‐fluticasone versus FP	**−0.43 (−0.65; −0.21) [< 0.001] [0%] [4; 2000]**	**−0.32 (−0.55; −0.10) [0.004] [41%] [4; 1998]**	−0.06 (−0.18; 0.05) [0.285] [0%] [4; 1870]
Bilastine + montelukast versus bilastine	0.14 (−0.39; 0.67) [0.606] [−] [1; 283]	0.04 (−0.37; 0.44) [0.861] [−] [1; 283]	—
Bilastine + montelukast versus montelukast	−0.10 (−0.64; 0.43) [0.703] [−] [1; 279]	−0.13 (−0.28; 0.53) [0.546] [−] [1; 279]	—
Ciclesonide + levocetirizine versus ciclesonide	−0.60 (−1.89; 0.69) [0.362] [−] [1; 52]	—	—
Ciclesonide + levocetirizine versus levocetirizine	−1.30 (−2.64; 0.04) [0.058] [−] [1; 45]	—	—
FF + oxymetazoline versus FF	—	—	0.54 (−1.38; 2.46) [0.581] [−] [1; 32]
FF + oxymetazoline versus oxymetazoline	—	—	−0.20 (−2.12; 1.72) [0.838] [−] [1; 33]
FP + loratadine versus FP	−0.48 (−1.10; 0.14) [0.127] [44%] [2; 625]	—	−0.10 (−0.37; 0.17) [0.475] [−] [1; 300]
FP + loratadine versus loratadine	**−1.83 (−3.54; −0.12) [0.036] [93%] [2; 630]**	—	**−1.00 (−1.28; −0.72) [< 0.001] [−] [1; 300]**
Loratadine + montelukast versus loratadine	**−0.59 (−1.01; −0.16) [0.007] [72%] [5; 1782]**	—	−0.11 (−0.24; 0.02) [0.086] [0%] [2; 779]
Loratadine + montelukast versus montelukast	**−0.58 (−0.87; −0.29) [< 0.001] [2%] [4; 1231]**	—	−0.12 (−0.25; 0) [0.058] [0%] [2; 637]
Mometasone + oxymetazoline versus mometasone	—	—	−0.08 (−0.69; −0.53) [0.796] [−] [1; 284]
Mometasone + oxymetazoline versus oxymetazoline	—	—	−0.29 (−0.90; 0.32) [0.350] [−] [1; 285]
Olopatadine‐mometasone versus mometasone	**−0.44 (−0.77; −0.11) [0.010] [25%] [3; 1665]**	**−0.27 (−0.48; −0.06) [0.014] [0%] [2; 1184]**	**−0.21 (−0.37; −0.05) [0.008] [0%] [2; 1189]**
Olopatadine‐mometasone versus olopatadine	**−0.56 (−0.86; −0.26) [< 0.001] [0%] [3; 1504]**	−0.13 (−0.35; −0.08) [0.225] [0%] [2; 1187]	**−0.29 (−0.45; −0.13) [< 0.001] [0%] [2; 1187]**

*Note:* These results concern only direct evidence from head‐to‐head trials comparing, for a same medication, (A) different doses versus the standard dose currently used in practice, (B) its use in co‐medication versus in monotherapy in the standard dose currently used in practice. Bold values indicate “significant” results for a significance level of *p* < 0.05.

Abbreviations: CI, Confidence interval; FF, Fluticasone furoate; FP, Fluticasone propionate; MD, Mean difference (change‐from‐baseline values); OM, Olopatadine‐mometasone; RQLQ, Rhinoconjunctivitis Quality of Life Questionnaire; TNSS, Total Nasal Symptom Score; TOSS, Total Ocular Symptom Score.

^a^ Comparison versus the standard dose of each medication.

Direct evidence from RCTs also indicated that co‐medication schemes were mostly associated with higher improvements in the assessed outcomes than the use of a standard dose of medication in monotherapy (Table 1B). This was particularly observed for INCS + INAH versus INCS or INAH alone, INCS + OAH versus OAH alone. A trend was also observed for INCS + OAH versus INCS alone.

In terms of safety outcomes, for most comparisons, an increase in the frequency of AE or withdrawals due to AE was not observed with the use of higher medication doses or co‐medication (Tables S6 and S7).

### 3.3 Meta‐Analytical Results: Dose–Response NMA

#### 3.3.1 Total Nasal Symptom Score

For the TNSS, we included 191 studies (84,359 participants) in our main analysis. Overall, we were able to provide results for 28 comparisons involving 11 interventions. Quantitative results are described in Figure 1. The certainty of evidence was high for one comparison, moderate for six, low for seven comparisons and very low for 14 (Table S8).

**FIGURE 1:**
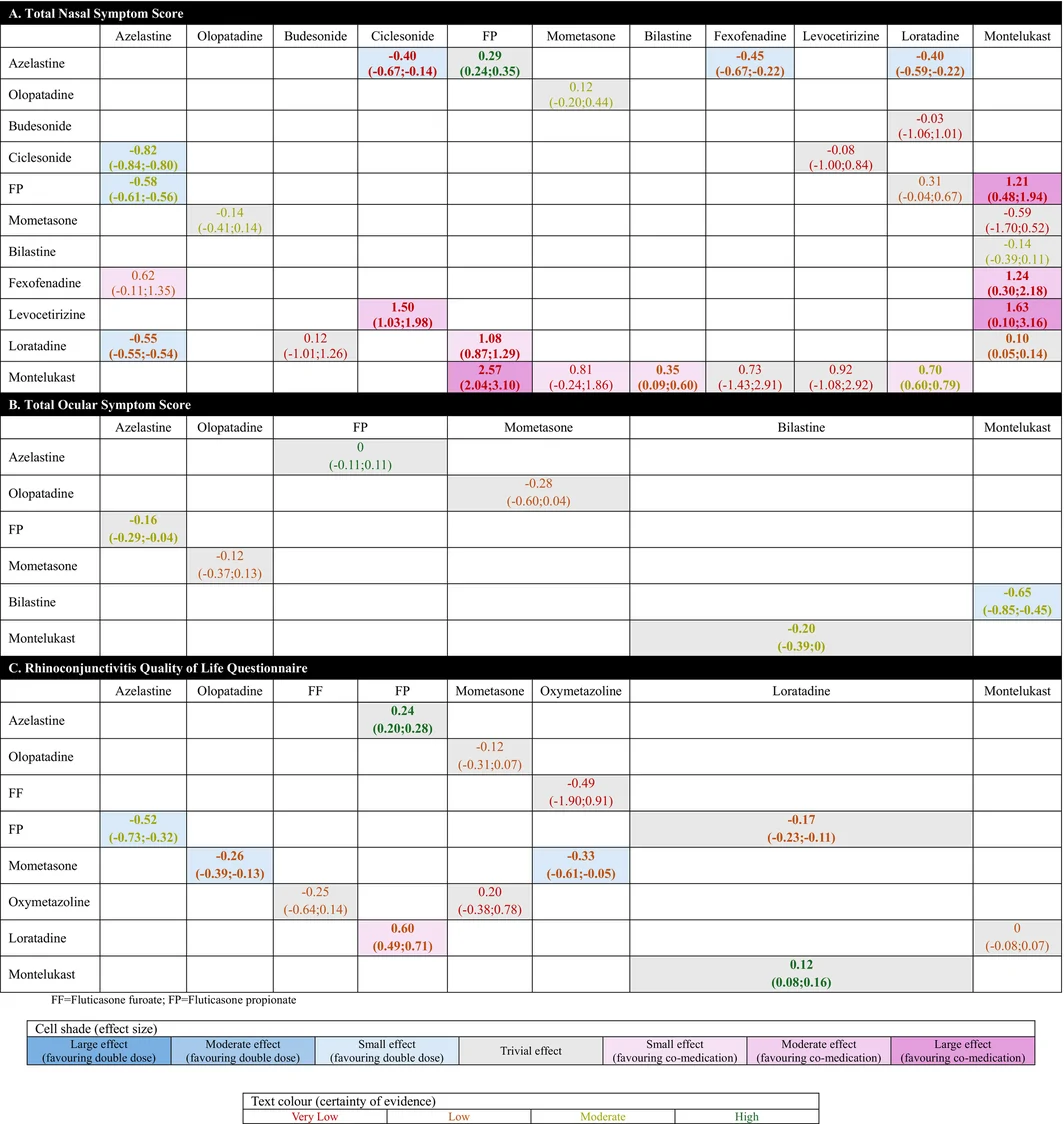
Comparison between doubling the dose of the medication on each row versus adding the medication of each column (main analysis: Randomised controlled trials evaluating patients of all ages and with either seasonal or perennial allergic rhinitis). Results are expressed as mean differences (95% confidence intervals [CI]). Example interpretation of results: For the first non‐empty cell in the first column, a double dose of ciclesonide is associated with a mean difference of −0.82 units (95% CI = −0.84; −0.80) of the Total Nasal Symptom Score compared to ciclesonide + azelastine.

Overall, doubling the dose of an INAH (particularly, azelastine) was associated with improved results compared to adding an OAH (fexofenadine or loratadine), with these differences being small but meaningful. On the other hand, the use of azelastine‐fluticasone or olopatadine‐mometasone was associated with a higher TNSS improvement than doubling the dose of azelastine or olopatadine.

Regarding INCS, doubling their dose resulted in higher TNSS improvement than adding INAH. On the other hand, non‐significant trivial differences were observed for the comparison between doubling the dose of INCS and adding OAH (levocetirizine or loratadine). Mixed results were observed for the comparison between double dose of INCS versus INCS + LTRA.

As for OAH, mixed results were observed for comparisons involving doubling their dose versus adding INAH. On the other hand, co‐medication schemes involving the co‐use of OAH and INCS resulted in overall higher improvements of the TNSS compared to doubling the dose of OAH. These differences were significant and meaningful for the comparisons between levocetirizine + ciclesonide versus double dose of levocetirizine (moderate effect), and between loratadine + fluticasone propionate versus double dose of loratadine (small effect). Co‐medication schemes involving OAH versus LTRA also resulted in improved TNSS compared to doubling the dose of fexofenadine and levocetirizine, but not bilastine.

In terms of LTRA, doubling their dose resulted in worse results compared to adding INCS and OAH. Differences were significant and/or meaningful except for two comparisons (i.e., those involving fexofenadine and levocetirizine) which were very imprecise.

In the sensitivity analysis evaluating patients with seasonal AR (Figure 2), we observed similar results except for the facts that (i) trivial differences were observed between doubling the dose of INCS versus adding OAH to INCS, (ii) there was less available evidence for LTRA, precluding comparisons between double dose of INCS versus INCS + LTRA, and (iii) doubling the dose of OAH resulted in a higher improvement in the TNSS than adding LTRA or in trivial differences (comparisons involving bilastine and loratadine, respectively). Consistent results with the main analysis were observed for the sensitivity analyses restricted to adult patients (Table S9) or separately considering nasal and oral placebo (Table S10).

**FIGURE 2:**
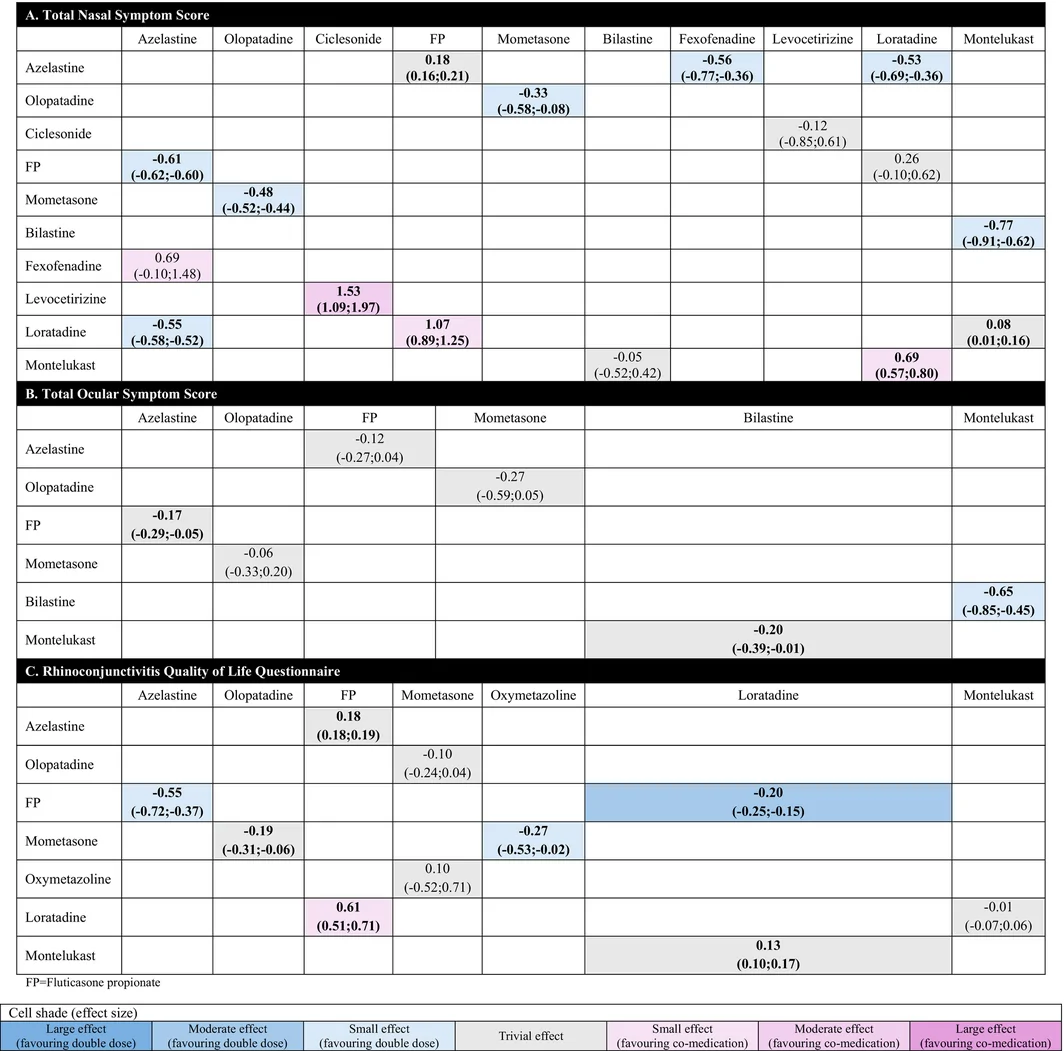
Comparison between doubling the dose of the medication on each row versus adding the medication of each column (sensitivity analysis: Randomised controlled trials evaluating patients with seasonal allergic rhinitis). Results are expressed as mean differences (95% confidence intervals [CI]). Example interpretation of results: For the first non‐empty cell in the first column, a double dose of fluticasone propionate is associated with a mean difference of −0.61 units (95% CI = −0.62; −0.60) of the Total Nasal Symptom Score compared to azelastine + fluticasone. FP, Fluticasone propionate.

#### 3.3.2 Total Ocular Symptom Score

For the TOSS, 44 studies (23,582 participants) were included in the main analysis. We evaluated six comparisons involving six interventions (Figure 1). The certainty of evidence was high for one comparison, moderate for three comparisons and low for two (Table S11).

For most comparisons, we observed trivial differences. The only exception concerned the fact that doubling the dose of bilastine resulted in a larger improvement in the TOSS than adding montelukast. Similar results were observed in the sensitivity analyses restricted to patients with seasonal AR (Figure 2), evaluating only adult patients (Table S9), or separately considering nasal and oral placebo (Table S10).

#### 3.3.3 Rhinoconjunctivitis Quality of Life Questionnaire

For the RQLQ, we included 97 studies (44,689 participants) in our main analysis. We evaluated 12 comparisons involving eight interventions (Figure 1). The certainty of evidence was high for two comparisons, moderate for one comparison, low for seven and very low for two (Table S12).

In terms of intranasal medications, azelastine‐fluticasone resulted in a higher improvement than doubling the dose of azelastine, even though the difference was trivial. On the other hand, doubling the dose of INCS led to better RQLQ results than adding INAH, with that difference being small but meaningful. Doubling the dose of INCS also led to a higher improvement in the RQLQ compared to adding a decongestant or an OAH. Nevertheless, differences were mostly trivial.

Regarding oral medications, adding an INCS to an OAH (in this case, fluticasone propionate to loratadine) was associated with better results than doubling the dose of OAH, with this difference being small but meaningful. Trivial differences were observed when comparing OAH + LTRA versus the double dose of any of these options.

Overall, similar results were observed in sensitivity analyses (Figure 2 and Tables S9 and S10), with a meaningful effect observed when comparing a double dose of fluticasone propionate versus fluticasone propionate + loratadine in patients with seasonal AR.

#### 3.3.4 Adverse Events

In our main analysis, we included 203 studies (84,838 participants) reporting the occurrence of adverse events. We assessed 20 comparisons concerning 11 interventions (Figure 3). In all comparisons except two (double dose of levocetirizine versus levocetirizine + ciclesonide, and double dose of montelukast versus montelukast + bilastine), differences between doubling the medication dose versus adding another medication were trivial. Similar results were observed in sensitivity analyses (Table S13). The certainty of evidence was moderate for eight comparisons, low for five and very low for seven comparisons (Table S14).

**FIGURE 3:**
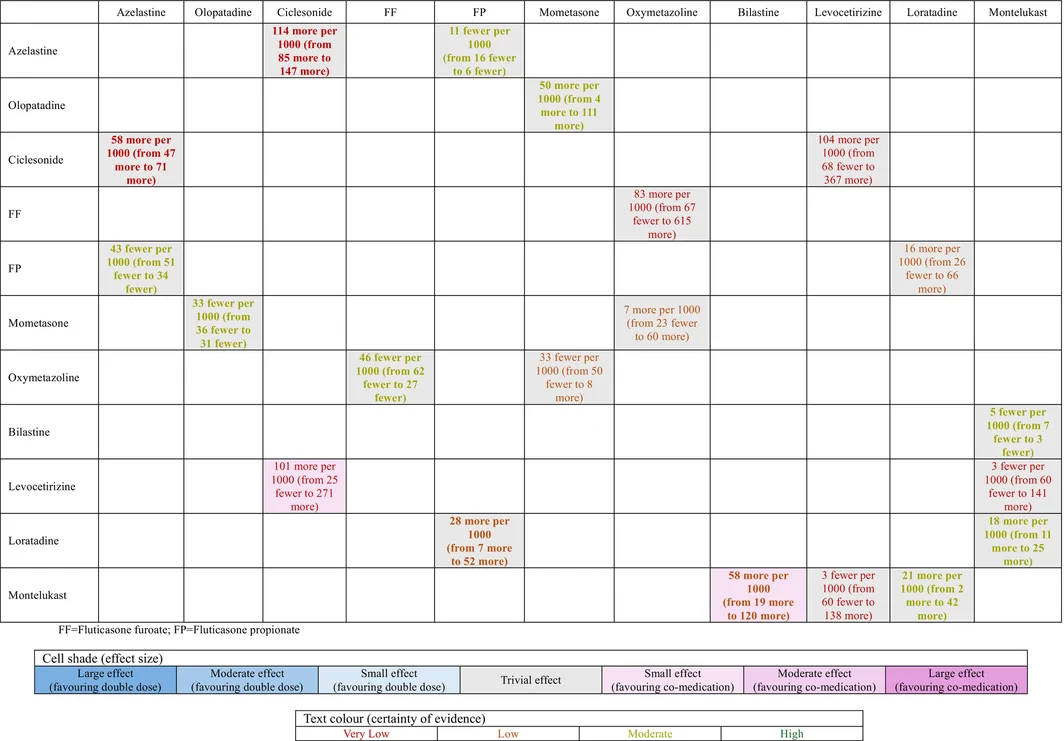
Comparison between doubling the dose of the medication on each row versus adding the medication of each column on the occurrence of adverse events (main analysis: Randomised controlled trials evaluating patients of all ages and with either seasonal or perennial allergic rhinitis). Example interpretation of results: For the first non‐empty cell in the first column, a double dose of ciclesonide is associated with 58 more adverse events per 1000 individuals (from 41 more to 71 more per 1000) compared to ciclesonide + azelastine. FF, Fluticasone furoate; FP, Fluticasone propionate.

#### 3.3.5 Withdrawal Due to Adverse Events

Regarding withdrawal due to adverse events, we included 183 studies (87,148 participants). We evaluated 20 comparisons concerning 11 interventions (Figure S4). For all comparisons, we observed trivial differences when comparing doubling the medication dose versus adding another medication. Consistent results were observed in sensitivity analyses (Table S15). The certainty of evidence was high for three comparisons, moderate for seven comparisons, low for two, and very low for eight (Table S16).

## 4 Discussion

In this systematic review of RCTs on intranasal or oral AR medications, we compared the use of these medications in double dose or co‐medication. We started by performing pairwise meta‐analyses based on direct evidence alone: efficacy results were mixed in terms of dose change and were favourable overall for co‐medication. However, we observed that limited evidence was available, as (i) no comparison included more than five RCTs, (ii) we were not able to find direct evidence for all medications, and (iii) there were no studies directly comparing co‐medication versus treatment dose increase. Therefore, we fitted dose–response NMAs to address our research question. A summary of our results is available in Table 2. In terms of efficacy, we observed that co‐medication schemes involving OAH + INCS generally resulted in higher TNSS and RQLQ improvements than doubling the dose of OAH. On the other hand, when comparing doubling the dose of INCS versus OAH + INCS, we observed that (i) both options displayed similar results in terms of nasal symptoms but (ii) a double dose of INCS was associated with higher RQLQ improvements (particularly when considering only patients with seasonal AR). Doubling the dose of INCS was associated with more favourable TNSS and RQLQ results than INCS + INAH. On the other hand, the comparison between doubling the dose of INAH versus INCS + INAH led to mixed results: overall, fixed combinations of INCS + INAH (in particular, azelastine‐fluticasone) were associated with better efficacy outcomes than doubling the dose of INAH. For non‐fixed combinations, the opposite effect was observed. Finally, as for INCS, doubling the dose of INAH was associated with better results than INAH + OAH. Altogether, these results favour (i) doubling the dose of intranasal medications over adding OAH, and (ii) adding INCS to standard doses of OAH over doubling the dose of OAH. In any case, these efficacy results should be interpreted with caution and consider within‐class medication differences. As for safety, our projected estimates did not point to relevant differences between treatment strategies.

**TABLE 2:** Overall summary of the efficacy results for the comparisons between strategies with use of medication on a double dose versus co‐medication using NMA fitting linear dose–response models.

	TNSS	TOSS	RQLQ
Double dose of INAH versus INAH+INCS	Mixed results^a^	No favoured option	Mixed results^a^
Double dose of INAH versus INAH+OAH	Favours double dose of INAH	No evidence	No evidence
Double dose of INCS versus INCS+INAH	Favours double dose of INCS	No favoured option	Favours double dose of INCS
Double dose of INCS versus INCS+OAH	No favoured option	No evidence	Favours double dose of INCS^b^
Double dose of OAH versus OAH + INAH	Mixed results	No evidence	No evidence
Double dose of OAH versus OAH + INCS	Favours OAH + INCS	No evidence	Favours OAH + INCS

*Note:* Legend of the cell shade values: Blue‐ favours double dose; pink‐ favours co‐medication; purple‐ mixed results; grey‐ no evidence.

Abbreviations: INAH, Intranasal antihistamines; INCS, Intranasal corticosteroids; OAH, Oral antihistamines; RQLQ, Rhinoconjunctivitis quality‐of‐life questionnaire, TNSS, Total nasal symptoms score; TOSS, Total ocular symptom score.

^a^ Overall, fixed combinations of INAH+INCS (particularly azelastine‐fluticasone) were associated with better efficacy outcomes than doubling the dose of INAH; for non‐fixed combinations, the opposite effect was observed.

^b^ Particularly considering results for patients with seasonal allergic rhinitis.

Importantly, while this systematic review has compared two treatment strategies based on their efficacy and safety, there are other criteria that need to be considered for decision‐making. At a societal level, these include, among others, availability, cost‐effectiveness and planetary health impact of interventions [24, 25, 26]. At an individual patient level, patients' values also need to be taken into account, with the consideration of aspects such as onset of treatment action, acceptability, taste, corticosteroid‐phobia, or affordability [27]. Therefore, while this systematic review will inform the ARIA‐EAACI 2024–2025 guidelines on the efficacy and safety (“desirable and undesirable effects”) of the different strategies, its results do not constitute a treatment recommendation, as they need to be put in perspective of a set of contextual factors.

This systematic review has some limitations worth noting. First, not all medications have primary studies assessing them in co‐medication with other treatments. Therefore, we were only able to test a limited number of comparisons. As a result, there are analyses for which some treatment classes were represented by a single medication. For example, for meta‐analyses on RQLQ, loratadine was the only OAH we were able to evaluate, as it was the medication tested in all studies assessing OAH in co‐medication. While it would have been possible to obtain projections for a double dose of a certain medication versus a co‐medication scheme involving a different medication (e.g., double dose of cetirizine versus loratadine + fluticasone), such comparisons were deemed not as relevant and informative for clinical practice as they do not reflect questions that appear frequently in daily practice. An additional limitation concerns the fact that there are medications assessed only in a single dose or in a limited set of doses. This has led to indirectness concerns for the projected estimates obtained for some comparisons. In addition, considering this constraint, we were not able to test whether non‐linear models would be a better fit for our dose–response NMA. Finally, most studies had a high or unclear risk of bias, not describing with sufficient detail aspects such as how randomisation or blinding were implemented.

This study also has important strengths. First, this systematic review addresses a relevant question. Several observational studies have indicated that patients treat themselves according to the severity of their symptoms [4, 5, 28]. In fact, patients seem to frequently resort to co‐medication when feeling insufficiently well controlled, with a study having observed that almost three fourths of the patients had reported at least 1 day in co‐medication [4]. The same study found that it is common for patients to try two or more different medications per year, including different medications of the same class [4]. Considering the relevance of the question it addresses, this systematic review will inform the ARIA‐EAACI 2024–2025 guidelines, in particular the question “Should co‐medication vs. medication updosing be used in patients with allergic rhinitis that is poorly controlled despite pharmacologic treatment?” [14, 15]. Importantly, to the best of our knowledge, this will be the first guideline on rhinitis to address this question. In contradistinction, for other allergic diseases like urticaria, updosing and co‐medication strategies are a key part of guidelines [3]. Second, to test the robustness of our results, we have performed several sensitivity analyses, including (i) for patients with seasonal allergic rhinitis, (ii) for adult patients, and (iii) considering nasal and oral placebo separately. For most comparisons, we observed similar results in the main analysis and in the sensitivity analyses. Finally, in order to overcome some of the limitations of traditional NMA methods, we have performed a dose–response NMA, the first in AR and one of the first in the allergy field [29]. Traditional methods would imply that we would consider each individual set of treatment‐dose as a different NMA node, resulting in (i) weakly connected NMAs and imprecise estimates, and (ii) incapacity to obtain projections for doses that have not been evaluated in primary studies [13, 23, 30].

In addition to its implications for clinical practice, this systematic review has important implications for research. The main implications are summarised in Table 3 and Table S17. An additional implication concerns the need to have a specific framework for assessing the certainty of evidence in the context of dose–response NMA. The special considerations that we adopted when evaluating the certainty of evidence (e.g., assuming that a comparison is defined as a pair of treatments and of their respective doses or considering the intervention doses when rating indirectness and intransitivity) can support a subsequent discussion for future GRADE guidance or for a concept paper on the subject.

**TABLE 3:** Summary of the implications for research of the systematic review.

Outcomes	Thresholds	Certainty of evidence	Lower certainty of evidence (*N* downgraded comparisons/*N* comparisons)	Implications for future research
Nasal symptoms	−0.8/−0.5/−0.2/0.2/0.5/0.8	Number of comparisons according to the certainty of evidence:—High: 1—Moderate: 6—Low: 7—Very low: 14	*N* comparisons with downgrading per domain: Risk of bias: 9/20 Indirectness: 18/20 Inconsistency: 3/20 Imprecision: 18/28 Dissemination bias: 0/20 Intransitivity: 0/24 Incoherence: 7/16	*Risk of bias*: Future research may make available their protocols and report complete data for all evaluated outcomes. *Indirectness*: Future trials may perform direct comparisons between increased dose of medication in monotherapy versus these treatments in co‐medication. *Inconsistency*: Future trials may be performed in children or patients with perennial allergic rhinitis to assess a potential subgroup effect associated with these variables. Results stratified by the presence of other variables (e.g., comorbid asthma) are needed. *Imprecision*: Future adequately powered trials are needed, particularly directly comparing increased dose of medication in monotherapy versus co‐medication strategies. *Incoherence*: Future trials are needed addressing direct comparisons and comparisons that are part of the most dominant first order loop in order to allow an identification of factors that may have resulted in differences between direct and indirect evidence.
Ocular symptoms	−0.8/−0.5/−0.2/0.2/0.5/0.8	Number of comparisons according to the certainty of evidence:—High: 1—Moderate: 3—Low: 2—Very low: 0	*N* comparisons with downgrading per domain: Risk of bias: 0/6 Indirectness: 6/6 Inconsistency: 0/6 Imprecision: 2/6 Dissemination bias: 0/6 Intransitivity: 0/6 Incoherence: 1/6	*Indirectness*: Future trials may perform direct comparisons between increased dose of medication in monotherapy versus these treatments in co‐medication. *Imprecision*: Future adequately powered trials are needed, particularly directly comparing increased dose of medication in monotherapy versus co‐medication strategies. *Incoherence*: Future trials are needed addressing direct comparisons and comparisons that are part of the most dominant first order loop in order to allow an identification of factors that may have resulted in differences between direct and indirect evidence.
Rhinoconjunctivitis‐related quality of life	−0.8/−0.5/−0.2/0.2/0.5/0.8	Number of comparisons according to the certainty of evidence:—High: 2—Moderate: 1—Low: 7—Very low: 2	*N* comparisons with downgrading per domain: Risk of bias: 4/12 Indirectness: 9/12 Inconsistency: 1/12 Imprecision: 6/12 Dissemination bias: 0/12 Intransitivity: 0/12 Incoherence: 2/12	*Risk of bias*: Future research may make available their protocols and report complete data for all evaluated outcomes. *Indirectness*: Future trials may perform direct comparisons between increased dose of medication in monotherapy versus these treatments in co‐medication. *Inconsistency*: Future trials may be performed in children or patients with perennial allergic rhinitis to assess a potential subgroup effect associated with these variables. Results stratified by the presence of other variables (e.g., comorbid asthma) are needed. *Imprecision*: Future adequately powered trials are needed, particularly directly comparing increased dose of medication in monotherapy versus co‐medication strategies. *Incoherence*: Future trials are needed addressing direct comparisons and comparisons that are part of the most dominant first order loop in order to allow an identification of factors that may have resulted in differences between direct and indirect evidence.
Adverse events	Thresholds per 1000 persons:—INAH: −862/−442/−187/187/442/862—INCS: −637/−327/−128/128/327/637—OAH: −303/−161/−65/65/161/303	Number of comparisons according to the certainty of evidence:—High: 0—Moderate: 8—Low: 5—Very low: 7	*N* comparisons with downgrading per domain: Risk of bias: 7/18 Indirectness: 18/18 Inconsistency: 2/18 Imprecision: 6/20 Dissemination bias: 0/18 Intransitivity: 0/17 Incoherence: 4/17	*Risk of bias*: Future research may make available their protocols and report data for all evaluated outcomes. *Indirectness*: Future trials may perform direct comparisons between increased dose of medication in monotherapy versus these treatments in co‐medication. *Inconsistency*: Future trials may be performed in children or patients with perennial allergic rhinitis to assess a potential subgroup effect associated with these variables. Results stratified by the presence of other variables (e.g., comorbid asthma) are needed. *Imprecision*: Future adequately powered trials are needed, particularly directly comparing increased dose of medication in monotherapy versus co‐medication strategies. *Incoherence*: Future trials are needed addressing direct comparisons and comparisons that are part of the most dominant first order loop in order to allow an identification of factors that may have resulted in differences between direct and indirect evidence.
Withdrawal due to adverse events	Thresholds per 1000 persons: ‐ INAH: −862/−442/−187/187/442/862—INCS: −637/−327/−128/128/327/637—OAH: −303/−161/−65/65/161/303	Number of comparisons according to the certainty of evidence:—High: 3—Moderate: 7—Low: 2—Very low: 8	*N* comparisons with downgrading per domain: Risk of bias: 6/17 Indirectness: 16/17 Inconsistency: 0/17 Imprecision: 2/20 Dissemination bias: 0/17 Intransitivity: 0/16 Incoherence: 0/16	*Risk of bias*: Future research may make available their protocols and report data for all evaluated outcomes. *Indirectness*: Future trials may perform direct comparisons between increased dose of medication in monotherapy versus these treatments in co‐medication. *Imprecision*: Future adequately powered trials are needed, particularly directly comparing increased dose of medication in monotherapy versus co‐medication strategies. *Incoherence*: Future trials are needed addressing direct comparisons and comparisons that are part of the most dominant first order loop in order to allow an identification of factors that may have resulted in differences between direct and indirect evidence.
Across outcomes summary	NA	Number of comparisons according to the certainty of evidence:—High: 7—Moderate: 25—Low: 23—Very low: 31	*N* comparisons with lower certainty of evidence: Risk of bias: 26 Indirectness: 67 Inconsistency: 6 Imprecision: 34 Dissemination bias: 0 Intransitivity: 0 Incoherence: 14	‐ Future studies should pre‐register and make available their protocols and report complete data for all the evaluated outcomes—More and larger future studies need to be conducted, particularly performing direct comparisons between medications in double dose versus *co‐medication strategies* ‐ Future studies may be conducted in children and patients with perennial allergic rhinitis. In addition, studies may present result stratified by the presence of demographic and clinical variables

In conclusion, in this systematic review with dose–response NMA, we evaluated RCTs of intranasal or oral medications in patients with AR. We projected estimates on efficacy and safety outcomes for the comparisons between increasing the medication dose or adding a different medication. Overall, in terms of efficacy, our results favour (i) doubling the dose of intranasal medications versus adding OAH, and (ii) adding INCS to standard doses of OAH over a double dose of OAH. Regarding strategies involving only intranasal medications, mixed results were observed. As for safety, differences between the two strategies were trivial for most studied comparisons. Our results will inform recommendations of the ARIA‐EAACI 2024–2025 guidelines, alongside information on other contextual factors such as acceptability, availability or costs.

## Author Contributions

Bernardo Sousa‐Pinto participated in conceptualization, methodology, data collection, data analysis and writing original draft. Rafael José Vieira, Sara Gil‐Mata, Ana Margarida Pereira, Vítor Henrique Duarte, Manuel Marques‐Cruz and Alexandro W.L. Chu participated in methodology, data collection, and manuscript review and editing. Antonio Bognanni, Paweł Jemioło, Mattia Giovannini, Danilo di Bona, Arnav Agarwal, Mark S. Dykewicz, Mohamed H. Shamji, Maria Jose Torres, Torsten Zuberbier, Ludger Klimek, Holger J. Schünemann and João A. Fonseca participated in methodology and manuscript review and editing. Guido Schwarzer participated in methodology, data analysis, and manuscript review and editing. Jean Bousquet participated in conceptualization, supervision, and manuscript review and editing.

## Funding

The work of this study has been funded within the context of the Allergic Rhinitis and its Impact on Asthma (ARIA) group.

## Conflicts of Interest

J.B. reports personal fees from Cipla, Menarini, Mylan, Novartis, Purina, Sanofi‐Aventis, Teva, Noucor, other from KYomed‐Innov, and other from Mask‐air‐SAS, outside the submitted work. J.A.F. reports grants from Astrazeneca and Mundipharma; and personal fees from AstraZeneca, Mundipharma, Sanofi, GSK and Teva outside the submitted work. L.K. reports personal fees from Allergopharma, personal fees from Viatris, personal fees from LETI Pharma, personal fees from Stallergenes, personal fees from ALK Abelló, personal fees from HAL Allergie, personal fees from Sanofi, personal fees from AstraZeneca, personal fees from GSK, personal fees from Inmunotek, personal fees from Regeneron Pharmaceuticals, personal fees from Novartis, outside the submitted work; and Membership: AeDA, DGHNO, Deutsche Gesellschaft für Allergologie und klinische Immunologie, HNO‐BV, GPA, EAACI. M.J.T. reports personal fees from Leti Laboratories, personal fees from Aimmune Therapeutics, personal fees from Diater Laboratories, grants from European Commission, grants from ISCIII, grants from SEAIC outside the submitted work. T.Z. reports personal fees from Amgen, personal fees from AstraZeneca, personal fees from AbbVie, personal fees from ALK‐Abelló, personal fees from Almirall, personal fees from Astellas, personal fees from Bayer Health Care, personal fees from Bencard, personal fees from Berlin Chemie, personal fees from FAES Farma, personal fees from HAL Allergie GmbH, personal fees from Henkel, personal fees from Kryolan, personal fees from Leti, personal fees from L'Oreal, personal fees from Meda, personal fees from Menarini, personal fees from Merck Sharp & Dohme, personal fees from Novartis, personal fees from Nuocor, personal fees from Pfizer, personal fees from Sanofi, personal fees from Stallergenes, personal fees from Takeda, personal fees from Teva, personal fees from UCB, personal fees from Uriach, personal fees from Abivax, personal fees from Blueprint, personal fees from Celldex, personal fees from Celltrion, outside the submitted work; and Committee member, “Allergic Rhinitis and its Impact on Asthma” (ARIA), Member of the Board, German Society for Allergy and Clinical Immunology (DGAKI), Head, European Centre for Allergy Research Foundation (ECARF), President, Global Allergy and Asthma Excellence Network (GA^2^LEN), and Member, Committee on Allergy Diagnosis and Molecular Allergology, World Allergy Organisation (WAO). M.G. reports personal fees from Sanofi, Thermo Fisher Scientific, outside the submitted work. The other authors have nothing to disclose, outside the submitted work.

## Supporting information


**Figure S1:** Flow diagram for primary study selection.
**Figure S2:** Risk of bias graph considering all included randomised controlled trials.
**Figure S3:** Risk of bias graphs for studies in (A) adults with perennial allergic rhinitis (*N* = 62), (B) adults with seasonal allergic rhinitis (*N* = 162), (C) children with perennial allergic rhinitis (*N* = 22) and (D) children with seasonal allergic rhinitis (*N* = 13).
**Figure S4:** Comparison between doubling the dose of the medication on each row to adding the medication of each column on the occurrence of withdrawal due to adverse events (main analysis: randomised controlled trials evaluating patients of all ages and with either seasonal or perennial allergic rhinitis). Example interpretation of results: for the first non‐empty cell in the first column, a double dose of ciclesonide is associated with 2 more withdrawals due to adverse events per 1000 individuals (from 3 fewer to 11 more per 1000) compared to ciclesonide + azelastine.
**Box S1**. PRISMA NMA Checklist of Items to Include When Reporting A Systematic Review Involving a Network Meta‐analysis.
**Table S1:** Queries applied in the different bibliographic databases.
**Table S2:** Decision thresholds adopted to evaluate the certainty of the evidence for the different outcomes.
**Table S3:**. Standard daily doses in adult patients that were considered for the different individual medications.
**Table S4:** Description of the primary studies included in the systematic review.
**Table S5:** Results of the risk of bias assessment for each included primary study.
**Table S6:** Results of the direct comparisons between non‐standard versus standard treatment doses of the same medication: Safety outcomes. These results concern only direct evidence from head‐to‐head trials comparing, for a same medication, different doses versus the standard dose currently used in practice.
**Table S7:** Results of the direct comparisons between co‐medication strategies and medications in monotherapy: Safety outcomes. These results concern only direct evidence from head‐to‐head trials comparing, for a same medication, its use in co‐medication versus in monotherapy in the standard dose currently used in practice.
**Table S8:** Certainty of Evidence assessment for the comparisons on the total nasal symptom score.
**Table S9:** Comparison between doubling the dose of the medication on each row to adding the medication of each column (sensitivity analysis: randomised controlled trials evaluating adult patients). Results are expressed as mean differences (95% confidence intervals [CI]). Example interpretation of results: for the first non‐empty cell in the first column, a double dose of ciclesonide is associated with a mean difference of −0.92 units (95% CI = −0.99; −0.85) of the Total Nasal Symptom Score compared to ciclesonide + azelastine.
**Table S10:** Comparison between doubling the dose of the medication on each row to adding the medication of each column (sensitivity analysis: randomised controlled trials differentiating nasal and oral placebo). Results are expressed as mean differences (95% confidence intervals [CI]). Example interpretation of results: for the first non‐empty cell in the first column, a double dose of ciclesonide is associated with a mean difference of −0.16 units (95% CI = −0.20; −0.12) of the Total Nasal Symptom Score compared to ciclesonide + azelastine.
**Table S11:** Certainty of Evidence assessment for the comparisons on the total ocular symptom score.
**Table S12:** Certainty of Evidence assessment for the comparisons on the rhinoconjunctivitis quality‐of‐life questionnaire.
**Table S13:** Comparison between doubling the dose of the medication on each row to adding the medication of each column on the occurrence of adverse events (main and sensitivity analyses). Results are expressed as risk ratios (95% confidence intervals [CI]). Example interpretation of results: for the first non‐empty cell in the first column, a double dose of ciclesonide is associated with 1.46 times (95% CI = 1.37; 1.56) increase in the risk of adverse events compared to ciclesonide + azelastine.
**Table S14:** Certainty of Evidence assessment for the comparisons on adverse events.
**Table S15:** Comparison between doubling the dose of the medication on each row to adding the medication of each column on the occurrence of withdrawal due to adverse events (main and sensitivity analyses). Results are expressed as risk ratios (95% confidence intervals [CI]). Example interpretation of results: for the first non‐empty cell in the first column, a double dose of ciclesonide is associated with 1.20 times (95% CI = 0.72; 2.02) increase in the risk of adverse events compared to ciclesonide + azelastine.
**Table S16:** Certainty of Evidence assessment for the comparisons on withdrawals due to adverse events.
**Table S17:** Full description of the research implications of the systematic reviews.

## Data Availability

The data that support the findings of this study are available from the corresponding author upon reasonable request.
