# Supplementary material for: Treatment Dose Increase Versus Co‐Medication in Allergic Rhinitis: Systematic Review With Dose–Response Network Meta‐Analysis
Source: Allergy. 2026 May 1;81(11):3797–812. doi: 10.1111/all.70372 (PMC13753678; doi:10.1111/all.70372)
Supplement: Supplementary file 1 [file ALL-81-3797-s001.zip › all70372sup-0001-Supinfo.DOC]

**Supplementary Methods**

We performed a systematic review with dose-response NMA of RCTs evaluating intranasal or oral medications in patients with AR. This systematic review follows the Preferred Reporting Items for Systematic Reviews and Meta-Analyses (PRISMA) extension for NMA (PRISMA-NMA)^1^. Its protocol has been published^2^ and has been registered in PROSPERO (CRD420251113186).

**Eligibility criteria**

We included RCTs with a parallel design assessing patients of any age with seasonal or perennial AR and evaluating the effect of (i) intranasal medications (intranasal corticosteroids, intranasal antihistamines, or fixed combinations of intranasal antihistamines + corticosteroids), (ii) oral medications (oral antihistamines or leukotriene receptor antagonists), or (iii) combinations of any intranasal and/or oral medications for AR. We considered both studies in which these interventions were compared with placebo as well as those in which active interventions were compared among themselves. We included studies in which the aforementioned interventions were evaluated irrespectively of the treatment dose.

In line with the outcomes prioritized by the ARIA 2024-2025 guideline panel^3,4^, we included studies reporting results on at least one of the following patient-reported outcome measures: Total Nasal Symptom Score (TNSS), Total Ocular Symptom Score (TOSS), or Rhinoconjunctivitis Quality of Life Questionnaire (RQLQ). We defined the TNSS as any score computed based on the sum of four patient-reported scores for individual nasal symptoms (sneezing, nasal itching, rhinorrhea, and nasal congestion), and the TOSS as any score computed based on the sum of three patient-reported scores for individual ocular symptoms (ocular itching, ocular redness and ocular watering/tearing). We considered the TNSS, TOSS, and RQLQ assessed in a reflective manner; that is, reflecting patients’ symptoms in the previous 12 or 24 hours. Considering Food and Drug Administration recommendations, we only included RCTs with a follow-up period of at least 2 weeks if assessing patients with seasonal AR or at least 4 weeks if assessing patients with perennial AR^5^.

We did not exclude studies based on publication language, date, or status (i.e., we included relevant studies irrespective of whether they were published as full papers, conference abstracts, clinical trials registries, or others).

**Information sources and search strategy**

We included the primary studies that have been included in our previous systematic reviews and that meet the eligibility criteria^6-11^. In brief, we have conducted six previous systematic reviews in which we searched for RCTs evaluating intranasal^6-8,10^ or oral medications^6,9,11^ in adult or pediatric patients with seasonal or perennial AR. The information sources and search strategies for these systematic reviews are described in the respective publications^6-11^. In detail, we searched four bibliographic databases (MEDLINE and Embase via Ovid, Web of Science, and the Cochrane Central Register of Controlled Trials) as well as databases of clinical trials (clinicaltrials.gov, the GSK clinical study dataset, and the AstraZeneca Clinical Trials Website). We updated our search in July 2025, by applying a search strategy based on the ones adopted in our previous systematic reviews in the same bibliographic sources (Supplementary Table 1).

**Study selection and data collection**

Study selection was independently performed by two researchers, first by title and abstract screening, and then by full text reading. Each non-excluded record was carefully assessed to identify multiple publications originating from the same study and avoid including the same participants more than once.

The following variables were independently extracted by two reviewers from each newly included primary study: (i) the assessed disease (seasonal or perennial AR), (ii) the participants’ eligibility criteria, (iii) the data collection period, (iv) the places from where patients were recruited, (v) the follow-up period, (vi) the assessed medications, (vii) the total daily dose of medications, (viii) the number of randomized participants, (ix) the number of participants completing the study, (x) the participants’ age and sex distribution, and (xi) the assessed outcomes. For each desirable effects outcome (TNSS, TOSS, and/or RQLQ), we retrieved (i) information on the scale and computation methods, as well as (ii) baseline values and post-intervention and/or change from baseline values. We also retrieved information on undesirable outcomes, namely on the frequency of patients (i) developing at least one adverse event (AE) (as defined by the authors), (ii) developing at least one serious AE, and (iii) discontinuing treatment due to AE. When results were only provided in a graphical form, estimates were obtained using the PlotDigitizer tool.

Disagreements between reviewers in the data selection or extraction processes were resolved by consensus or by a third reviewer.

**Risk of bias and certainty of evidence assessment**

Two researchers independently evaluated the risk of bias of each included primary study (at an outcome level) using the Cochrane risk-of-bias tool^12^. Disagreements were resolved by consensus or by a third reviewer. The small amount of RCTs with a low risk of bias precluded a sensitivity analysis restricted to those studies.

We evaluated the certainty of the evidence at an outcome level based on the GRADE approach for NMA^13,14^. We followed the most recent GRADE guidance^15^, considering predefined decision thresholds to judge limitations in the design or execution of studies (“risk of bias”), inconsistency, indirectness and imprecision. For continuous outcomes, we considered that standardized mean differences of 0.2, 0.5 and 0.8 can be used as decision thresholds respectively distinguishing “trivial or none” from “small” effects, “small” form “moderate” effects, and “moderate” from “large” effects. These thresholds have been classically used to contextualize standardized mean differences^16^. Of note, the adoption of these thresholds implied that we rated the certainty of evidence for meta-analytical estimates calculated as standardized mean differences, even though our main meta-analytical results were obtained and reported as mean differences. For dichotomous outcomes, we considered previously determined decision thresholds for AEs^11,17^ (Supplementary Table 2). Considering the specificities of dose-response NMA, we adopted some special considerations when evaluating the certainty of evidence. In particular, (i) we assumed that a “comparison” is defined not only as a pair of treatments but rather as a pair of treatments and the respective doses, and (ii) we considered the doses of interventions evaluated in included primary studies when rating indirectness and intransitivity (i.e., we would rate down for indirectness if the doses of interest had never been evaluated in any primary study).

**Quantitative synthesis of the evidence**

Desirable outcomes are continuous and were presented as mean (± standard deviation) baseline and change-from-baseline values. When information on spread measures was missing, we estimated them based on an approach proposed by Weir et al.^18^ as described and applied in our previous systematic reviews^6-11^. Undesirable outcomes are dichotomous and were presented using absolute and relative frequencies.

Both for pairwise and network approaches, we performed meta-analysis of (i) mean differences in change-from-baseline values for desirable effects outcomes (TNSS, TOSS and RQLQ), and (ii) risk ratios for undesirable effects outcomes (AE, withdrawal due to AE). We did not perform meta-analysis on the frequency of serious AE due to the scarce number of events.

All analyses were performed using software R. The meta package was used for pairwise meta-analysis. The netdose package was used for dose-response network meta-analysis^19^.

**Pairwise meta-analysis**

As first steps in our analysis, we performed frequentist pairwise random-effects meta-analysis comparing (i) standard versus other doses of AR medications, and (ii) co-medication versus monotherapy in standard doses. That is, for these meta-analyses, we considered RCTs performing head-to-head comparisons between AR treatments in standard dose (doses in which the medications are most currently used; Supplementary Table 3) *versus* either in different doses or in co-medication schemes. We used the restricted maximum likelihood estimator for between-study variance. Heterogeneity was evaluated using the *I*^2^ statistic.

**Dose-response network meta-analysis**

We performed a frequentist dose-response NMA^20^. Main analyses were performed at an individual medication level, considering the whole range of daily doses which have been evaluated for each medication. Considering that most medications have only been evaluated on a limited set of daily doses, we fitted linear dose-response models (i.e., such limited evidence may render more difficult to have evidence supporting the application of more complex models).

We evaluated the comparative effect of individual medications per unit of standard daily medication dose. For example, for loratadine, adults are usually treated with a daily dose of 10 mg. Therefore, 10 mg corresponds to the standard daily medication dose for loratadine (having been coded as “1” in our models); a study evaluating a dose of 20 mg would be assessing two times the standard daily medication dose for loratadine (such dose would be coded as “2” in our models). On the other hand, adults are usually treated with 200 μg of fluticasone propionate, so that this was considered the standard daily dose for this medication. The list of standard daily doses that we considered in our systematic review is displayed in Supplementary Table 3. The justification for considering units of standard daily medication doses is grounded on the dose differences in intranasal medications (typically daily doses of tens to hundreds of micrograms of active compound) *vis-à-vis* oral medications (typically daily doses of tens to hundreds of milligrams). Considering standard daily medication doses allowed for enhanced comparability in results presentation.

Whenever dealing with interventions corresponding to co-medication schemes, we considered the standard daily dose for adults of each of the medications that are part of such scheme. For example, an intervention corresponding to 10 mg of loratadine + 5 mg of montelukast was not coded as corresponding to a daily dose of 1.5 (1 standard daily dose of loratadine + 0.5 standard daily dose of montelukast) but rather – in different models – as 1 and 0.5. Coding as “1” informs if increasing the dose of loratadine is more or less effective than adding montelukast; coding as “0.5” informs if increasing the dose of montelukast is more or less effective than adding loratadine.

In our main analyses, we considered “placebo” as our reference category, assuming a dose of 0 for placebo interventions. Nasal and oral placebo were not distinguished in our main analyses, as there was only one primary study directly comparing these two placebo formulations: separating nasal *versus* oral placebo would result in a weak connection between the subnetwork evaluating nasal interventions and the subnetwork evaluating oral interventions. Nevertheless, and considering the potential effect of nasal placebo in cleaning the nasal cavities, we performed sensitivity analyses distinguishing nasal and oral placebo. In those sensitivity analyses, nasal placebo was considered an active intervention with a dose of 1, while oral placebo was considered the reference category, displaying a dose of 0.

In our main analyses, we considered together studies performed in adults and children, as well as in patients with perennial and seasonal AR. Sensitivity analyses were performed for studies (i) assessing adults, and (ii) restricted to patients with seasonal AR.

The transitivity assumption (i.e., the existence of comparable distributions of patient characteristics across studies in the treatment network) was assessed by considering patient and study characteristics across the studies that compare pairs of treatments and the respective doses. We assessed heterogeneity at a comparison level by comparing the number of decision thresholds crossed by the random-effects *versus* the fixed-effects model, in accordance with the GRADE guidance^15^. We evaluated incoherence between direct and indirect evidence using a splitting approach. Namely, for each comparison, we compared the predicted estimates when considering only direct evidence versus when removing direct evidence (indirect evidence based on the rest of the network).

We presented results using net league tables. In dose-response NMA, there is an unlimited set of comparisons that can be performed. In fact, for the same pair of interventions, different projected meta-analytical estimates can be obtained depending on the doses being considered for each treatment. Therefore, it is necessary to specify the comparisons (pairs of treatments and of the respective doses) for which results are presented. In this dose-response NMA, we presented results for comparisons involving two times the standard dose of intranasal and oral medications for AR in monotherapy *versus* co-medication strategies involving the standard dose of the same intranasal and oral medications. For example, we identified primary studies evaluating a standard dose of fluticasone-propionate + a standard dose of loratadine. We presented results for the comparison between this strategy and (i) double dose of fluticasone propionate, and (ii) double dose of loratadine.

**Changes to the initial protocol**

In contradistinction to what was initially planned, we grouped together primary studies performed in adults and in children in our main analyses. This was justified by the scarcity of studies performed in children. This factor, as well as the scarcity of studies performed in patients with perennial AR, led us to opt for sensitivity analyses in adults and in patients with seasonal AR (instead of subgroup analyses by type of AR and by age group). We also performed additional sensitivity analyses distinguishing nasal and oral placebo as separate interventions.

Finally, although not described in the initial protocol, we have performed pairwise meta-analysis comparing (i) standard versus other doses of AR medications, and (ii) co-medication versus monotherapy in standard doses. We opted to perform these analyses in order to allow for a better contextualization of the dose-response NMA results, demonstrating what is the current direct evidence from head-to-head trials. Of note, these analyses or their results did not imply any change to the procedures described in the protocol.

**Supplementary Table 1. Queries applied in the different bibliographic databases**

| **MEDLINE (Ovid)** | | | |
| --- | --- | --- | --- |
| 1 | | azelastin$.mp. | |
| 2 | | azelastine-fluticasone.mp. | |
| 3 | | azeflu.mp. | |
| 4 | | MP-azeflu.mp. | |
| 5 | | (azelastine adj2 fluticasone).mp. | |
| 6 | | dymista.mp. | |
| 7 | | azecort.mp. | |
| 8 | | olopatadin$.mp. | |
| 9 | | olopatadine-mometasone.mp. | |
| 10 | | (olopatadine adj2 mometasone).mp. | |
| 11 | | gsp301.mp. | |
| 12 | | ryaltris.mp. | |
| 13 | | levocabastin$.mp. | |
| 14 | | lexicomp.mp. | |
| 15 | | ciclesonid$.mp. | |
| 16 | | Alvesco.mp. | |
| 17 | | fluticason$.mp. | |
| 18 | | GW685698X.mp. | |
| 19 | | mometason$.mp. | |
| 20 | | beclometason$.mp. | |
| 21 | | budesonid$.mp. | |
| 22 | | triamcinolon$.mp. | |
| 23 | | azelastine.nm. | |
| 24 | | levocabastine.nm. | |
| 25 | | ciclesonide.nm. | |
| 26 | | acrivastine$.mp. | |
| 27 | | alimemazine$.mp. | |
| 28 | | antazoline$.mp. | |
| 29 | | astemizole$.mp. | |
| 30 | | bilastine$.mp. | |
| 31 | | brompheniramine$.mp. | |
| 32 | | carbinoxamine$.mp. | |
| 33 | | cetirizine$.mp. | |
| 34 | | chlorpheniramine$.mp. | |
| 35 | | clemastine$.mp. | |
| 36 | | cetrizine$.mp. | |
| 37 | | cyproheptadine$.mp. | |
| 38 | | desloratadine$.mp. | |
| 39 | | dexbrompheniramine$.mp. | |
| 40 | | dexchlorpheniramine$.mp. | |
| 41 | | dimenhydrinate$.mp. | |
| 42 | | dimethindene$.mp. | |
| 43 | | diphenhydramine$.mp. | |
| 44 | | doxylamine$.mp. | |
| 45 | | ebastine$.mp. | |
| 46 | | epinastine$.mp. | |
| 47 | | fexofenadine$.mp. | |
| 48 | | hydroxyzine$.mp. | |
| 49 | | loratadine$.mp. | |
| 50 | | meclizine$.mp. | |
| 51 | | mequitazine$.mp. | |
| 52 | | methapyrilene$.mp. | |
| 53 | | mizolastine$.mp. | |
| 54 | | oxatomide$.mp. | |
| 55 | | oxomemazine$.mp. | |
| 56 | | pheniramine$.mp. | |
| 57 | | promethazine$.mp. | |
| 58 | | pyrilamine$.mp. | |
| 59 | | quifenadine$.mp. | |
| 60 | | rupatadine$.mp. | |
| 61 | | terfenadine$.mp. | |
| 62 | | trimeprazine$.mp. | |
| 63 | | tripelennamine$.mp. | |
| 64 | | triprolidine$.mp. | |
| 65 | | exp Olopatadine Hydrochloride/ | |
| 66 | | exp Fluticasone/ | |
| 67 | | exp Mometasone Furoate/ | |
| 68 | | exp Beclomethasone/ | |
| 69 | | exp Budesonide/ | |
| 70 | | exp Triamcinolone/ | |
| 71 | | exp Astemizole/ | |
| 72 | | exp Cetirizine/ | |
| 73 | | exp Chlorpheniramine/ | |
| 74 | | exp Cyproheptadine/ | |
| 75 | | exp Clemastine/ | |
| 76 | | exp Hydroxyzine/ | |
| 77 | | exp Loratadine/ | |
| 78 | | exp Ketotifen/ | |
| 79 | | exp Promethazine/ | |
| 80 | | exp Terfenadine/ | |
| 81 | | exp Antazoline/ | |
| 82 | | exp Methapyrilene/ | |
| 83 | | exp Pyrilamine/ | |
| 84 | | exp Tripelennamine/ | |
| 85 | | exp Dimenhydrinate/ | |
| 86 | | exp Diphenhydramine/ | |
| 87 | | exp Doxylamine/ | |
| 88 | | exp Brompheniramine/ | |
| 89 | | exp Dimethindene/ | |
| 90 | | exp Pheniramine/ | |
| 91 | | exp Triprolidine/ | |
| 92 | | exp Meclizine/ or exp Trimeprazine/ | |
| 93 | | exp Rhinitis/ | |
| 94 | | (rhinit$ or rhinoconjunctivit$).mp. | |
| 95 | | exp Allergic Rhinitis/ | |
| 96 | | (hayfever or "hay fever" or pollenosis or pollinosis or SAR).mp. | |
| 97 | | or/1-92 | |
| 98 | | or/93-96 | |
| 99 | | 97 and 98 | |
| 100 | | exp randomized controlled trial/ | |
| 101 | | controlled clinical trial.pt. | |
| 102 | | randomized.ab. | |
| 103 | | placebo.ab. | |
| 104 | | clinical trials as topic/ | |
| 105 | | randomly.ab. | |
| 106 | | trial.ti. | |
| 107 | | or/100-106 | |
| 108 | | exp animals/ not humans/ | |
| 109 | | 107 not 108 | |
| 110 | | 99 and 109 | |
| **CENTRAL (CRS Web)** | | |  |
| 1 | (azelastine* OR azelastine-fluticasone OR azeflu OR mp-azeflu OR (azelastine AND fluticasone) OR dymista OR azecort OR olopatadin* OR olopatadine-mometasone OR (olopatadine AND mometasone) OR gsp301 OR ryaltris OR levocabastin* OR lexicomp OR ciclesonid* OR alvesco OR fluticason* OR gw685698x OR mometason* OR beclometason* OR budesonid* OR triamcinolon* OR antazoline* or methapyrilene* OR pyrilamine* OR tripelennamine* OR clemastine* OR dimenhydrinate* OR diphenhydramine* OR doxylamine* OR brompheniramine* OR chlorpheniramine* OR dimethindene* OR pheniramine* OR triprolidine* OR promethazine* OR cetrizine* OR meclizine* OR hydroxyzine* OR astemizole* OR cyproheptadine* OR loratadine* OR terfenadine* OR acrivastine* OR fexofenadine* OR dexbrompheniramine* OR carbinoxamine* OR bilastine* OR cetirizine* OR desloratadine* OR dexchlorpheniramine* OR dimetindene* OR diphenhydramine* OR ebastine* OR epinastine* OR levocabastine* OR rupatadine* OR mizolastine* OR mequitazine* OR alimemazine* OR trimeprazine* OR oxatomide* OR oxomemazine* OR quifenadine*):ti,ab,kw | |  |
| 2 | MeSH descriptor: [Azelastine] explode all trees | |  |
| 3 | MeSH descriptor: [Olopatadine Hydrochloride] explode all trees | |  |
| 4 | MeSH descriptor: [Fluticasone] explode all trees | |  |
| 5 | MeSH descriptor: [Mometasone Furoate] explode all trees | |  |
| 6 | MeSH descriptor: [Beclomethasone] explode all trees | |  |
| 7 | MeSH descriptor: [Budesonide] explode all trees | |  |
| 8 | MeSH descriptor: [Triamcinolone] explode all trees | |  |
| 9 | MeSH descriptor: [Astemizole] explode all trees | |  |
| 10 | MeSH descriptor: [Cetirizine] explode all trees | |  |
| 11 | MeSH descriptor: [Chlorpheniramine] explode all trees | |  |
| 12 | MeSH descriptor: [Cyproheptadine] explode all trees | |  |
| 13 | MeSH descriptor: [Clemastine] explode all trees | |  |
| 14 | MeSH descriptor: [Hydroxyzine] explode all trees | |  |
| 15 | MeSH descriptor: [Loratadine] explode all trees | |  |
| 16 | MeSH descriptor: [Ketotifen] explode all trees | |  |
| 17 | MeSH descriptor: [Promethazine] explode all trees | |  |
| 18 | MeSH descriptor: [Terfenadine] explode all trees | |  |
| 19 | MeSH descriptor: [Antazoline] explode all trees | |  |
| 20 | MeSH descriptor: [Methapyrilene] explode all trees | |  |
| 21 | MeSH descriptor: [Pyrilamine] explode all trees | |  |
| 22 | MeSH descriptor: [Tripelennamine] explode all trees | |  |
| 23 | MeSH descriptor: [Dimenhydrinate] explode all trees | |  |
| 24 | MeSH descriptor: [Diphenhydramine] explode all trees | |  |
| 25 | MeSH descriptor: [Doxylamine] explode all trees | |  |
| 26 | MeSH descriptor: [Brompheniramine] explode all trees | |  |
| 27 | MeSH descriptor: [Dimethindene] explode all trees | |  |
| 28 | MeSH descriptor: [Pheniramine] explode all trees | |  |
| 29 | MeSH descriptor: [Triprolidine] explode all trees | |  |
| 30 | MeSH descriptor: [Meclizine] explode all trees | |  |
| 31 | MeSH descriptor: [Trimeprazine] explode all trees | |  |
| 32 | MeSH descriptor: [Histamine H1 Antagonists] explode all trees | |  |
| 33 | #1 OR #2 OR #3 OR #4 OR #5 OR #6 OR #7 OR #8 OR #9 OR #10 OR #11 OR #12 OR #13 OR #14 OR #15 OR #16 OR #17 OR #18 OR #19 OR #20 OR #21 OR #22 OR #23 OR #24 OR #25 OR #26 OR #27 OR #28 OR #29 OR #30 OR #31 OR #32 | |  |
| 34 | (rhinit*):ti, ab, kw OR (rhinoconjunctivit*):ti, ab, kw | |  |
| 35 | (hayfever):ti, ab, kw OR ("hay fever"):ti, ab, kw OR (pollenosis):ti, ab, kw OR (pollinosis):ti, ab, kw OR ("SAR"):ti, ab, kw | |  |
| 36 | MESH DESCRIPTOR: [Rhinitis] EXPLODE ALL | |  |
| 37 | MESH DESCRIPTOR: [Rhinitis, Allergic] EXPLODE ALL | |  |
| 38 | #34 OR # 35 OR #36 OR #37 | |  |
| 39 | #33 AND #38 | |  |
| **Embase** | | |  |
| 1 | (azelastin* or 'azelastine-fluticasone' or azeflu or 'MP-azeflu' or (azelastine NEAR/2 fluticasone) or dymista or azecort or olopatadin* or 'olopatadine-mometasone' or (olopatadine NEAR/2 mometasone) or 'gsp301 orryaltris' or levocabastin* or lexicomp or ciclesonid* or Alvesco or fluticason* or GW685698X or mometason* or beclometason* or budesonid* or triamcinolon* or antazoline* or methapyrilene* or pyrilamine* or tripelennamine* or clemastine* or dimenhydrinate* or diphenhydramine* or doxylamine* or brompheniramine* or chlorpheniramine* or dimethindene* or pheniramine* or triprolidine* or promethazine* or cetrizine* or meclizine* or hydroxyzine* or astemizole* or cyproheptadine* or loratadine* or terfenadine* or acrivastine* or fexofenadine* or dexbrompheniramine* or carbinoxamine* or bilastine* or cetirizine* or desloratadine* or dexchlorpheniramine* or dimetindene* or diphenhydramine* or ebastine* or epinastine* or levocabastine* or rupatadine* or mizolastine* or mequitazine* or alimemazine* or trimeprazine* or oxatomide* or oxomemazine* or quifenadine*):ti,ab,tn | |  |
| 2 | (rhinit* or rhinoconjunctivit* or hayfever or "hay fever" or pollenosis or pollinosis):ti,ab,tn | |  |
| 3 | #1 and #2 | |  |
| 4 | 'randomised controlled trial'/exp | |  |
| 5 | 'controlled clinical trial'/exp | |  |
| 6 | random*:ti,ab | |  |
| 7 | 'randomization'/exp | |  |
| 8 | 'intermethod comparison'/exp | |  |
| 9 | placebo:ti,ab | |  |
| 10 | (compare or compared or comparison):ti | |  |
| 11 | ((evaluated or evaluate or evaluating or assessed or assess) and (compare or compared or comparing or comparison)):ab | |  |
| 12 | (open NEAR/1 label):ti,ab | |  |
| 13 | ((double or single or doubly or singly) NEAR/1 (blind or blinded or blindly)):ti,ab | |  |
| 14 | 'double blind procedure'/exp | |  |
| 15 | 'parallel group$':ti,ab | |  |
| 16 | (crossover or 'cross over'):ti,ab | |  |
| 17 | ((assign* or match or matched or allocation) NEAR/5 (alternate or group$ or intervention$ or patient$ or subject$ or participant$)):ti,ab | |  |
| 18 | (assigned or allocated):ti,ab | |  |
| 19 | (controlled NEAR/7 (study or design or trial)):ti,ab | |  |
| 20 | (volunteer or volunteers):ti,ab | |  |
| 21 | 'human experiment'/exp | |  |
| 22 | trial:ti | |  |
| 23 | #4 or #5 or #6 or #7 or #8 or #9 or #10 or #11 or #12 or #13 or #14 or #15 or #16 or #17 or #18 or #19 or #20 or #21 or #22 | |  |
| 24 | (((case NEAR/1 control*) and random*) not 'randomi?ed controlled'):ti,ab | |  |
| 25 | ('systematic review' not (trial or study)):ti | |  |
| 26 | (nonrandom* not random*):ti,ab | |  |
| 27 | "Random field*":ti,ab | |  |
| 28 | ('random cluster' NEAR/3 sampl*):ti,ab | |  |
| 29 | (review:ab and reviewit) not trial:ti | |  |
| 30 | "we searched":ab and (review:ti or review:it) | |  |
| 31 | "update review":ab | |  |
| 32 | (databases NEAR/4 searched):ab | |  |
| 33 | (rat or rats or mouse or mice or swine or porcine or murine or sheep or lambs or pigs or piglets or rabbit or rabbits or cat or cats or dog or dogs or cattle or bovine or monkey or monkeys or trout or marmoset$):ti and 'animal experiment'/exp | |  |
| 34 | 'Animal experiment'/exp not ('human experiment'/exp or 'human'/exp) | |  |
| 35 | #24 or #25 or #26 or #27 or #28 or #29 or #30 or #31 or #32 or #33 or #34 | |  |
| 36 | #23 not #35 | |  |
| 37 | #3 and #36 | |  |
| **Web of Science** | | |  |
| 1 | TS=(azelastin* OR azelastine-fluticasone OR azeflu OR MP-azeflu OR (azelastine NEAR/2 fluticasone) OR dymista OR azecort OR olopatadin* OR olopatadine-mometasone OR (olopatadine NEAR/2 mometasone) OR gsp301 OR ryaltris OR levocabastin* OR lexicomp OR ciclesonid* OR Alvesco OR fluticason* OR GW685698X OR mometason* OR beclometason* OR budesonid* OR triamcinolon* OR antazoline* or methapyrilene* OR pyrilamine* OR tripelennamine* OR clemastine* OR dimenhydrinate* OR diphenhydramine* OR doxylamine* OR brompheniramine* OR chlorpheniramine* OR dimethindene* OR pheniramine* OR triprolidine* OR promethazine* OR cetrizine* OR meclizine* OR hydroxyzine* OR astemizole* OR cyproheptadine* OR loratadine* OR terfenadine* OR acrivastine* OR fexofenadine* OR dexbrompheniramine* OR carbinoxamine* OR bilastine* OR cetirizine* OR desloratadine* OR dexchlorpheniramine* OR dimetindene* OR diphenhydramine* OR ebastine* OR epinastine* OR levocabastine* OR rupatadine* OR mizolastine* OR mequitazine* OR alimemazine* OR trimeprazine* OR oxatomide* OR oxomemazine* OR quifenadine*) | |  |
| 2 | TS=(rhinit* or rhinoconjunctivit*) OR TS=(hayfever or "hay fever" or pollenosis or pollinosis or SAR) | |  |
| 3 | TS=(randomised OR randomised OR randomisation OR randomisation OR placebo* OR (random* AND (allocat* OR assign*)) OR (blind* AND (single OR double OR treble OR triple))) | |  |
| 4 | TS=(animal or animals or pisces or fish or fishes or catfish or catfishes or sheatfish or silurus or arius or heteropneustes or clarias or gariepinus or fathead minnow or fathead minnows or pimephales or promelas or cichlidae or trout or trouts or char or chars or salvelinus or salmo or oncorhynchus or guppy or guppies or millionfish or poecilia or goldfish or goldfishes or carassius or auratus or mullet or mullets or mugil or curema or shark or sharks or cod or cods or gadus or morhua or carp or carps or cyprinus or carpio or killifish or eel or eels or anguilla or zander or sander or lucioperca or stizostedion or turbot or turbots or psetta or flatfish or flatfishes or plaice or pleuronectes or platessa or tilapia or tilapias or oreochromis or sarotherodon or common sole or dover sole or solea or zebrafish or zebrafishes or danio or rerio or seabass or dicentrarchus or labrax or morone or lamprey or lampreys or petromyzon or pumpkinseed or pumpkinseeds or lepomis or gibbosus or herring or clupea or harengus or amphibia or amphibian or amphibians or anura or salientia or frog or frogs or rana or toad or toads or bufo or xenopus or laevis or bombina or epidalea or calamita or salamander or salamanders or newt or newts or triturus or reptilia or reptile or reptiles or bearded dragon or pogona or vitticeps or iguana or iguanas or lizard or lizards or anguis fragilis or turtle or turtles or snakes or snake or aves or bird or birds or quail or quails or coturnix or bobwhite or colinus or virginianus or poultry or poultries or fowl or fowls or chicken or chickens or gallus or zebra finch or taeniopygia or guttata or canary or canaries or serinus or canaria or parakeet or parakeets or grasskeet or parrot or parrots or psittacine or psittacines or shelduck or tadorna or goose or geese or branta or leucopsis or woodlark or lullula or flycatcher or ficedula or hypoleuca or dove or doves or geopelia or cuneata or duck or ducks or greylag or graylag or anser or harrier or circus pygargus or red knot or great knot or calidris or canutus or godwit or limosa or lapponica or meleagris or gallopavo or jackdaw or corvus or monedula or ruff or philomachus or pugnax or lapwing or peewit or plover or vanellus or swan or cygnus or columbianus or bewickii or gull or chroicocephalus or ridibundus or albifrons or great tit or parus or aythya or fuligula or streptopelia or risoria or spoonbill or platalea or leucorodia or blackbird or turdus or merula or blue tit or cyanistes or pigeon or pigeons or columba or pintail or anas or starling or sturnus or owl or athene noctua or pochard or ferina or cockatiel or nymphicus or hollandicus or skylark or alauda or tern or sterna or teal or crecca or oystercatcher or haematopus or ostralegus or shrew or shrews or sorex or araneus or crocidura or russula or european mole or talpa or chiroptera or bat or bats or eptesicus or serotinus or myotis or dasycneme or daubentonii or pipistrelle or pipistrellus or cat or cats or felis or catus or feline or dog or dogs or canis or canine or canines or otter or otters or lutra or badger or badgers or meles or fitchew or fitch or foumart or foulmart or ferrets or ferret or polecat or polecats or mustela or putorius or weasel or weasels or fox or foxes or vulpes or common seal or phoca or vitulina or grey seal or halichoerus or horse or horses or equus or equine or equidae or donkey or donkeys or mule or mules or pig or pigs or swine or swines or hog or hogs or boar or boars or porcine or piglet or piglets or sus or scrofa or llama or llamas or lama or glama or deer or deers or cervus or elaphus or cow or cows or bos taurus or bos indicus or bovine or bull or bulls or cattle or bison or bisons or sheep or sheeps or ovis aries or ovine or lamb or lambs or mouflon or mouflons or goat or goats or capra or caprine or chamois or rupicapra or leporidae or lagomorpha or lagomorph or rabbit or rabbits or oryctolagus or cuniculus or laprine or hares or lepus or rodentia or rodent or rodents or murinae or mouse or mice or mus or musculus or murine or woodmouse or apodemus or rat or rats or rattus or norvegicus or guinea pig or guinea pigs or cavia or porcellus or hamster or hamsters or mesocricetus or cricetulus or cricetus or gerbil or gerbils or jird or jirds or meriones or unguiculatus or jerboa or jerboas or jaculus or chinchilla or chinchillas or beaver or beavers or castor fiber or castor canadensis or sciuridae or squirrel or squirrels or sciurus or chipmunk or chipmunks or marmot or marmots or marmota or suslik or susliks or spermophilus or cynomys or cottonrat or cottonrats or sigmodon or vole or voles or microtus or myodes or glareolus or primate or primates or prosimian or prosimians or lemur or lemurs or lemuridae or loris or bush baby or bush babies or bushbaby or bushbabies or galago or galagos or anthropoidea or anthropoids or simian or simians or monkey or monkeys or marmoset or marmosets or callithrix or cebuella or tamarin or tamarins or saguinus or leontopithecus or squirrel monkey or squirrel monkeys or saimiri or night monkey or night monkeys or owl monkey or owl monkeys or douroucoulis or aotus or spider monkey or spider monkeys or ateles or baboon or baboons or papio or rhesus monkey or macaque or macaca or mulatta or cynomolgus or fascicularis or green monkey or green monkeys or chlorocebus or vervet or vervets or pygerythrus or hominoidea or ape or apes or hylobatidae or gibbon or gibbons or siamang or siamangs or nomascus or symphalangus or hominidae or orangutan or orangutans or pongo or chimpanzee or chimpanzees or pan troglodytes or bonobo or bonobos or pan paniscus or gorilla or gorillas or troglodytes) | |  |
| 5 | #3 NOT #4 | |  |
| 6 | #1 AND #2 AND #5 | |  |

**Supplementary Table 2. Decision thresholds adopted to evaluate the certainty of the evidence for the different outcomes ^a^**

| Outcome | Decision thresholds  [reference] |
| --- | --- |
| Total Nasal Symptom Score; Total Ocular Symptoms Score; Rhinoconjunctivitis Quality of Life Questionnaire (standardised mean differences) | -0.8/-0.5/-0.2/  0.2/0.5/0.8 |
| Occurrence of any adverse event and withdrawal due to adverse events: Intranasal antihistamines | -862/-442/-187/  187/442/862 ^a^ |
| Occurrence of any adverse event and withdrawal due to adverse events: Intranasal corticosteroids | -637/-327/-138/  138/327/637 ^a, b^ |
| Occurrence of any adverse event and withdrawal due to adverse events: Oral medications | -303/-161/-65/  65/161/303 ^a, c^ |

^a^ Per 1000 persons; ^b^ Reference: Sousa-Pinto B, Bognanni A, Gil-Mata S, et al. Empirical estimation of disutilities and decision thresholds for composite endpoints. J Clin Epidemiol. 2025;179:111638; ^c^ Reference: Vieira RJ, Gil-Mata S, Ferreira A, et al. Efficacy and safety of oral antihistamines for allergic rhinitis: Network meta-analysis. J Allergy Clin Immunol Pract. 2026:S2213-2198(26)00140-6.

**Supplementary Table 3. Standard daily doses in adult patients that were considered for the different individual medications ^a^**

| Medication | Standard daily dose of active compounds |
| --- | --- |
| Azelastine (intranasal) | 560 μg |
| Azelastine-fluticasone (intranasal) | 748 μg ^b^ |
| Beclomethasone (intranasal) | 320 μg |
| Bilastine (oral) | 20 mg |
| Budesonide (intranasal) | 256 μg |
| Cetirizine (oral) | 10 mg |
| Ciclesonide (intranasal) | 200 μg |
| Desloratadine (oral) | 5 mg |
| Ebastine (oral) | 10 mg |
| Fexofenadine (oral) | 180 mg |
| Fluticasone furoate (intranasal) | 110 μg |
| Fluticasone propionate (intranasal) | 200 μg |
| Levocetirizine (oral) | 5 mg |
| Loratadine (oral) | 10 mg |
| Mometasone (intranasal) | 200 μg |
| Montelukast (oral) | 10 mg |
| Olopatadine (intranasal) | 5320 μg |
| Olopatadine-mometasone (intranasal) | 5520 μg ^c^ |
| Rupatadine (oral) | 10 mg |
| Terfenadine (oral) | 120 mg |
| Triamcinolone (intranasal) | 220 μg |

^a^ Doses defined based on the literature (i.e., doses most commonly evaluated in randomized controlled trials) and after discussion with allergists. In some cases, we defined the standard dose ad the lower end of a recommended or approved dose range; ^b^ 548 μg of azelastine + 200 μg of fluticasone propionate; ^c^ 5320 μg of olopatadine + 200 μg of mometasone.

**Supplementary Table 4. Description of the primary studies included in the systematic review**

| **Study identification** | **Place and period of data collection** | **Active treatment** | **Assessed outcomes** | **Follow-up** | ***N* participants** | ***N* (%) completers** | ***N* (%) females** | **Mean age (years)** |
| --- | --- | --- | --- | --- | --- | --- | --- | --- |
| **Adults with perennial allergic rhinitis** | | | | | | | | |
| Spector S, 1989 ^21^ | US; Winter and Spring seasons | Triamcinolone nasal aerosol 200 μg (1 spray/nostril QID)  Placebo | - rTNSS | 12 weeks | 205 | 152 (74.1) | 104 (50.7) | 36.8 |
| Day J, 1990 ^22^ | - | Budesonide nasal spray 400 μg (2 sprays/nostril BID)  Placebo | - rTNSS | 4 weeks | 48 | - | 28 (58.3) | 43.9 |
| Storms W, 1991^23^ | US | Triamcinolone nasal aerosol 110 μg (1 spray/nostril QD)  Triamcinolone nasal aerosol 220 μg (1 spray/nostril QD)  Triamcinolone nasal aerosol 440 μg (1 spray/nostril QD)  Placebo | - rTNSS | 12 weeks | 305 | 262 (85.9) | 100 (32.8) | 25.5 |
| van As A, 1993 ^24^  (FNM30006) | US; 1998 (December) – 1989 (October) | Beclomethasone nasal spray 336 μg (2 sprays/ nostril BID)  Fluticasone propionate 200 μg (2 sprays/nostril BID)  Fluticasone propionate 200 μg (2 sprays/nostril QD)  Placebo | - rTNSS | 26 weeks | 466 | 360 (77.3) | 239 (51.3) | 36.3 |
| Banov CH, 1994 ^25^  (FNM30007) | US; 1988 December – 1989 (October) | Fluticasone propionate 200 μg (BID)  Fluticasone propionate 200 μg (QD)  Placebo | - rTNSS | 26 weeks | 365 | 299 (81.9) | 181 (49.6) | 35.5 |
| Kobayashi RH, 1995 ^26^ | US | Triamcinolone nasal spray 220 μg (2 sprays/nostril QD)  Placebo | - rTNSS | 4 weeks | 178 | 172 (96.6) | 68 (38.2) | 31 |
| Drouin M, 1996 ^27^ | Europe and Canada | Beclomethasone nasal spray 400 μg (2 sprays/ nostril BID)  Mometasone 200 μg (2 sprays/nostril QD)  Placebo | - rTNSS | 12 weeks | 427 | 327 (76.6) | 194 (45.4) | 31.7 |
| Mandl M, 1997 ^28^ | Europe, Latin America and Canada | Fluticasone propionate 200 μg (2 sprays/nostril QD)  Mometasone 200 μg (2 sprays/nostril QD)  Placebo | - rTNSS | 12 weeks | 548 | 474 (86.5) | 300 (54.7) | 33.0 |
| Bachert C, 1998 ^29^ | Germany and UK (22 sites); - | Mizolastine 10 mg (QD)  Placebo | - rTNSS  - rTOSS | 4 weeks | 257 | 220 (85.6) | 155 (60.3) | 35 |
| Davies RJ, 1998 ^30^ | Europe (45 sites); 1995 (November) - 1996 (April) | Ebastine 10 mg (QD)  Ebastine 20 mg (QD)  Loratadine 10 mg (QD) | - rTNSS | 4 weeks | 317 | 301 (95.0) | 156 (49.2) | 31.9 |
| Day J, 1998 ^31^ | Canada and Spain; 1994 (November) – 1995 (July) | Budesonide nasal spray 256 μg (2 sprays/nostril QD)  Fluticasone propionate 200 μg (2 sprays/nostril QD)  Placebo | - rTNSS | 6 weeks | 273 | - | 150  (54.9) | 30.8 |
| Meltzer EO, 1998 ^32^ | US; 1994 (Autumn pollen season) | Budesonide nasal spray 32 μg (QD)  Budesonide nasal spray 64 μg (QD)  Budesonide nasal spray 128 μg (QD)  Budesonide nasal spray 256 μg (QD)  Placebo | - rTNSS  - RQLQ | 6 weeks | 478 | 447 (93.5) | 233 (48.7) | 24.7 |
| Murris-Espin M, 1998 ^33^ | France (18 sites); 1994 (August) - 1995 (April) | Cetirizine 10 mg (QD)  Ebastine 10 mg (QD) | - rTNSS | 4 weeks | 214 | - | 120 (56.1) | 31.2 |
| Stern MA, 1998 ^34^ | United Kingdom | Azelastine 0.10% (1 spray/nostril BID)  Budesonide nasal spray 256 μg (QD)  Placebo | - rTNSS | 6 weeks | 195 | 145 (74.4) | 96 (49.2) | 36.9 |
| Bousquet J, 1999 ^35^ | France, Portugal, Spain (37 sites); October-May | Ebastine 10 mg (QD)  Ebastine 20 mg (QD)  Placebo | - rTNSS | 12 weeks | 290 | 248 (85.5) | 147 (50.7) | 25.6 |
| Holm AF, 1999 ^36^  (FNM30029) | Netherlands; 1988 (September) – 1990 (April) | Fluticasone propionate 200 μg (2 sprays/nostril QD)  Placebo | - rTNSS | 52 weeks | 42 | 29 (69.0) | 14 (33.3) | 28.6 |
| Kivisaari E, 2001 ^37^ | Austria, Finland, Indonesia, New Zealand, Taiwan, and the Netherlands | Budesonide 200 μg  Fluticasone propionate 200 μg  Placebo | - rTNSS | 8 weeks | 333 | 302 (90.7) | 196 (58.9) | 28.6 |
| Weckx LLM, 2001 ^38^ | Brazil; - | Triamcinolone nasal spray 220 μg (2 sprays/ nostril QD)  Beclomethasone nasal spray 400 μg (2 sprays/nostril BID) | - rTNSS | 4 weeks | 162 | 141 (87.0) | 86 (61.0) | 26.2 |
| Bende M, 2002 ^39^ | Hungary, Portugal, Spain and Sweden | Budesonide nasal spray 128 μg (QD)  Budesonide nasal spray 256 μg (QD)  Mometasone 200 μg  Placebo | - rTNSS | 4 weeks | 437 | 400 (91.5) | 252 (57.7) | 31.0 |
| Frèche C, 2002 ^40^ | Europe (41 sites); 1994 (October) - 1996 (February) | Mizolastine 10 mg (QD)  Loratadine 10 mg (QD)  Placebo | - rTNSS | 4 weeks | 428 | 394 (92.1) | 230 (53.7) | 32 |
| R1810220 ^41^ | US; 2002 (January-April) | Fluticasone propionate 100 μg (QD)  Placebo | - rTNSS | 4 weeks | 361 | 327 (90.6) | 226 (62.6) | 35.3 |
| Simons F, 2003 ^42^ | USA (33 sites); - | Desloratadine 5 mg (QD)  Placebo | - rTNSS | 4 weeks | 676 | 634 (93.8) | 477 (70.6) | 34.8 |
| Bachert C, 2004 ^43^ | Europe (63 sites); - | Levocetirizine 5 mg (QD)  Placebo | - RQLQ | 24 weeks | 551 | 420 (76.2) | 310 (56.3) | 30.3 |
| Aguiar FAB, 2006 ^44^ | Brazil | Mometasone 200 μg (2 sprays/nostril BID)  Triamcinolone nasal spray 220 μg (1 spray/nostril BID)  Placebo | - rTNSS | 12 weeks | 61 | - | 27 (44.3) | 28.6 |
| Chervinsky P, 2007 ^45^  (NCT00163501) | US; 2004 (January) – 2005 (April) | Ciclesonide nasal spray 200 μg (2 sprays/nostril QD)  Placebo | - rTNSS  - RQLQ | 48 weeks | 663 | 474 (71.5) | 436 (65.8) | 37.0 |
| Meltzer EO, 2007 ^46^  (NCT00659750) | US and Canada; 2003 (December) – 2004 (December) | Ciclesonide nasal spray 200 μg (2 sprays/nostril QD)  Placebo | - rTNSS  - RQLQ | 6 weeks | 471 | 409 (86.8) | 305 (64.8) | 35.5 |
| Philip G, 2007 ^47^  (NCT00974571) | USA (74 sites); 2001 (November) - 2002 (May) | Cetirizine 10 mg (QD)  Montelukast 10 mg (QD)  Placebo | - rTNSS  - RQLQ | 6 weeks | 1355 | 1198 (88.4) | 922 (68.0) | 35.4 |
| Rosenblut A, 2007 ^48^  (FFR102123) | Australia, Chile, Estonia, Germany, Italy, Latvia, Lithuania, Netherlands, New Zealand, Romania, Russia, Spain, Sweden; 2004 September – 2005 December | Fluticasone furoate 110 μg (2 sprays/ nostril QD)  Placebo | - rTNSS | 52 weeks | 806 | 592 (73.4) | 413 (51.2) | 32.4 |
| Fantin S, 2008 ^49^ | Argentina, Chile, Romania (33 sites); 2004 (September) - 2005 (May) | Rupatadine 10 mg (QD)  Cetirizine 10 mg (QD)  Placebo | - RQLQ | 12 weeks | 543 | 479 (88.2) | 354 (65.2) | 29.3 |
| Nathan RA, 2008 ^50^  (NCT00103454) | US and Canada; 2005 | Fluticasone furoate 110 μg (2 sprays/ nostril QD)  Placebo | - rTNSS  - rTOSS  - RQLQ | 4 weeks | 302 | 279 (92.4) | 189 (62.6) | 36.7 |
| Patel D, 2008 ^51^  (NCT00116818) | US and Canada; 2005 | Fluticasone furoate 110 μg (2 sprays/ nostril QD)  Placebo | - rTNSS | 6 weeks | 99 | 88 (88.9) | 52 (52.5) | 36.5 |
| Vasar M, 2008 ^52^ (NCT00289198) | Australia, Canada, Estonia, Germany, Latvia, Lithuania, New Zealand, Russia, US; 2006 | Fluticasone furoate 110 μg (2 sprays/ nostril QD)  Placebo | - rTNSS  - rTOSS  - RQLQ | 6 weeks | 302 | 241 (79.8) | 171 (56.6) | 37.2 |
| Holmberg K, 2009 ^53^ | France and Sweden (115 sites); October-March | Desloratadine 5 mg (QD)  Placebo | - rTNSS  - RQLQ | 4 weeks | 584 | - | 305 (52.2) | 34.4 |
| Kowalski ML, 2009 ^54^ | Poland and Czech Republic (22 sites); October-March | Rupatadine 10 mg (QD)  Rupatadine 20 mg (QD)  Loratadine 10 mg (QD)  Placebo | - rTNSS | 4 weeks | 283 | 265 (93.6) | 149 (52.7) | 29.4 |
| Bernstein J, 2010 ^55^  (NCT00712920) | US; 2006-2007 Winter allergy season | Azelastine 0.10% (2 sprays/nostril BID)  Azelastine 0.15% (2 sprays/nostril BID)  Placebo | - rTNSS  - RQLQ | 4 weeks | 578 | 535 (92.6) | 393 (68.0) | 36.9 |
| Bousquet J, 2010 ^56^ | Europe and North America (83 sites); 2006 (September) - 2007 (November) | Desloratadine 5 mg (QD)  Placebo | - RQLQ | 12 weeks | 716 | 562 (78.5) | 406 (56.7) | 33.9 |
| Given JT, 2010 ^57^  (NCT00609674) | Canada, Estonia, Germany, Hungary, Russia, Slovakia, US; 2008 (January-June) | Fluticasone furoate 110 μg (QD)  Placebo | - rTNSS  - rTOSS  - RQLQ | 4 weeks | 315 | 301 (95.6) | 213 (67.6) | 38.7 |
| Grossman J, 2010 ^58^ | US | Mometasone 200 μg (QD)  Placebo | - rTNSS | 12 weeks | 334 | 285 (85.3) | - | 70.5 |
| Meltzer EO, 2010 ^59^ (NCT00359216) | US | Mometasone 200 μg (2 sprays/nostril QD)  Placebo | - rTNSS  - RQLQ | 4 weeks | 30 | 29 (96.7) | 16 (53.3) | 34.3 |
| Molina M, 2010 ^60^ | Spain (29 sites); 1999-2000 | Rupatadine 10 mg (QD)  Ebastine 10 mg (QD)  Placebo | - rTNSS | 4 weeks | 223 | 201 (90.1) | 115 (51.6) | 27.7 |
| NCT01033825 ^61^ | US; 2010 (January – May) | Ciclesonide nasal spray 200 μg (QD)  Ciclesonide nasal aerosol 160 μg (QD)  Ciclesonide nasal aerosol 320 μg (QD)  Placebo | - rTNSS | 6 weeks | 274 | 262 (95.6) | 169 (61.7) | 34.3 |
| Baroody FM, 2011 ^62^  (NCT00584987) | US; 2007 November – 2009 May | Fluticasone furoate 110 μg (2 sprays/ nostril QD)  Oxymetazoline 0.05% (2 sprays/nostril QD)  Fluticasone furoate 110 μg (2 sprays/ nostril QD) + oxymetazoline 0.05% (2 sprays/nostril QD)  Placebo | - RQLQ | 4 weeks | 64 | 60 (93.8) | 41 (64.1) | 28.8 |
| Marmouz F, 2011 ^63^ | France (multiple sites); - | Rupatadine 10 mg (QD)  Rupatadine 20 mg (QD)  Cetirizine 10 mg (QD)  Placebo | - rTNSS | 4 weeks | 282 | 269 (95.4) | 170 (60.3) | 32.1 |
| Berger WE, 2012 ^64^  (NCT00953147) | US; 2009 (August) – 2010 (May) | Ciclesonide nasal aerosol 80 μg (1 spray/nostril QD)  Ciclesonide nasal aerosol 160 μg (1 spray/nostril QD)  Placebo | - rTNSS  - RQLQ | 26 weeks | 1110 | 965 (86.9) | 716 (64.5) | 37.2 |
| Fokkens WJ, 2012 ^65^ (NCT00224523) | Netherlands, Belgium; September 2005-February 2007 | Mometasone furoate 200 μg (2 sprays/nostril QD)  Fluticasone furoate 110 μg (2 sprays/ nostril QD) | - rTNSS | 52 weeks | 116 | 96 (82.8) | 78 (67.2) | 31.4 |
| Guilemany JM, 2012 ^66^ | -; 2005 (January) - 2007 (February) | Levocetirizine 5 mg (QD)  Placebo | - RQLQ | 4 weeks | 27 | - | 13 (48.1) | 31.9 |
| Meltzer EO, 2012 ^67^  (NCT01134705) | US; 2010 (May-October) | Beclomethasone aerosol 320 μg (2 sprays/nostril QD)  Placebo | - rTNSS  - RQLQ | 6 weeks | 474 | 437 (92.9) | 319 (67.3) | 37.0 |
| Aneeza WH, 2013 ^68^ | Malaysia; - | Mometasone furoate 200 μg (QD)  Fluticasone furoate 110 μg (QD) | - rTNSS  - rTOSS  - RQLQ | 8 weeks | 78 | 63 (80.8) | 37 (47.4) | 28.7 |
| EUCTR-2013-000654-22-DE ^69^ | Germany; 2013 (September) – 2015 (February) | Mometasone 200 μg (1 spray/nostril BID)  Placebo | - rTNSS | 2 years | 101 | 92 (91.1) | 66 (65.3) | - |
| LaForce C, 2013 ^70^  (NCT00682643) | US; 2008 June – 2011 February | Fluticasone furoate 110 μg (2 sprays/ nostril QD)  Placebo | - rTNSS | 104 weeks | 550 | 303 (55.1) | 371 (67.5) | 37.4 |
| Goh BS, 2014 ^71^ | Malaysia; 2009 (February-November) | Fluticasone propionate 100 μg (1 spray/nostril QD)  Fluticasone propionate 100 μg (1 spray/nostril QD) + montelukast 10 mg (QD) | - rTNSS | 8 weeks | 128 | - | 60  (46.9) | 24.5 |
| NCT00806754 | US (35 sites); 2006 (November) – 2007 (May) | Ciclesonide 200 μg (1 spray/nostril BID)  Ciclesonide 200 μg (1 spray/nostril BID) + azelastine 1120 μg (2 sprays/nostril BID) | - rTNSS | 4 weeks | 298 | - | - | - |
| Skoner D, 2014 ^72^ | USA (15 sites); - | Cetirizine 10 mg (QD)  Placebo | - RQLQ | 4 weeks | 321 | 298 (92.8) | 225 (70.1) | 37.3 |
| Weinstein SF, 2014 ^73^  (NCT00988247) | US | Beclomethasone aerosol 320 μg (2 sprays/ nostril QD)  Placebo | - rTNSS  - RQLQ | 30-52 weeks | 529 | 420 (79.4) | 352 (66.5) | 37.1 |
| Jia M, 2017 ^74^ | China | Mometasone 200 μg (2 sprays/nostril QD)  Mometasone 200 μg (2 sprays/nostril QD) + loratadine 10 mg (QD)  Mometasone 200 μg (2 sprays/nostril QD) + montelukast 10 mg (QD) | - rTNSS | 4 weeks | 57 | - | 26 (45.6) | 29.7 |
| Lv C, 2018 ^75^ | China | Budesonide nasal spray 4800 μg (2 sprays/nostril TID)  Placebo | - rTNSS | 4 weeks | 100 | - | - | - |
| Karaulov AV, 2019 ^76^  (NCT03317015) | Russia; November 2016 - July 2017 | Triamcinolone nasal spray 220 μg (2 sprays/ nostril QD)  Fluticasone propionate 200 μg (2 sprays/nostril QD | - rTNSS  - RQLQ | 4 weeks | 260 | 256 (98.5) | 160 (61.5) | 32.5 |
| Segall N, 2019 ^77^  (NCT02709538) | US | Olopatadine-mometasone - 665 μg olopatadine + 25 μg mometasone (2 sprays/nostril BID)  Placebo | - rTNSS  - RQLQ | 52 weeks | 593 | 440 (74.2) | 405 (68.3) | 40.8 |
| Mo JH, 2020 ^78^ | South Korea (13 sites); 2017 (January- October) | Bepotastine 20 mg (QD)  Placebo | - rTNSS | 4 weeks | 272 | 229 (84.2) | 132 (48.5) | 34.0 |
| Wagner K, 2020 ^79^ | - | Budesonide nasal spray 128 μg Budesonide nasal spray 256 μg  Placebo | - rTNSS | 6 weeks | 144 | - | - | - |
| Won T, 2021 ^80^ | South Korea (6 sites); 2013 (August) - 2016 (July) | Rupatadine 10 mg (QD)  Bepotastine 20 mg (BID)  Placebo | - rTNSS | 4 weeks | 330 | 306 (92.7) | 183 (55.5) | 29.1 |
| Bousquet J, 2025 ^81^ (NCT00720382) | US, March 2007- June 2008 | Azelastine 0.15% (2 sprays/ nostril BID)  Mometasone furoate 200 μg (2 sprays/ nostril QD) | - RQLQ | 52 weeks | 703 | 468 (66.6) | 485 (69.0) | 39.2 |
| **Adults with seasonal allergic rhinitis** | | | | | | | | |
| Andersen JB, 1975 ^82^ | Denmark; 1974 (grass pollen season) | Beclomethasone aerosol 200 μg (1 spray/nostril BID)  Beclomethasone aerosol 300 μg (1 spray/nostril TID)  Beclomethasone aerosol 400 μg (1 spray/nostril QID)  Placebo | - rTNSS | 2 weeks | 24 | 20 (83.3) | 11 (45.8) | 22.7 |
| Munch EP, 1983^83^ | Denmark; 1982 Grass Pollen Season (June) | Budesonide 400 mcg (2 sprays/nostril BID)  Dexchlorpheniramine maleate 12 mg (BID) | - rTNSS | 3 weeks | 61 | 60 (98.4) | 30 (49.2) | 29 |
| Gutkowski A, 1985 ^84^ | Quebec, Canada (4 sites); 1983 (August - September) | Dexchlorpheniramine 12 mg (BID)  Terfenadine 120 mg (BID) | - rTNSS  - rTOSS | 2 weeks | 174 | 129 (74.1) | - | - |
| Skassa-Brociek W, 1988 ^85^ | - | Loratadine 10 mg (QD)  Mequitazine 10 mg (BID) Placebo | - rTNSS | 2 weeks | 69 | 64 (92.8) | 33 (47.8) | 30.7 |
| Tinkelman D, 1989 ^86^ | - | Triamcinolone nasal aerosol 110 μg (1 spray/nostril QID)  Placebo | - rTNSS | 4 weeks | 180 | 168 (93.3) | 75 (41.7) | 33.0 |
| Meltzer EO, 1990 ^87^  (FLN202) | US; 1988 (April – July) | Fluticasone propionate 50 μg (1 spray/nostril BID)  Fluticasone propionate 200 μg (1 spray/nostril BID)  Fluticasone propionate 800 μg (1 spray/nostril BID)  Placebo | - rTNSS | 2 weeks | 423 | 415 (98.1) | 102 (24.1) | 33.4 |
| Nathan RA, 1991 ^88^  (FLN-203) | US; 1988 (August – November) | Fluticasone propionate 200 μg (2 sprays/nostril QD)  Fluticasone propionate 200 μg (2 sprays/nostril BID)  Placebo | - rTNSS | 2 weeks | 227 | 221 (97.4) | 97 (42.7) | 34.2 |
| van As A, 1991 ^89^ | US | Fluticasone propionate 100 μg (2 sprays/nostril BID)  Placebo | - rTNSS | 4 weeks | 49 | 47 (95.9) | 17 (34.7) | 36.5 |
| FLN-270 ^90^ | 1991 | Fluticasone propionate 200 μg (QD)  Placebo | - rTNSS | 4 weeks | 304 | 300 (98.7) | 126 (41.4) | 32.1 |
| Findlay S, 1992 ^91^ | US | Triamcinolone l10 μg (1 spray/nostril QD)  Triamcinolone 220 μg (1 spray/nostril QD)  Triamcinolone 440 μg (1 spray/nostril QD)  Placebo | - rTNSS | 4 weeks | 304 | 278 (91.4) | 79 (26.0) | 23.3 |
| Norman PS, 1992 ^92^ | -; (grass pollen season) | Budesonide nasal spray 400 μg (2 sprays/nostril BID)  Placebo | - rTNSS | 4 weeks | 50 | 48 (96.0) | 26 (52.0) | 30.3 |
| Ratner PH, 1992 ^93^  (FNM40123) | US; 1988 (December) – 1989 (February) | Beclomethasone nasal spray 336 μg (2 sprays/nostril BID)  Fluticasone propionate 200 μg (2 sprays/nostril QD)  Placebo | - rTNSS | 2 weeks | 313 | 309 (98.7) | 141 (45.0) | 37.1 |
| Dockhorn RJ, 1993 ^94^  (FLN-204) | US; 1988 (August – December) | Fluticasone propionate 200 μg (2 sprays/nostril QD)  Fluticasone propionate 200 μg (2 sprays/nostril BID)  Placebo | - rTNSS | 4 weeks | 301 | 289 (96.0) | 111 (36.9) | 32.3 |
| NCT03855189 ^95^ | 1993 (August-October) | Beclomethasone nasal spray 336 μg (BID)  Mometasone 200 μg (QD)  Placebo | - rTNSS | 4 weeks | 343 | 322 (93.9) | 176 (51.0) | 34.9 |
| Dahl R, 1994 ^96^ | Denmark, Germany, Netherlands | Levocabastine 0.05% (2 sprays/ nostril BID)  Placebo | - rTNSS | 4 weeks | 262 | 210 (80.2) | 146 (55.7) | 31.7 |
| LaForce CF, 1994 ^97^  (FLN-306) | US; 1989 (March-July) | Beclomethasone nasal spray 336 μg (2 sprays/nostril BID)  Fluticasone propionate 200 μg (2 sprays/nostril QD)  Fluticasone propionate 200 μg (2 sprays/nostril BID)  Placebo  Placebo | - rTNSS | 4 weeks | 238 | 235 (98.7) | 69 (29.0) | 23.1 |
| Mackowiak JI, 1994 ^98^  (FLN-412) | United States; 1992 (March - July) | Fluticasone propionate 200 mcg (QD)  Astemizole 10 mg (QD)  Placebo | - rTNSS | 4 weeks | 304 | 272 (89.5) | 139 (45.7) | 32.1 |
| McArthur JG, 1994 ^99^ | UK; 1993 (May -August) | Budesonide nasal spray 400 μg (2 sprays/ nostril BID)  Beclomethasone nasal spray 400 μg (2 sprays/nostril BID) | - rTNSS | 3 weeks | 88 | 66 (75.0) | 45 (51.1) | 27.0 |
| Munk ZM, 1994 ^100^  (FLN-305) | US; 1989 (April – July) | Fluticasone propionate 200 μg (2 sprays/nostril QD)  Fluticasone propionate 200 μg (2 sprays/nostril BID)^a^  Placebo | - rTNSS | 2 weeks | 243 | 237 (97.5) | 17 (7.0) | 14.2 |
| van Bavel J, 1994 ^101^  (FLN-401) | Texas, US; 1990 (December) – 1991 (March) | Fluticasone propionate 200 μg (2 sprays/nostril QD)  Terfenadine 120 mg (BID)  Placebo | - rTNSS | 2 weeks | 232 | 217 (93.5) | 118 (50.9) | 39.7 |
| NCT03882047 ^102^ | 1994 (August-September) | Fluticasone propionate 200 μg (QD)  Mometasone 200 μg (QD)  Placebo | - rTNSS | 2 weeks | 312 | 281 (90.1) | 141 (45.3) | 31.3 |
| Bronsky E, 1995 ^103^ | USA (14 sites); 1989 (Fall) | Loratadine 10 mg (QD)  Placebo | - rTNSS | 2 weeks | 433 | 403 (93.1) | 230 (53.1) | - |
| Pelucchi A, 1995 ^104^ | Italy; 1993 (April-June) | Azelastine 0.10% (1 spray/nostril BID)  Beclomethasone nasal spray 200 μg (1 spray/nostril BID)  Placebo | - rTNSS  - rTOSS | 5 weeks | 36 | 36 (100) | 15 (41.7) | 25.6 |
| Prenner BM, 1995 ^105^ | US; 1993 (Autumn) | Beclomethasone nasal spray 336 μg (2 sprays/nostril QD)  Beclomethasone nasal spray 336 μg (2 sprays/nostril BID)  Beclomethasone nasal spray 1344 μg (2 sprays/nostril QD)  Placebo | - rTNSS | 4 weeks | 438 | 410 (93.6) | 251 (57.3) | - |
| Settipane G, 1995 ^106^ | US; ragweed pollen season | Triamcinolone nasal spray 220 μg (2 sprays/nostril QD)  Placebo | - rTNSS | 3 weeks | 287 | - | 131 (45.6) | 37 |
| Bernstein DI, 1996 ^107^ | United States; Ragweed Pollen Season | Triamcinolone acetonide 220 mcg (2 sprays/nostril QD)  Astemizole 10 mg (QD) | - rTNSS | 4 weeks | 239 | 215 (90) | 128 (53.6) | 35.7 |
| Bronsky EA, 1996 ^108^  (FLN-402) | US; Spring allergy season (February – May) | Fluticasone propionate 200 μg (2 sprays/nostril QD)  Terfenadine 120 mg (BID)  Placebo | - rTNSS | 4 weeks | 348 | 319 (91.7) | 145 (41.7) | 29.5 |
| Cook CK, 1996 ^109^  (FLTA4004) | US | Fluticasone propionate 200 μg (QD)  Loratadine 10 mg (QD)  Placebo | - rTNSS | 4 weeks | 333 | 308 (92.5) | 147 (44.1) | 35.0 |
| Harvey R, 1996 ^110^ | -; Ragweed allergy season | Cetirizine 5-10 mg (-)  Chlorpheniramine 12-16 mg (TID/QID)  Terfenadine 120 mg (BID) | - RQLQ | 2 weeks | 131 | 122 (93.1) | 79 (60.3) | 34.4 |
| Hebert JR, 1996 ^111^ | Canada and Europe; 1993 (Spring and Summer) | Beclomethasone nasal spray 400 μg (2 sprays/nostril BID)  Mometasone 100 μg (2 sprays/nostril QD)  Mometasone 200 μg (2 sprays/nostril QD)  Placebo | - rTNSS | 4 weeks | 498 | 434 (87.1) | 42 (8.4) | 32.2 |
| Howland WC, 1996 ^112,113^ | US | Fluticasone propionate 200 μg (2 sprays/nostril QD)  Placebo | - rTNSS | 2 weeks | 154 | 139 (90.3) | 82 (53.2) | 39.6 |
| Munk ZM, 1996 ^114^ | US; ragweed pollen season | Triamcinolone nasal spray 220 μg (2 sprays/nostril QD)  Placebo | - rTNSS | 2 weeks | 140 | 123 (87.9) | 64 (45.7) | 36 |
| Schoenwetter W, 1996 ^115^ | United States; Ragweed Pollen Season | Triamcinolone acetonide 220 mcg (2 sprays/nostril QD)  Loratadine 10 mg (QD) | - rTNSS | 4 weeks | 298 | 268 (89.9) | 172 (57.7) | 31.3 |
| Alvarado-Valdés CA, 1997 ^116^  (FLN-411) | US; 1991 (July – October) | Fluticasone propionate 200 μg (2 sprays/nostril QD)  Astemizole 10 mg (QD)  Placebo | - rTNSS | 2 weeks | 313 | 286 (91.4) | 136 (43.5) | 32.4 |
| Day J, 1997 ^117^ | Canada; 1991 (April-September) | Budesonide nasal spray 256 μg (2 sprays/nostril QD)  Budesonide nasal spray 400 μg (2 sprays/nostril QD)  Placebo | - rTNSS | 3 weeks | 240 | 228 (95.0) | 118 (49.2) | 31.3 |
| Gawchik SM, 1997 ^118^ | United States; - | Triamcinolone acetonide 220 mcg (2 sprays/nostril QD)  Loratadine 10 mg (QD) | - rTNSS | 4 weeks | 305 | 276 (90.5) | 174 (57) | 33.1 |
| Géhanno P, 1997 ^119^  (FNM40071) | France; 1991 (March 15 - August 20) | Fluticasone propionate 200 mcg (2 sprays/nostril QD)  Loratadine 10 mg (QD) | - rTNSS | 4 weeks | 114 | 103 (90.4) | 63 (55.3) | 39 |
| Small P, 1997 ^120^ | -; Spring allergy season | Triamcinolone nasal spray 220 μg (2 sprays/ nostril QD)  Fluticasone propionate 200 μg (2 sprays/nostril QD) | - rTNSS | 3 weeks | 233 | 223 (95.7) | 121 (51.9) | 28.6 |
| Stern MA, 1997 ^121^ | Denmark and UK; 1993 (April-July) | Budesonide nasal spray 128 μg (1 spray/nostril QD)  Budesonide nasal spray 256 μg (2 sprays/nostril QD)  Fluticasone propionate 200 μg (QD)  Placebo | - rTNSS | 4-6 weeks | 602 | 551 (91.5) | 266 (44.2) | - |
| Creticos P, 1998 ^122^ | US; 1994 (ragweed pollen season) | Budesonide nasal spray 32 μg (QD)  Budesonide nasal spray 64 μg (QD)  Budesonide nasal spray 128 μg (QD)  Budesonide nasal spray 256 μg (QD)  Placebo | - rTNSS | 4 weeks | 406 | 395 (97.3) | 182 (44.8) | 25.4 |
| Meltzer EO, 1998 ^123^ | Southern California, US; (April-August) | Mometasone 200 μg (2 sprays/nostril QD)  Placebo | - rTNSS | 2 weeks | 128 | 121 (94.5) | - | - |
| Ratner PH, 1998 ^124^  (FLTA4006) | US; 1995 (December) – 1996 (February) | Fluticasone propionate 200 μg (2 sprays/nostril QD)  Loratadine 10 mg (QD)  Fluticasone propionate 200 μg (2 sprays/nostril QD) + Loratadine 10 mg (QD)  Placebo | - rTNSS  - RQLQ | 2 weeks | 600 | 569 (94.8) | 328 (54.7) | 41.3 |
| Rosenthal R, 1998 ^125^ | US; grass pollen season | Triamcinolone nasal spray 200 μg (4 sprays/nostril QD)  Triamcinolone nasal spray 400 μg (4sprays/nostril QD)  Triamcinolone aerosol 440 μg (4 sprays/nostril QD)  Placebo | - rTNSS | 2 weeks | 377 | 355 (94.2) | 143 (37.9) | 34.2 |
| Stricker WE, 1998 ^126^  (FLTA4024) | US; 1997 | Fluticasone propionate 200 μg (QD)  Loratadine 10 mg (QD)  Fluticasone propionate 200 μg (2 sprays/nostril QD) + Loratadine 10 mg (QD)  Placebo | - rTNSS | 2 weeks | 655 | 636 (97.1) | 371 (56.6) | 34.0 |
| Berkowitz RB, 1999 ^127^  (NCT03861559) | 1994 (March-June) | Mometasone 200 μg (2 sprays/nostril QD)  Placebo | - rTNSS | 2 weeks | 201 | 194 (96.5) | 111 (55.2) | 31.4 |
| Casale TB, 1999 ^128^ | USA; 1997 (August – November) | Fexofenadine 120 mg (QD)  Fexofenadine 180 mg (QD)  Placebo | - RQLQ | 2 weeks | 861 | - | 557 (64.7) | 32 |
| Di Lorenzo G, 1999 ^129^ | Italy | Fluticasone propionate 200 μg (QD)  Levocabastine 200 μg (BID)  Placebo | - rTNSS | 6 weeks | 24 | 24 (100) | - | - |
| Hampel FC, 1999 ^130^ | Texas, US; 1994-1995 Mountain cedar allergy season | Levocabastine 400 μg (2 sprays/nostril BID)  Placebo | - rTNSS | 4 weeks | 270 | - | 175 (64.8) | 39.5 |
| Kremer B, 1999 ^131^ | 1993 (April-July) | Azelastine 560 μg (1 spray/nostril BID)  Azelastine 560 μg (1 spray/nostril BID) + azelastine 2 mg | -rTNSS | 2 weeks | 293 | 262 (89.4) | 139 (47.4) | 32.5 |
| Tanner LA, 1999 ^132^ | USA (32 sites); 1994 (March-July) | Fexofenadine 120 mg (BID)  Placebo | - RQLQ | 2 weeks | 776 | - | - | - |
| Condemi J, 2000 ^133^ | United States; Grass Pollen Allergy Season | Triamcinolone acetonide 220 mcg (2 sprays/nostril QD)  Loratadine 10 mg (QD) | - rTNSS - RQLQ | 4 weeks | 351 | 317 (90.3) | 193 (55) | 32 |
| Meltzer EO, 2000 ^134^ | California, USA (32 sites); 1997 (March-May) | Loratadine 10 mg (QD)  Montelukast 10 mg (QD)  Montelukast 20 mg (QD)  Loratadine 10 mg + montelukast 10 mg (QD)  Placebo | - rTNSS  - rTOSS  - RQLQ | 2 weeks | 458 | 432 (94.3) | 210 (45.8) | - |
| Van Cauwenberge P, 2000 ^135^ | Europe (nine countries) and South Africa; - | Fexofenadine 120 mg (QD)  Loratadine 10 mg (QD)  Placebo | - RQLQ | 2 weeks | 685 | 639 (93.3) | 379 (55.3) | 31.2 |
| Falser N, 2001 ^136^ | Australia; 1996 hay fever season | Azelastine 0.10% (2 sprays/ nostril BID)  Levocabastine 400 μg (2 sprays/nostril BID) | - rTNSS | 4 weeks | 180 | 177 (98.3) | 58 (32.2) | 27.0 |
| FNM30034 ^137^ | US; 2001 (March – June) | Fluticasone propionate 200 μg (2 sprays/nostril QD)  Loratadine 10 mg (QD)  Placebo | - rTOSS | 4 weeks | 482 | 444 (92.1) | 310 (64.3) | 34.9 |
| R1810221 ^138^ | US; 2002 (March – May) | Fluticasone propionate 100 μg (QD)  Placebo | - rTNSS | 2 weeks | 411 | 406 (98.8) | 266 (64.7) | 35.5 |
| NCT00641680 ^139^ | US; 2003 (April-July) | Budesonide nasal spray 64 μg (QD)  Fluticasone propionate 200 μg (QD)  Placebo | - rTNSS | 2 weeks | 318 | 304 (95.6) | - | - |
| Berger WE, 2002 ^140^ | USA (37 sites); 1999 (August) - 2000 (March) | Desloratadine 5 mg (QD)  Placebo | - rTNSS | 4 weeks | 331 | 284 (85.8) | 218 (65.9) | 32.3 |
| Ciprandi G, 2002 ^141^ | France and Italy | Budesonide 280 μg (1 spray/nostril QD)  Fluticasone propionate 200 μg (2 sprays/nostril QD)  Placebo | - rTNSS | 5 weeks | 307 | 280 (91.2) | 155 (50.5) | 34.7 |
| Gross G, 2002 ^142^ | Texas, US; 1996 Fall season | Triamcinolone nasal spray 220 μg (2 sprays/ nostril QD)  Fluticasone propionate 200 μg (2 sprays/nostril QD) | - rTNSS  - RQLQ | 3 weeks | 352 | 342 (97.2) | 234 (66.5) | 38.8 |
| Nayak AS, 2002 ^143^ | USA (14 sites); 1999 (September - November) | Loratadine 10 mg (QD)  Montelukast 10 mg (QD)  Loratadine 10 mg + montelukast 10 mg (QD)  Placebo | - rTNSS  - rTOSS  - RQLQ | 2 weeks | 897 | 762 (84.9) | 587 (65.4) | 37.4 |
| Philip G, 2002 ^144^ | North America (50 sites); 2000 (Spring) | Loratadine 10 mg (QD)  Montelukast 10 mg (QD)  Placebo | - rTNSS  - rTOSS  - RQLQ | 2 weeks | 1298 | 1239 (95.5) | 450 (34.7) | 36.4 |
| Salmun L, 2002 ^145^ | USA (29 sites); 1998 (April-June) | Desloratadine 2.5 mg (QD)  Desloratadine 5 mg (QD) ^e^  Desloratadine 7.5 mg (QD)  Desloratadine 10 mg (QD)  Desloratadine 20 mg (QD) Placebo | - rTNSS | 2 weeks | 1036 | 968 (93.4) | 613 (59.2) | 34 |
| Berger WE, 2003 ^146^ | US; 2001 Spring allergy season | Triamcinolone nasal spray 220 μg (2 sprays/ nostril QD)  Fluticasone propionate 200 μg (2 sprays/nostril QD) | - rTNSS  - RQLQ | 3 weeks | 295 | 287 (97.3) | 183 (62.0) | 31.6 |
| Berger WE, 2003 ^147^ | US; 2002 fall allergy season | Azelastine 0.10% (2 sprays/ nostril BID)  Azelastine 0.10% (2 sprays/ nostril BID) + loratadine 10 mg  Desloratadine 5 mg  Placebo | - rTNSS | 2 weeks | 440 | 428 (97.3) | 290 (65.9) | 35.2 |
| Dykewicz MS, 2003 ^148^  (FNM30030; FNM30031) | US; 2000 (August – October) | Fluticasone propionate 200 μg (QD)  Placebo | - rTNSS | 4 weeks | 241 | 224 (92.9) | 143 (59.3) | 34.3 |
| Gawchik S, 2003 ^149^ | US | Mometasone 200 μg (2 sprays/nostril QD)  Placebo | - rTNSS | 2 weeks | 245 | 241 (98.4) | 162 (66.1) | 34.4 |
| Lumry W, 2003 ^150^ | US; Fall ragweed pollen season | Triamcinolone nasal spray 220 μg (2 sprays/ nostril QD)  Beclomethasone nasal spray 336 μg (2 sprays/nostril BID) | - rTNSS  - RQLQ | 3 weeks | 152 | 145 (95.4) | 101 (66.4) | 37.0 |
| Noonan M, 2003 ^151^ | USA (19 sites); 1999 (Spring) | Cetirizine 10 mg (QD)  Placebo | - RQLQ | 2 weeks | 403 | 379 (94.0) | 265 (65.8) | 36.6 |
| Ratner PH, 2003 ^152^ (FNM40195) | United States; 2001 - 2002 Mountain Cedar Allergy Season (December 12 - February 26) | Fluticasone propionate 200 mcg (QD)  Montelukast 10 mg (QD) | - rTNSS | 2 weeks | 705 | 673 (95.5) | 439 (62.3) | 38.2 |
| van Adelsberg J, 2003a ^153^ | North America (32 sites); 2001 (Spring) | Loratadine 10 mg (QD)  Montelukast 10 mg (QD)  Placebo | - rTNSS  - rTOSS  - RQLQ | 2 weeks | 1214 | 1158 (95.4) | 764 (62.9) | 35.9 |
| van Adelsberg J, 2003b ^154^ | USA; 2001 (Fall) | Loratadine 10 mg (QD)  Montelukast 10 mg (QD)  Placebo | - rTNSS  - rTOSS  - RQLQ | 4 weeks | 1077 | 1000 (92.9) | 721 (66.9) | 36.4 |
| Bernstein DI, 2004 ^155^  (FNM30033) | US; 2001 (March – July) | Fluticasone propionate 200 μg (2 sprays/nostril QD)  Loratadine 10 mg (QD)  Placebo | - rTOSS | 4 weeks | 471 (90.7) | 427 | 280 | 33.6 |
| Chervinsky P, 2004 ^156^  (NCT00960141) | -; 2000 (Fall allergy season) | Loratadine 10 mg (QD)  Montelukast 10 mg (QD)  Placebo | - rTNSS  - rTOSS  - RQLQ | 2 weeks | 820 | 795 (97.0) | 568 (68.5) | 36.6 |
| Hampel F, 2004 ^157^ | USA (18 sites); 1999 (September -December) | Ebastine 10 mg (QD)  Ebastine 20 mg (QD)  Loratadine 10 mg (QD)  Placebo | - rTNSS | 4 weeks | 749 | 649 (86.6) | 385 (51.4) | 37.6 |
| LaForce C, 2004 ^158^ | US; 2003 spring allergy season | Azelastine 0.10% (2 sprays/nostril BID)  Azelastine 0.10% (2 sprays/nostril BID) + fexofenadine 120 mg (BID)  Placebo | - rTNSS | 2 weeks | 335 | 324 (96.7) | 207 (61.8) | 34.9 |
| Ratner P, 2004 ^159^ | USA (21 sites); 2001 (September - November) | Ebastine 20 mg (QD)  Loratadine 10 mg (QD)  Placebo | - rTNSS | 4 weeks | 703 | 618 (87.9) | 431 (61.3) | 38.2 |
| Bhatia S, 2005 ^160^ | United States; 2003 Spring Allergy Season (March - June) | Budesonide 64 mcg (QD)  Desloratadine 5 mg (QD) | - RQLQ | 2 weeks | 61 | 61 (100) | 33 (54.1) | 26 |
| Corren J, 2005 ^161^ | United States; 2004 Fall Allergy Season | Azelastine (2 sprays/nostril BID)  Cetirizine 10 mg (QD) | - rTNSS - RQLQ | 2 weeks | 307 | 299 (97.4) | 190 (35.5) | 35 |
| Meltzer EO, 2005 ^162^ | US; 2002 (August – November) | Olopatadine - 5320 μg (2 sprays/nostril BID)  Olopatadine - 3544 μg (2 sprays/nostril BID)  Placebo | - rTNSS  - RQLQ | 2 weeks | 565 | - | 349 | 35.2 |
| Okubo K, 2005 ^163^ | Tokyo, Japan; 2003 (February - March) | Fexofenadine 120 mg (BID)  Placebo | - RQLQ | 2 weeks | 210 | 206 (98.1) | 86 (41.0) | 33.5 |
| Ratner PH, 2005 ^164^ | Texas, US; 2002 (December) – 2003 (March) | Olopatadine - 5320 μg (2 sprays/nostril BID)  Olopatadine - 3544 μg (2 sprays/nostril BID)  Placebo | - rTNSS  - RQLQ | 2 weeks | 675 | - | 448 | 38.9 |
| Berger W, 2006 ^165^ | United States; 2005 Spring Allergy Season | Azelastine (2 sprays/nostril BID)  Cetirizine 10 mg (QD) | - rTNSS - RQLQ | 2 weeks | 354 | 345 (97.5) | 205 (57.9) | 35 |
| Martin BG, 2006 ^166^ | United States; 2001 - 2002 Mountain Cedar Allergy Season (December 11 - February 15) | Fluticasone propionate 200 mcg (QD)  Montelukast 10 mg (QD) | - rTNSS | 2 weeks | 736 | 698 (94.8) | 463 (63) | 39.7 |
| Meltzer EO, 2006 ^167^ | - | Desloratadine 5 mg (QD)  Placebo | - rTNSS  - RQLQ | 2 weeks | 220 | 212 (96.4) | 128 (58.2) | 32.2 |
| Ratner PH, 2006 ^168^ | US; 2002-2003  Mountain cedar allergy season | Ciclesonide nasal spray 20 μg (QD)  Ciclesonide nasal spray 50 μg (QD)  Ciclesonide nasal spray 100 μg (QD)  Ciclesonide nasal spray 200 μg (QD)  Placebo | - rTNSS | 2 weeks | 726 | 703 (96.8) | 512 (70.5) | - |
| Ratner PH, 2006 ^169^  (NCT00659841) | Texas, US; 2004-2005 Mountain cedar allergy season | Ciclesonide nasal spray 200 μg (2 sprays/nostril QD)  Placebo | - rTNSS  - RQLQ | 4 weeks | 327 | 292 (89.3) | 212 (64.8) | 40.0 |
| NCT00358527 ^170^ | 2006 (May-November) | Mometasone 200 μg (2 sprays/nostril QD)  Placebo | - rTNSS | 4 weeks | 401 | 385 (96.0) | 255 (63.6) | 35.7 |
| Fokkens WJ, 2007 ^171^  (NCT00225823) | Estonia, Latvia, Lithuania, Netherlands, Russia, Sweden; 2005 grass allergy season | Fluticasone furoate 110 μg (QD)  Placebo | - rTNSS  - rTOSS  - RQLQ | 2 weeks | 285 | 266 (93.3) | 151 (53.0) | 30.1 |
| Kaiser HB, 2007 ^172^  (NCT00197262) | US Midwest; 2005 ragweed allergy season (August-October) | Fluticasone furoate 110 μg (2 sprays/ nostril QD)  Placebo | - rTNSS  - rTOSS  - RQLQ | 2 weeks | 299 | 286 (95.7) | 180 (60.2) | 35.0 |
| Lumry W, 2007 ^173^ | US; 2003 spring allergy season | Azelastine 0.10% (1 spray/nostril BID)  Placebo | - rTNSS  - RQLQ | 2 weeks | 280 | 272 (97.1) | 177 (63.2) | 34.0 |
| Lumry W, 2007 ^173^ | US; 2003 spring allergy season | Azelastine 0.10% (1 spray/nostril BID)  Placebo | - rTNSS  - RQLQ | 2 weeks | 274 | 270 (98.5) | 171 (62.4) | 35.5 |
| Martin BG, 2007 ^174^  (FFR20001) | Texas, US; 2003-2004 mountain cedar allergy season | Fluticasone furoate 55 μg (2 sprays/ nostril QD)  Fluticasone furoate 110 μg (2 sprays/ nostril QD)  Fluticasone furoate 220 μg (2 sprays/ nostril QD)  Fluticasone furoate 440 μg (2 sprays/ nostril QD)  Placebo | - rTNSS  - rTOSS  - RQLQ | 2 weeks | 641 | 620 (96.7) | 421 (65.7) | 39.3 |
| Pradalier A, 2007 ^175^ | - | Desloratadine 5 mg (QD)  Placebo | - rTNSS  - RQLQ | 2 weeks | 483 | - | 233 (48.2) | 32.5 |
| NCT00453063 ^176^ | US; 2007 (March-June) | Mometasone 200 μg (2 sprays/nostril QD)  Placebo | - RQLQ | 2 weeks | 426 | 420 (98.6) | 270 (63.4) | - |
| Anolik R, 2008 ^177^ (NCT03855228) | US; 1995 (March-August) | Mometasone 200 μg (QD)  Loratadine 10 mg (QD)  Mometasone 200 μg (QD) + Loratadine 10 mg (QD)  Placebo | - rTNSS | 2 weeks | 692 | 665 (96.1) | 350 (49.9) | 25.5 |
| Ratner PH, 2008 ^178^ | US; December 2005 - February 2006 | Azelastine 0.10% (2 sprays/ nostril BID)  Fluticasone propionate 200 μg (2 sprays/nostril QD)  Azelastine 0.10% (2 sprays/nostril BID) + Fluticasone propionate 200 μg (2 sprays/nostril QD | - rTNSS  - RQLQ | 2 weeks | 151 | 147 (97.4) | 95 (62.9) | 37.2 |
| NCT00733005 ^179^ | US; 2008 (July-October) | Mometasone 200 μg (2 sprays/nostril QD)  Placebo | - rTNSS | 2 weeks | 324 | 319 (98.5) | 229 (70.7) | - |
| EudraCT 2008-000133-22 | Europe (103 sites); 2008 (May-August) | Desloratadine 5 mg (QD)  Placebo | - rTNSS - RQLQ | 2 weeks | 667 | 626 (93.9) | - | - |
| Andrews CP, 2009 ^180^  (NCT00502775) | US; 2007 ragweed allergy season | Fluticasone furoate 110 μg (QD)  Fexofenadine 180 mg (QD)  Placebo | - rTNSS  - rTOSS | 2 weeks | 679 | 642 (94.6) | 426 (62.6) | 34.4 |
| Andrews CP, 2009 ^180^  (NCT00435461) | Texas, US; 2006-2007 mountain cedar allergy season | Fluticasone furoate 110 μg (QD)  Fexofenadine 180 mg (QD)  Placebo | - rTNSS  - rTOSS | 2 weeks | 935 | 875 (93.6) | 602 (64.3) | 38.4 |
| Bachert C, 2009 ^181^ | Europe (eight countries); 2003 (April-August) | Bilastine 20 mg (QD)  Desloratadine 5 mg (QD)  Placebo | - rTNSS  - RQLQ | 2 weeks | 720 | 679 (94.3) | 355 (49.3) | 30.3 |
| Bernstein JA, 2009 ^182^ | US; 2006 spring allergy season | Azelastine 0.10% (1 spray/nostril BID)  Azelastine 0.10% (2 sprays/nostril BID)  Placebo | - rTNSS  - RQLQ | 2 weeks | 835 | 815 (97.6) | 495 (59.3) | 35.3 |
| Bousquet J, 2009 ^183^ | Europe and Canada (60 sites); 2006-2007 | Desloratadine 5 mg (QD)  Placebo | - RQLQ | 2 weeks | 547 | 518 (94.7) | 318 (58.1) | 34.2 |
| Brandi V, 2009 ^184^ | Texas, US; 2001 (December) – 2002 (February) | Fluticasone propionate 200 μg (QD)  Placebo | - rTNSS | 2 weeks | 519 | 498 (96.0) | 312 (60.1) | 37.9 |
| Demoly P, 2009 ^185^ | France (34 sites); 2002-2003 (cypress pollen season) | Desloratadine 5 mg (QD)  Placebo | - rTNSS  - RQLQ | 2 weeks | 233 | 133 (57.1) | 131 (56.2) | 39.8 |
| Jacobs R, 2009 ^186^  (NCT00115622) | Texas, US; 2004 December – 2005 January | Fluticasone furoate 110 μg (QD)  Placebo | - rTNSS  - rTOSS  - RQLQ | 2 weeks | 302 | 283 (93.7) | 191 (63.2) | 37.6 |
| Kaliner M, 2009 ^187^  (NCT00691665) | US; May 2008- January 2009 | Olopatadine 5320 μg (2 sprays/nostril BID)  Fluticasone propionate 200 μg (2 sprays/nostril QD) | - rTNSS  - rTOSS | 2 weeks | 130 | 130 (100.0) | 64 (49.2) | 35.3 |
| Kuna P, 2009 ^188^ | Europe (61 sites); 2005 (April-August) | Bilastine 20 mg (QD)  Cetirizine 10 mg (QD)  Placebo | - rTNSS  - rTOSS | 2 weeks | 681 | 646 (94.9) | 355 (52.1) | 31.1 |
| LaForce C, 2009 ^189^ | US | Ciclesonide nasal aerosol 80 μg (1 spray/nostril QD)  Ciclesonide nasal aerosol 160 μg (1 spray/nostril QD)  Ciclesonide nasal aerosol 320 μg (1 spray/nostril QD)  Placebo | - rTNSS  - RQLQ | 2 weeks | 513 | 498 (97.1) | 320 (62.4) | 37.7 |
| Lu S, 2009 ^190^ | US (17 sites); 1998 (April-June) | Beclomethasone 400 μg (BID)  Loratadine 10 mg (QD)  Montelukast 10 mg (QD)  Loratadine 10 mg + montelukast 10 mg (QD)  Placebo | - rTNSS  - RQLQ | 2 weeks | 628 | 617 (98.2) | 390  (62.1) | 34.8 |
| Lu S, 2009 ^191^ | US (21 sites); 1999 (April-June) | Loratadine 10 mg (QD)  Montelukast 10 mg (QD)  Loratadine 10 mg + montelukast 10 mg (QD)  Placebo | - rTNSS | 2 weeks | 530 | 518 (97.7) | 328 (61.9) | 28.3 |
| Nathan R, 2009 ^192^ | United States; - | Mometasone furoate 200 mcg (QD)  Loratadine 10 mg (QD) | - rTNSS - rTOSS | 2 weeks | - | 340 | - | - |
| Okubo K, 2009 ^193^  (FFR100652) | Japan; 2005 cedar allergy season | Fluticasone furoate 110 μg (QD)  Fluticasone propionate 200 μg (BID)  Placebo | - rTNSS | 2 weeks | 361 | 353 (97.8) | 188 (52.1) | 32.3 |
| Prenner B, 2009 ^194^  (NCT00319995) | US; 2006 Spring allergy season | Loratadine 10 mg (QD)  Montelukast 10 mg (QD)  Placebo | - RQLQ | 2 weeks | 1005 | 985 (98.0) | 698 (69.5) | 28.4 |
| Shah S, 2009 ^195^ (NCT00720278) | US; 2007 fall allergy season | Azelastine 0.15% (2 sprays/nostril BID)  Azelastine 0.10% (2 sprays/nostril BID)  Placebo | - rTNSS  - RQLQ | 2 weeks | 526 | 509 (96.8) | 334 (63.5) | 36.7 |
| Shah S, 2009 ^196^ | US; 2005 (May-October) | Azelastine 0.10% (2 sprays/nostril BID)  Olopatadine - 5320 μg (2 sprays/nostril BID)  Placebo | - rTNSS  - RQLQ | 2 weeks | 544 | - | 369 (67.8) | 36 |
| van Bavel J, 2009 ^197^  (NCT00660829) | US; 2007-2008 Mountain cedar allergy season | Azelastine 0.15% (2 sprays/nostril QD)  Placebo | - rTNSS  - RQLQ | 2 weeks | 532 | 499 (93.8) | 346 (65.0) | 40.3 |
| Hampel FC, 2010 ^198^  (NCT00660517) | Texas, US; 2007 Mountain cedar allergy season (January-February) | Azelastine 0.10% (1 spray/nostril BID)  Azelastine-fluticasone 137 μg (1 spray/ nostril BID)  Fluticasone propionate 200 μg (2 sprays/nostril QD)  Placebo | - rTNSS  - rTOSS  - RQLQ | 2 weeks | 609 | 577 (94.7) | 396 (65.0) | 39.2 |
| Maiti R, 2010 ^199^ | India (1 site); Winter | Rupatadine 10 mg (QD)  Levocetirizine 10 mg (QD) | - rTNSS  - RQLQ | 2 weeks | 60 | 51 (85.0) | 27 (45.0) | 29.6 |
| Mansfield L, 2010 ^200^ | USA (39 sites); 2008 (March-June) | Levocetirizine 5 mg (QD)  Placebo | - RQLQ | 2 weeks | 596 | 580 (97.3) | 405 (68.0) | 37.2 |
| Prenner BM, 2010 ^201^ (NCT00468312) | US; 2007 (April-July) | Mometasone 200 μg (2 sprays/nostril QD)  Placebo | - rTNSS  - rTOSS  - RQLQ | 2 weeks | 429 | 420 (97.9) | 257 (59.9) | 35.6 |
| Ratner P, 2010 ^202^  (NCT00790023) | Texas, US; 2008-2009 Mountain cedar allergy season | Ciclesonide nasal aerosol 80 μg (1 spray/nostril QD)  Ciclesonide nasal aerosol 160 μg (1 spray/nostril QD)  Placebo | - rTNSS  - rTOSS  - RQLQ | 2 weeks | 707 | 665 (94.1) | 469 (66.3) | 41.8 |
| Segall N, 2010 ^203^ | USA (39 sites); 2008 (Spring) | Levocetirizine 5 mg (QD)  Placebo | - rTNSS  - rTOSS  - RQLQ | 2 weeks | 580 | 566 (97.6) | 351 (60.5) | 38.1 |
| Han D, 2011 ^204^  (NCT01231464) | China; 2009 September – 2010 May | Fluticasone furoate 110 μg (QD)  Placebo | - rTNSS | 2 weeks | 137 | - | - | - |
| Howland WC, 2011^205^  (NCT00824473) | Texas, US; 2008-2009 Mountain cedar allergy season | Azelastine 0.15% (2 sprays/nostril QD)  Placebo | - rTNSS  - rTOSS  - RQLQ | 2 weeks | 506 | 478 (94.5) | 307 (60.7) | 38.2 |
| Maiti R, 2011 ^206^ | India (1 site) | Olopatadine 10 mg (QD)  Rupatadine 10 mg (QD) | - rTNSS  - RQLQ | 2 weeks | 70 | 57 (81.4) | 38 (54.3) | 37.6 |
| Meltzer EO, 2011^207^ (NCT00732381) | US; 2008 (August-November) | Mometasone 200 μg (2 sprays/nostril QD)  Placebo | - rTNSS | 2 weeks | 351 | 348 (99.1) | 223 (63.5) | - |
| Meltzer EO, 2011^207^ (NCT00728416) | US; 2008 (August-October) | Mometasone 200 μg (2 sprays/nostril QD)  Placebo | - rTNSS | 2 weeks | 333 | 329 (98.8) | 212 (63.7) | - |
| Diaz A, 2012 ^208^  (NCT00997620) | Texas, US; 2010 | Fluticasone furoate 110 μg (2 sprays/ nostril QD)  Placebo | - rTNSS | 2 weeks | 40 | 40 (100) | 20 (50.0) | 37.0 |
| Ratner PH, 2012 ^209^  (NCT01010971) | Texas, US; 2009-2010 Mountain cedar allergy season | Ciclesonide nasal aerosol 80 μg (1 spray/nostril QD)  Ciclesonide nasal aerosol 160 μg (1 spray/nostril QD)  Placebo | - rTNSS  - rTOSS  - RQLQ | 2 weeks | 671 | 647 (96.4) | 398 (59.3) | 40.4 |
| NCT01279057 ^210^ | Texas, US; 2010-2011 winter season | Fluticasone furoate 110 μg (2 sprays/ nostril QD)  Placebo | - rTNSS  - rTOSS | 2 weeks | 727 | 713 (98.1) | 460 (63.3) | - |
| van Bavel JH, 2012 ^211^  (NCT01024608) | US; 2009-2010 mountain cedar allergy season | Beclomethasone aerosol 320 μg (2 sprays/nostril QD)  Placebo | - rTNSS  - rTOSS  - RQLQ | 2 weeks | 340 | 332 (97.6) | 210 (61.8) | 38.6 |
| Carr W, 2012 ^212^  (NCT00651118) | US; 2008 (March-June) | Azelastine 0.10% (1 spray/nostril BID)  Azelastine-fluticasone 137 μg (1 spray/ nostril BID)  Fluticasone propionate 200 μg (2 sprays/nostril QD)  Placebo | - rTNSS  - rTOSS  - RQLQ | 2 weeks | 831 | 798 (96.0) | 532 (64.0) | 37.3 |
| Carr W, 2012 ^212^  (NCT00740792) | US; 2008 fall allergy season (August-November) | Azelastine 0.10% (1 spray/nostril BID)  Azelastine-fluticasone 137 μg (1 spray/ nostril BID)  Fluticasone propionate 200 μg (2 sprays/nostril QD)  Placebo | - rTNSS  - rTOSS  - RQLQ | 2 weeks | 776 | 739 (95.2) | 494 (63.7) | 37.8 |
| Carr W, 2012 ^212^  (NCT00883168) | US; 2009 (April-August) | Azelastine 0.10% (1 spray/nostril BID)  Azelastine-fluticasone 137 μg (1 spray/ nostril BID)  Fluticasone propionate 200 μg (2 sprays/nostril QD)  Placebo | - rTNSS  - rTOSS  - RQLQ | 2 weeks | 1791 | 1728 (96.5) | 1097 (61.3) | 35.2 |
| Lukat K, 2013 ^213^ | Europe (five countries, 26 sites); 2005 (April - August) | Desloratadine 5 mg (QD)  Rupatadine 10 mg (QD)  Placebo | - rTNSS  - rTOSS | 4 weeks | 379 | 320 (84.4) | 177 (46.7) | 31.5 |
| Meltzer EO, 2013 ^214^ (NCT00552110) | US; 2007 (December) -2008 (February) | Mometasone 200 μg (2 sprays/nostril QD)  Oxymetazoline 0.05% (2 sprays/ nostril BID)  Mometasone (2 sprays/nostril QD) + oxymetazoline (1 spray/nostril QD)  Mometasone (2 sprays/nostril QD) + oxymetazoline (3 sprays/nostril QD)  Placebo | - RQLQ | 2 weeks | 704 | 679 (96.3) | 471 (66.8) | 39.5 |
| Raphael GD, 2013 ^215^  (NCT00854360) | US; 2009 (March-May) | Beclomethasone aerosol 80 μg (1 spray/nostril QD)  Beclomethasone aerosol 160 μg (2 sprays/nostril QD)  Beclomethasone aerosol 320 μg (4 sprays/nostril QD)  Placebo | - rTNSS  - rTOSS  - RQLQ | 2 weeks | 487 | 470 (96.5) | 316 (64.9) | 38.5 |
| Schenkel E, 2013 ^216^ | US; ragweed pollen season | Triamcinolone CFC aerosol 14 μg (1 spray/nostril QD)  Triamcinolone CFC aerosol 110 μg (1 spray/nostril QD)  Triamcinolone CFC aerosol 440 μg (1 spray/nostril QD)  Triamcinolone HFA aerosol 14 μg (1 spray/nostril QD)  Triamcinolone HFA aerosol 110 μg (1 spray/nostril QD)  Triamcinolone HFA aerosol 440 μg (1 spray/nostril QD)  Placebo | - rTNSS | 2 weeks | 780 | 732 (93.8) | 314 (40.3) | 36 |
| NCT00719862 ^217^ | US; 2007 fall allergy season (August-November) | Azelastine 0.15% (2 sprays/nostril QD)  Placebo | - rTNSS  - RQLQ | 2 weeks | 480 | 467 (97.3) | 316 (65.8) | 35.4 |
| NCT00618332 ^218^ | US; 2008 (April-July) | Mometasone 200 μg (2 sprays/nostril QD)  Placebo | - RQLQ | 2 weeks | 40 | 40 (100) | 16 (40.0) | 33.8 |
| NCT01038427 ^219^ | Texas, US; 2009 (December) – 2010 (February) | Mometasone 200 μg (2 sprays/nostril QD)  Placebo | - rTNSS | 2 weeks | 795 | 782 (98.4) | 521 (65.5) | - |
| NCT02155881 ^220^ | Russia; 2014 (August-November) | Ciclesonide nasal spray 200 μg (2 sprays/nostril QD)  Placebo | - rTNSS  - rTOSS  - RQLQ | 2 weeks | 80 | 74 (92.5) | 45 (56.3) | 39.8 |
| NCT02230696 ^221^ | US; 2014-2015 | Azelastine-fluticasone 137 μg  Placebo | - rTNSS | 2 weeks | 951 | 921 (96.8) | 621 (65.3) | 38.1 |
| NCT01852513 ^222^ | Illinois, US; 2013 | Beclomethasone nasal spray 320 μg (2 sprays/nostril QD)  Placebo | - rTNSS  - RQLQ | 2 weeks | 39 | 39 (100) | 16 (41.0) | - |
| NCT01850823 ^223^ | US; 2013 (February-December) | Mometasone 200 μg (2 sprays/nostril QD)  Placebo | - rTNSS | 2 weeks | 880 | 864 (98.2) | 585 (66.5) | 39.0 |
| Kuna P, 2014 (EudraCT 2009-009094-98) ^224^ | Poland (Spring to Autumn) | Mometasone 200 μg (2 sprays/nostril QD)  Placebo | - rTNSS | 4 weeks | 402 | 380 (94.5) | 190 (47.3) | 31.2 |
| Ford LB, 2015 ^225^  (NCT01916226) | US; 2013 fall allergy season (August – October) | Fluticasone propionate 200 μg (2 sprays/nostril QD)  Cetirizine 10 mg (QD)  Placebo (separate groups for nasal and oral) | - rTNSS  - rTOSS | 2 weeks | 682 | 673 (97.8) | 428 (62.8) | 40.3 |
| Kim C-H, 2015 ^226^ (NCT01430260) | South Korea; 2011 - 2012 (January - July) | Ciclesonide 200 μg (QD)  Levocetirizine 5 mg (QD)  Ciclesonide 200 μg (QD) + Levocetirizine 5 mg (QD) | - rTNSS | 2 weeks | 73 | - | - | - |
| Ratner P, 2015 ^227^  (NCT01817790) | US; 2012-2013 mountain cedar pollen allergy season | Fluticasone propionate 200 μg (2 sprays/nostril QD)  Placebo | - rTOSS | 2 weeks | 626 | 614 (98.1) | 413 (66.0) | 40.4 |
| NCT02246920 ^228^ | US; 2014 (March – July) | Fluticasone propionate 200 μg  Placebo | - rTNSS | 2 weeks | 1178 | 1161 (98.6) | 720 (61.1) | 39.3 |
| Hisada S, 2016 ^229^ | Japan (3 sites); 2015 (January-April) | Desloratadine 5 mg (QD)  Placebo | - rTNSS | 2 weeks | 448 | 441 (98.4) | 261 (58.3) | 39.9 |
| Ratner P, 2017 ^230^  (NCT01241214) | Texas, USA (7 sites); Mountain Cedar pollen season | Cetirizine 10 mg (QD)  Placebo | - rTNSS  - rTOSS  - RQLQ | 2 weeks | 192 | 186 (96.9) | 136 (70.8) | 42.8 |
| Ratner P, 2017 ^230^  (NCT01484119) | Texas, USA (7 sites); Mountain Cedar pollen season | Cetirizine 10 mg (QD)  Placebo | - rTNSS  - rTOSS  - RQLQ | 2 weeks | 420 | 398 (94.8) | 254 (60.5) | 39.1 |
| Okubo K, 2018 ^231^ | Japan (4 sites); 2015 (February - May) | Rupatadine 10 mg (QD)  Rupatadine 20 mg (QD)  Placebo | - rTNSS  - rTOSS | 2 weeks | 900 | 893 (99.2) | 443 (49.2) | 36.7 |
| Gross GN, 2019 ^232^ (NCT02870205) | US; fall and mountain cedar allergy seasons | Mometasone 200 μg (2 sprays/nostril QD)  Olopatadine - 5320 μg (2 sprays/nostril BID)  Olopatadine-mometasone - 665 μg olopatadine + 25 μg mometasone (2 sprays/nostril BID)  Placebo | - rTNSS  - rTOSS  - RQLQ | 2 weeks | 1175 | 1147 (97.6) | 737 (62.7) | 39.6 |
| Hampel FC, 2019 ^233^ (NCT02631551) | US; 2016 spring pollen season | Mometasone 200 μg (2 sprays/nostril QD)  Olopatadine - 5320 μg (2 sprays/nostril BID)  Olopatadine-mometasone - 665 μg olopatadine + 25 μg mometasone (2 sprays/nostril BID)  Placebo | - rTNSS  - rTOSS  - RQLQ | 2 weeks | 1181 | 1126 (95.3) | 762 (64.5) | 39.3 |
| Ilyina NI, 2019 ^234^ | Russia; 2014 (April-July) | Azelastine 0.10% (1 spray/nostril BID)  Azelastine-fluticasone – 0.10% azelastine +200 μg fluticasone (1 spray/nostril BID) | - rTNSS  - rTOSS  - RQLQ | 2 weeks | 149 | 148 (99.3) | 77 (51.7) | 36.0 |
| Andrews CP, 2020 ^235^ (NCT02318303) | US; winter/mountain cedar pollen season | Mometasone 200 μg (2 sprays/nostril QD)  Mometasone 200 μg (2 sprays/nostril BID)  Olopatadine- 5320 μg (2 sprays/nostril BID)  Olopatadine - 2660 μg (2 sprays/nostril QD)  Olopatadine-mometasone - 665 μg olopatadine + 25 μg mometasone (2 sprays/nostril QD and BID)  Placebo | - rTNSS  - rTOSS  - RQLQ | 2 weeks | 1111 | 1085 (97.7) | 740 (66.6) | 43.9 |
| Lavorini F, 2020 ^236^  (NCT02761252) | Europe; Spring pollen season | Bilastine 20 mg (QD)  Montelukast 10 mg (QD)  Bilastine 20 mg (QD) + Montelukast 10 mg (QD) | - rTNSS  - rTOSS | 4 weeks | 419 | 388 (92.6) | 225 (53.6) | 35.2 |
| Haruna T, 2023 ^237^ | Japan; 2017 Japanese cedar pollen season | Mometasone 200 μg (1 spray/nostril QD)  Mometasone 200 μg (1 spray/nostril QD)  + levocabastine 400 μg (1 spray/nostril QID) | - rTNSS | 2 weeks | 40 | 40 (100) | 24 (60.0) | 41.6 |
| Gendeh HS, 2024 ^238^  (NCT05912192) | Malaysia | Mometasone 200 μg (1 spray/nostril BID)  Placebo | - RQLQ | 4 weeks | 163 | 155 (95.1) | 112 (68.7) | 31.2 |
| **Children with perennial allergic rhinitis** | | | | | | | | |
| Storms WW, 1996 ^239^ | US | Triamcinolone 220 μg (QD)  Placebo | - rTNSS | 4 weeks | 137 | 130 (94.9) | 49 (35.8) | 8.9  (6-11) |
| Fokkens WJ, 2002 ^240^ | Hungary, Netherlands, Portugal | Budesonide 128 μg (QD)  Placebo | - rTNSS  - RQLQ | 6 weeks | 202 | - | 63 (31.2) | 10.6  (6-16) |
| Lai DS, 2002 ^241^ | -; - | Cetirizine 10 mg (QD)  Oxatomide 1 mg/kg (BID)  Ketotifen 1 mg (QD)  Placebo | - RQLQ | 12 weeks | 80 | 69 (86.3) | 39 (48.8) | 8.1 |
| Potter PC, 2005 ^242^ | South Africa (24 sites); 2002 (April - September) | Levocetirizine 5 mg (QD)  Placebo | - RQLQ | 4 weeks | 306 | 297 (97.1) | 120 (39.2) | 9.9 |
| Chen ST, 2006 ^243^ | -; - | Cetirizine 5 mg (QD)  Montelukast 4 mg (QD)  Placebo | - RQLQ | 12 weeks | 60 | 60 (100) | 28 (46.7) | 4.5 |
| Murphy K, 2006 ^244^ | US; 2000 (January) – 2003 (April) | Budesonide 64 μg (1 spray/nostril QD)  Placebo | - RQLQ | 52 weeks | 229 | 168 (73.4) | 77 (33.6) | 5.9  (6-8) |
| Hung CH, 2007 ^245^ | Taiwan (single center); - | Loratadine 5 mg (QD)  Loratadine 5 mg + Montelukast 5 mg (QD)  Loratadine 5 mg + Sodium cromoglycate 20 mg/mL  Loratadine 5 mg + budesonide 200 μg | - rTNSS | 8 weeks | 45 | - | 22 (48.9) | 8.6 |
| Kim K, 2007 ^246^  (NCT00261287) | US; 2005 (November) – 2006 (June) | Ciclesonide spray 200 μg (QD)  Placebo | - rTNSS | 12 weeks | 123 | 123 (100) | 70 (56.9) | - |
| Kim K, 2007 ^246^  (NCT00658918) | US; 2004 (September) – 2005 (November) | Ciclesonide spray 25 μg (QD)  Ciclesonide spray 100 μg (QD)  Ciclesonide spray 200 μg (QD)  Placebo | - rTNSS | 6 weeks | 132 | 128 (97.0) | 58 (43.9) | 3.5  (2-5) |
| Berger WE, 2008 ^247^  (NCT00163514) | US; 2004 (May) – 2005 (July) | Ciclesonide spray 25 μg (QD)  Ciclesonide spray 100 μg (QD)  Ciclesonide spray 200 μg (QD)  Placebo | - rTNSS | 12 weeks | 664 | 586 (88.3) | 269 (40.5) | 9.0  (6-11) |
| Lee CF, 2008 ^248^ | Taiwan | Cetirizine 10 mg (QD)  Levocetirizine 5 mg (QD)  Placebo | - RQLQ | 12 weeks | 80 | 74 (92.5) | 31 (38.8) | 8.4 |
| Maspero JF, 2008 ^249^  (NCT00108914) | Argentina, Chile, Finland, Italy, Mexico, Slovakia, US; 2005 (February–November) | Fluticasone furoate 55 μg (QD)  Fluticasone furoate 110 μg (QD)  Placebo | - rTNSS | 12 weeks | 558 | 492 (88.2) | 248 (44.4) | 7.7  (2-11) |
| Tripathy I, 2008 ^250^  (NCT00116883) | US; 2005 (February-June) | Fluticasone furoate 100 μg (QD)  Placebo | - rTNSS | 6 weeks | 112 | 105 (93.8) | 56 (50.0) | 6.3  (2-11) |
| Li AM, 2009 ^251^ | -; - | Fexofenadine 60 mg (<12 years) or 120 mg (≥12 years) (QD) Fexofenadine 60 mg (<12 years) or 120 mg (≥12 years) (QD) + Montelukast 5 mg (<14 years) or 10 mg (≥14 years) (QD) | - rTNSS | 16 weeks | 44 | 44 (100) | 24 (54.5) | 12.1 |
| Weinstein S, 2009 ^252^  (NCT00132925) | Multicenter; 2004 (March) – 2006 (March) | Triamcinolone 110 μg (QD)  Placebo | - rTNSS | 4 weeks | 474 | 432 (91.1) | 198 (41.8) | 3.6  (2-5) |
| Baena-Cagnani CE, 2010 ^253^ | Argentina, Canada, Chile, Colombia, Finland, Guatemala, Mexico, Sweden, Uruguay, Venezuela | Mometasone 100 μg (QD)  Placebo | - rTNSS | 4 weeks | 381 | 357 (93.7) | 149 (39.1) | 7.5  (3-11) |
| Berman G, 2012 ^254^  (NCT01378429) | US; 2011 (July-November) | Ciclesonide aerosol 74 μg (QD)  Placebo | - rTNSS | 6 weeks | 89 | 88 (98.9) | 44 (49.4) | 8.9  (6-11) |
| Bernstein JA, 2014 ^255^  (NCT01018862) | US; 2009 (November) – 2011 (April) | Azelastine 0.10% (1 spray/nostril BID)  Azelastine 0.15% (1 spray/nostril BID)  Placebo | - rTNSS  - RQLQ | 4 weeks | 489 | 450 (92.0) | 208 (42.5) | 8.8  (6-11) |
| Lee L, 2014 ^256^  (NCT00570492) | Argentina, Canada, Chile, France, Peru, US; 2007 (November) – 2011 (March) | Fluticasone furoate 110 μg (QD)  Placebo | - rTNSS | 52 weeks | 474 | 373 (78.7) | 148 (31.2) | 6.6  (5-8) |
| Berger WE, 2015 ^257^  (NCT01783548) | US; 2013 (January-October) | Beclomethasone aerosol 80 μg (1 spray/nostril QD)  Placebo | - rTNSS | 12 weeks | 547 | 495 (90.5) | 246 (45.0) | 8.0  (4-11) |
| NCT01451541 | US; 2011 (October) – 2012 (December) | Ciclesonide aerosol 37 μg (QD)  Ciclesonide aerosol 74 μg (QD)  Placebo | - rTNSS  - RQLQ | 12 weeks | 846 | 748 (88.4) | 363 (42.9) | (6-11) |
| Kim CK, 2024 ^258^ | Korea (9 centres); 2019 (May) – 2022 (June) | Levocetirizine 5 mg (QD) + montelukast 5 mg (QD)  Montelukast 5 mg (QD) | - rTNSS | 4 weeks | 147 | 136 (92.5) | 49 (33.3) | 9.7  (6-14) |
| **Children with seasonal allergic rhinitis** | | | | | | | | |
| Masi M, 1993 ^259^ | Italy; 1990 (May-July) | Cetirizine 10 mg (BID)  Placebo | - rTNSS | 2 weeks | 124 | 114 (91.9) | 48 (38.7) | 10.1 |
| Galant S, 1994^260^  (FLN-320) | US; 1989 (August-November) | Fluticasone propionate 100 μg (QD)  Fluticasone propionate 200 μg (QD)  Placebo | - rTNSS | 2 weeks | 250 | 245 (98.0) | 87 (34.8) | 8.9  (4-11) |
| Banov CH, 1996^261^ | US; (April and July) | Triamcinolone 220 μg (QD)  Placebo | - rTNSS | 2 weeks | 116 | 115 (99.1) | 42 (36.2) | 9.0  (6-11) |
| Schenkel EJ, 1997 ^262^ | - | Triamcinolone 110 μg (QD)  Triamcinolone 220 μg (QD)  Placebo | - rTNSS  - rTOSS | 2 weeks | 223 | 204 (91.5) | 76 (34.1) | 9.0  (6-11) |
| Meltzer EO, 1999 ^263^  (NCT03879772) | - | Mometasone 25 μg (1 spray/nostril QD)  Mometasone 100 μg (1 spray/nostril QD)  Mometasone 200 μg (1 spray/nostril QD)  Beclomethasone 168 μg (1 spray/nostril BID)  Placebo | - rTNSS | 4 weeks | 679 | 646 (95.1) | 260 (38.3) | 9.0  (6-11) |
| de Blic J, 2005 ^264^ | France and Germany; 2002 (April-September) | Levocetirizine 5 mg (QD)  Placebo | - RQLQ | 6 weeks | 177 | 145 (81.9) | 60 (33.9) | 9.9 |
| Berger WE, 2008 ^247^  (NCT00305487) | US; 2006 (March-October) | Ciclesonide spray 100 μg (QD)  Ciclesonide spray 200 μg (QD)  Placebo | - rTNSS | 2 weeks | 618 | 588 (95.1) | 141 (22.8) | 9.7  (6-11) |
| Berger WE, 2009 ^265^  (NCT00578929) | US; 2006 (December) – 2008 (January) | Olopatadine 0.6%  (1 spray/nostril BID)  Olopatadine 0.6%  (2 spray/nostril BID)  Placebo | - rTNSS  - rTOSS  - RQLQ | 2 weeks | 1188 | 1135 (98.0) | 501 (42.2) | (6-11) |
| Meltzer E, 2009 ^266^  (NCT00107757) | US; 2005 (Spring and Fall allergy seasons) | Fluticasone furoate 55 μg (QD)  Fluticasone furoate 110 μg (QD)  Placebo | - rTNSS  - rTOSS | 2 weeks | 554 | 536 (96.8) | 231 (41.7) | 8.1  (2-11) |
| Storms WW, 2013 ^267^  (NCT01307319) | US; 2011 (March-August) | Beclomethasone spray 80 μg (1 spray/nostril QD)  Beclomethasone spray 160 μg (1 spray/nostril QD)  Placebo | - rTNSS | 2 weeks | 715 | 696 (97.3) | 332 (46.4) | 9.0  (6-11) |
| NCT01458275 | US; 2011 (November) – 2013 (March) | Ciclesonide aerosol 37 μg (QD)  Ciclesonide aerosol 74 μg (QD)  Placebo | - rTNSS  - rTOSS  - RQLQ | 2 weeks | 847 | 811 (95.7) | 374 (44.2) | 8.6  (6-11) |
| Berger W, 2016 ^268^  (NCT01915823) | US; 2013 (July) – 2014 (February) | Azelastine-fluticasone 548 μg + 200 μg (1 spray/nostril BID)  Placebo | - rTNSS  - rTOSS  - RQLQ | 2 weeks | 348 | 344 (98.9) | 162 (46.6) | 8.4  (4-11) |
| Prenner B, 2022 ^269^  (NCT03463031) | US; 2018 (March-November) | Olopatadine-mometasone 665 μg + 25 μg (1 spray/nostril BID)  Placebo | - rTNSS  - rTOSS  - RQLQ | 2 weeks | 446 | 431 (96.6) | 208 (46.6) | 8.7  (6-11) |

BID=Twice per day; QID=Four times per day; QD=Once per day; RQLQ=Rhinoconjunctivitis quality of life questionnaire; rTNSS=Reflective total nasal symptom score; rTOSS=Reflective total ocular symptom score; TID=Three times per day

**Supplementary Table 5. Results of the risk of bias assessment for each included primary study**

| **Study** | **Random sequence generation** | **Allocation concealment** | **Blinding of participants and personnel** | **Selective reporting** | **TNSS** | | **TOSS** | | **RQLQ** | |
| --- | --- | --- | --- | --- | --- | --- | --- | --- | --- | --- |
|  |  |  |  |  | **Blinding of outcome assessment** | **Incomplete outcome data** | **Blinding of outcome assessment** | **Incomplete outcome data** | **Blinding of outcome assessment** | **Incomplete outcome data** |
| **Adults with perennial allergic rhinitis** | | | | | | | | | | |
| Spector S, 1989 | UNCLEAR | UNCLEAR | LOW | UNCLEAR | LOW | HIGH |  |  |  |  |
| Day J, 1990 | UNCLEAR | UNCLEAR | LOW | UNCLEAR | LOW | UNCLEAR |  |  |  |  |
| Storms W, 1991 | UNCLEAR | UNCLEAR | LOW | UNCLEAR | LOW | HIGH |  |  |  |  |
| van As A, 1993 | LOW | LOW | LOW | LOW | LOW | HIGH |  |  |  |  |
| Banov CH, 1994 | UNCLEAR | UNCLEAR | UNCLEAR | UNCLEAR | LOW | HIGH |  |  |  |  |
| Kobayashi RH, 1995 | UNCLEAR | UNCLEAR | UNCLEAR | UNCLEAR | LOW | LOW |  |  |  |  |
| Drouin M, 1996 | LOW | LOW | LOW | HIGH | LOW | HIGH |  |  |  |  |
| Mandl M, 1997 | LOW | UNCLEAR | LOW | HIGH | LOW | HIGH |  |  |  |  |
| Bachert C, 1998 | UNCLEAR | UNCLEAR | LOW | UNCLEAR | LOW | LOW | LOW | LOW |  |  |
| Davies RJ, 1998 | LOW | UNCLEAR | LOW | UNCLEAR | LOW | LOW |  |  |  |  |
| Day J, 1998 | LOW | LOW | HIGH | LOW | HIGH | HIGH |  |  |  |  |
| Meltzer EO, 1998 | UNCLEAR | UNCLEAR | UNCLEAR | HIGH | LOW | LOW |  |  | UNCLEAR | UNCLEAR |
| Murris-Espin M, 1998 | UNCLEAR | UNCLEAR | LOW | UNCLEAR | LOW | LOW |  |  |  |  |
| Stern MA, 1998 | LOW | UNCLEAR | HIGH | LOW | HIGH | HIGH |  |  |  |  |
| Bousquet J, 1999 | UNCLEAR | UNCLEAR | UNCLEAR | UNCLEAR | LOW | UNCLEAR |  |  |  |  |
| Holm AF, 1999 | UNCLEAR | UNCLEAR | UNCLEAR | HIGH | LOW | HIGH |  |  |  |  |
| Kivisaari E, 2001 | UNCLEAR | UNCLEAR | UNCLEAR | LOW | LOW | LOW |  |  |  |  |
| Weckx LLM, 2001 | UNCLEAR | UNCLEAR | HIGH | UNCLEAR | HIGH | HIGH |  |  |  |  |
| Bende M, 2002 | LOW | UNCLEAR | UNCLEAR | UNCLEAR | UNCLEAR | UNCLEAR |  |  |  |  |
| Frèche C, 2002 | UNCLEAR | UNCLEAR | LOW | UNCLEAR | LOW | LOW |  |  |  |  |
| R1810220 | UNCLEAR | UNCLEAR | UNCLEAR | LOW | LOW | LOW |  |  |  |  |
| Simons F, 2003 | LOW | UNCLEAR | LOW | UNCLEAR | LOW | LOW |  |  |  |  |
| Bachert C, 2004 | LOW | LOW | UNCLEAR | UNCLEAR |  |  |  |  | LOW | LOW |
| Aguiar FAB, 2006 | UNCLEAR | UNCLEAR | UNCLEAR | UNCLEAR | LOW | UNCLEAR |  |  |  |  |
| Chervinsky P, 2007 | UNCLEAR | UNCLEAR | UNCLEAR | LOW | LOW | HIGH |  |  | LOW | HIGH |
| Meltzer EO, 2007 | UNCLEAR | UNCLEAR | UNCLEAR | LOW | LOW | UNCLEAR |  |  | LOW | UNCLEAR |
| Philip G, 2007 | LOW | UNCLEAR | LOW | UNCLEAR | LOW | LOW |  |  | LOW | LOW |
| Rosenblut A, 2007 | LOW | LOW | LOW | LOW | LOW | UNCLEAR |  |  |  |  |
| Fantin S, 2008 | LOW | LOW | LOW | UNCLEAR |  |  |  |  | LOW | LOW |
| Nathan RA, 2008 | LOW | LOW | LOW | UNCLEAR | LOW | LOW | LOW | LOW | LOW | UNCLEAR |
| Patel D, 2008 | LOW | LOW | LOW | LOW | LOW | HIGH |  |  |  |  |
| Vasar M, 2008 | LOW | LOW | LOW | LOW | LOW | HIGH | LOW | HIGH | LOW | HIGH |
| Holmberg K, 2009 | UNCLEAR | UNCLEAR | LOW | UNCLEAR | LOW | LOW |  |  | LOW | LOW |
| Kowalski ML, 2009 | LOW | UNCLEAR | LOW | UNCLEAR | LOW | LOW |  |  |  |  |
| Bernstein J, 2010 | UNCLEAR | UNCLEAR | UNCLEAR | LOW | LOW | LOW |  |  | LOW | LOW |
| Bousquet J, 2010 | LOW | UNCLEAR | LOW | LOW |  |  |  |  | LOW | HIGH |
| Given JT, 2010 | LOW | LOW | LOW | LOW | LOW | LOW | LOW | LOW | LOW | LOW |
| Grossman J, 2010 | UNCLEAR | UNCLEAR | HIGH | LOW | HIGH | HIGH |  |  |  |  |
| Meltzer EO, 2010 | UNCLEAR | UNCLEAR | UNCLEAR | LOW | LOW | LOW |  |  | LOW | LOW |
| Molina M, 2010 | UNCLEAR | UNCLEAR | LOW | UNCLEAR | LOW | LOW |  |  |  |  |
| NCT01033825 | UNCLEAR | UNCLEAR | UNCLEAR | LOW | LOW | LOW |  |  |  |  |
| Baroody FM, 2011 | UNCLEAR | UNCLEAR | UNCLEAR | LOW |  |  |  |  | LOW | LOW |
| Marmouz F, 2011 | UNCLEAR | UNCLEAR | LOW | HIGH | LOW | LOW |  |  |  |  |
| Berger WE, 2012 | LOW | LOW | UNCLEAR | LOW | LOW | LOW |  |  | LOW | HIGH |
| Fokkens WJ, 2012 (NCT00224523 \| FFR104503) | UNCLEAR | UNCLEAR | UNCLEAR | HIGH | UNCLEAR | LOW |  |  |  |  |
| Guilemany JM, 2012 | UNCLEAR | UNCLEAR | UNCLEAR | UNCLEAR |  |  |  |  | LOW | UNCLEAR |
| Meltzer EO, 2012 | UNCLEAR | UNCLEAR | UNCLEAR | LOW | LOW | LOW |  |  | LOW | LOW |
| Aneeza WH, 2013 | LOW | UNCLEAR | HIGH | UNCLEAR | HIGH | HIGH | HIGH | HIGH | HIGH | HIGH |
| EUCTR-2013-000654-22-DE | UNCLEAR | UNCLEAR | LOW | HIGH | LOW | LOW |  |  |  |  |
| LaForce C, 2013 | LOW | LOW | LOW | LOW | LOW | HIGH |  |  |  |  |
| Goh BS, 2014 | UNCLEAR | LOW | UNCLEAR | UNCLEAR | LOW | UNCLEAR | LOW | UNCLEAR | LOW | UNCLEAR |
| NCT00806754 | UNCLEAR | LOW | UNCLEAR | HIGH | LOW | LOW |  |  |  |  |
| Skoner D, 2014 | UNCLEAR | UNCLEAR | UNCLEAR | UNCLEAR |  |  |  |  | LOW | LOW |
| Weinstein SF, 2014 | UNCLEAR | UNCLEAR | UNCLEAR | LOW | LOW | UNCLEAR |  |  | LOW | HIGH |
| Jia M, 2017 | UNCLEAR | UNCLEAR | HIGH | UNCLEAR | HIGH | LOW |  |  |  |  |
| Lv C, 2018 | UNCLEAR | UNCLEAR | UNCLEAR | UNCLEAR | UNCLEAR | UNCLEAR |  |  |  |  |
| Karaulov AV, 2019 | UNCLEAR | UNCLEAR | LOW | LOW | LOW | LOW |  |  | LOW | LOW |
| Segall N, 2019 | UNCLEAR | UNCLEAR | UNCLEAR | HIGH | LOW | HIGH |  |  | LOW | HIGH |
| Mo JH, 2020 | LOW | LOW | LOW | LOW | LOW | HIGH |  |  |  |  |
| Wagner K, 2020 | UNCLEAR | UNCLEAR | UNCLEAR | UNCLEAR | UNCLEAR | UNCLEAR |  |  |  |  |
| Won T, 2021 | LOW | LOW | LOW | UNCLEAR | LOW | LOW |  |  |  |  |
| Bousquet J, 2025 | UNCLEAR | UNCLEAR | HIGH | UNCLEAR |  |  |  |  | LOW | HIGH |
| **Adults with seasonal allergic rhinitis** | | | | | | | | | | |
| Andersen JB, 1975 | UNCLEAR | UNCLEAR | LOW | UNCLEAR | LOW | HIGH |  |  |  |  |
| Munch EP, 1983 | UNCLEAR | UNCLEAR | LOW | UNCLEAR | LOW | LOW |  |  |  |  |
| Gutkowski A, 1985 | UNCLEAR | UNCLEAR | UNCLEAR | UNCLEAR | LOW | HIGH | LOW | HIGH |  |  |
| Skassa-Brociek W, 1988 | UNCLEAR | UNCLEAR | UNCLEAR | UNCLEAR | LOW | LOW |  |  |  |  |
| Tinkelman D, 1989 | UNCLEAR | UNCLEAR | UNCLEAR | UNCLEAR | LOW | LOW |  |  |  |  |
| Meltzer EO, 1990 | LOW | LOW | UNCLEAR | LOW | LOW | LOW |  |  |  |  |
| Nathan RA, 1991 | UNCLEAR | UNCLEAR | UNCLEAR | LOW | LOW | LOW |  |  |  |  |
| van As A, 1991 | UNCLEAR | UNCLEAR | UNCLEAR | UNCLEAR | LOW | LOW |  |  |  |  |
| FLN-270 | UNCLEAR | UNCLEAR | UNCLEAR | HIGH | LOW | LOW |  |  |  |  |
| Findlay S, 1991 | UNCLEAR | UNCLEAR | LOW | UNCLEAR | LOW | LOW |  |  |  |  |
| Norman PS, 1992 | UNCLEAR | UNCLEAR | LOW | UNCLEAR | LOW | LOW |  |  |  |  |
| Ratner PH, 1992 | UNCLEAR | UNCLEAR | LOW | LOW | LOW | LOW |  |  |  |  |
| Dockhorn RJ, 1993 | UNCLEAR | UNCLEAR | UNCLEAR | LOW | LOW | LOW |  |  |  |  |
| NCT03855189 | UNCLEAR | UNCLEAR | UNCLEAR | LOW | LOW | LOW |  |  |  |  |
| Dahl R, 1994 | UNCLEAR | UNCLEAR | UNCLEAR | UNCLEAR | LOW | UNCLEAR | LOW | UNCLEAR |  |  |
| LaForce C, 1994 | UNCLEAR | UNCLEAR | UNCLEAR | LOW | LOW | LOW |  |  |  |  |
| Mackowiak J, 1994 | UNCLEAR | UNCLEAR | UNCLEAR | LOW | LOW | LOW |  |  |  |  |
| Mcarthur JG, 1994 | UNCLEAR | UNCLEAR | HIGH | UNCLEAR | UNCLEAR | HIGH |  |  |  |  |
| Munk ZM, 1994 | UNCLEAR | UNCLEAR | UNCLEAR | LOW | LOW | LOW |  |  |  |  |
| van Bavel J, 1994 | LOW | UNCLEAR | UNCLEAR | UNCLEAR | LOW | LOW |  |  |  |  |
| NCT03882047 | UNCLEAR | UNCLEAR | LOW | LOW | LOW | LOW |  |  |  |  |
| Bronsky E, 1995 | UNCLEAR | UNCLEAR | LOW | UNCLEAR | LOW | LOW |  |  |  |  |
| Pelucchi A, 1995 | UNCLEAR | UNCLEAR | UNCLEAR | HIGH | LOW | HIGH |  |  |  |  |
| Prenner BM, 1995 | UNCLEAR | UNCLEAR | UNCLEAR | HIGH | LOW | HIGH |  |  |  |  |
| Settipane G, 1995 | UNCLEAR | UNCLEAR | HIGH | UNCLEAR | HIGH | HIGH |  |  |  |  |
| Bernstein DI, 1997 | UNCLEAR | UNCLEAR | UNCLEAR | UNCLEAR | UNCLEAR | UNCLEAR |  |  |  |  |
| Bronsky EA, 1996 | UNCLEAR | UNCLEAR | UNCLEAR | LOW | LOW | LOW |  |  |  |  |
| Cook CK, 1996 | UNCLEAR | UNCLEAR | UNCLEAR | LOW | LOW | LOW |  |  |  |  |
| Harvey R, 1996 | UNCLEAR | UNCLEAR | UNCLEAR | UNCLEAR |  |  |  |  | LOW | UNCLEAR |
| Hebert JR, 1996 | UNCLEAR | UNCLEAR | LOW | HIGH | LOW | HIGH |  |  |  |  |
| Howland WC, 1996 | UNCLEAR | UNCLEAR | UNCLEAR | LOW | LOW | LOW |  |  |  |  |
| Munk ZM, 1996 | UNCLEAR | UNCLEAR | UNCLEAR | UNCLEAR | LOW | LOW |  |  |  |  |
| Schoenwetter W, 1996 | UNCLEAR | UNCLEAR | LOW | UNCLEAR | LOW | LOW |  |  |  |  |
| Alvarado-Valdés CA, 1997 | UNCLEAR | UNCLEAR | LOW | HIGH | LOW | LOW |  |  |  |  |
| Day J, 1997 | LOW | LOW | HIGH | HIGH | HIGH | LOW |  |  |  |  |
| Gawchik SM, 1997 | UNCLEAR | UNCLEAR | LOW | UNCLEAR | LOW | UNCLEAR |  |  |  |  |
| Géhanno P, 1997 | UNCLEAR | UNCLEAR | LOW | UNCLEAR | LOW | LOW |  |  |  |  |
| Small P, 1997 | UNCLEAR | UNCLEAR | HIGH | UNCLEAR | HIGH | LOW |  |  |  |  |
| Stern MA, 1997 | UNCLEAR | UNCLEAR | HIGH | LOW | HIGH | HIGH |  |  |  |  |
| Creticos P, 1998 | UNCLEAR | UNCLEAR | UNCLEAR | HIGH | LOW | LOW |  |  |  |  |
| Meltzer EO, 1998 | UNCLEAR | UNCLEAR | UNCLEAR | HIGH | LOW | LOW |  |  |  |  |
| Ratner PH, 1998 | UNCLEAR | UNCLEAR | LOW | HIGH | LOW | LOW |  |  | LOW | LOW |
| Rosenthal R, 1998 | UNCLEAR | UNCLEAR | HIGH | LOW | HIGH | LOW |  |  |  |  |
| Stricker WE, 1998 | UNCLEAR | UNCLEAR | UNCLEAR | LOW | LOW | LOW |  |  |  |  |
| Berkowitz RB, 1999 | LOW | LOW | UNCLEAR | LOW | LOW | LOW |  |  |  |  |
| Casale TB, 1999 | UNCLEAR | UNCLEAR | UNCLEAR | HIGH |  |  |  |  | LOW | LOW |
| Di Lorenzo G, 1999 | UNCLEAR | UNCLEAR | UNCLEAR | LOW | LOW | UNCLEAR |  |  |  |  |
| Hampel FC, 1999 | UNCLEAR | UNCLEAR | UNCLEAR | UNCLEAR | LOW | LOW | LOW | LOW |  |  |
| Kremer B, 1999 | UNCLEAR | UNCLEAR | UNCLEAR | UNCLEAR | LOW | LOW |  |  |  |  |
| Tanner LA, 1999 | UNCLEAR | UNCLEAR | UNCLEAR | HIGH |  |  |  |  | UNCLEAR | HIGH |
| Condemi J, 2000 | UNCLEAR | UNCLEAR | LOW | UNCLEAR | LOW | LOW |  |  | LOW | LOW |
| Meltzer EO, 2000 | LOW | UNCLEAR | LOW | UNCLEAR | LOW | LOW | LOW | LOW | LOW | LOW |
| Van Cauwenberge P, 2000 | UNCLEAR | UNCLEAR | UNCLEAR | UNCLEAR |  |  |  |  | LOW | LOW |
| Falser N, 2001 | UNCLEAR | UNCLEAR | UNCLEAR | UNCLEAR | HIGH | LOW |  |  |  |  |
| FNM30034 | LOW | UNCLEAR | LOW | LOW |  |  | LOW | LOW |  |  |
| R1810221 | UNCLEAR | UNCLEAR | UNCLEAR | LOW | LOW | LOW |  |  |  |  |
| NCT00641680 | UNCLEAR | UNCLEAR | UNCLEAR | HIGH | LOW | LOW |  |  |  |  |
| Berger WE, 2002 | UNCLEAR | UNCLEAR | UNCLEAR | UNCLEAR | LOW | LOW |  |  |  |  |
| Ciprandi G, 2002 | LOW | LOW | LOW | LOW | LOW | HIGH |  |  |  |  |
| Gross G, 2002 | UNCLEAR | UNCLEAR | HIGH | UNCLEAR | HIGH | LOW |  |  | HIGH | LOW |
| Nayak AS, 2002 | LOW | UNCLEAR | LOW | LOW | LOW | LOW | LOW | LOW | LOW | LOW |
| Philip G, 2002 | LOW | UNCLEAR | UNCLEAR | HIGH | LOW | LOW | LOW | LOW | LOW | LOW |
| Salmun L, 2002 | LOW | UNCLEAR | LOW | UNCLEAR | LOW | LOW |  |  |  |  |
| Berger WE, 2003 | UNCLEAR | UNCLEAR | UNCLEAR | UNCLEAR | HIGH | LOW |  |  | HIGH | HIGH |
| Berger WE, 2003 | UNCLEAR | UNCLEAR | LOW | LOW | LOW | LOW |  |  |  |  |
| Dykewicz MS, 2003 | UNCLEAR | UNCLEAR | UNCLEAR | HIGH | LOW | LOW |  |  |  |  |
| Gawchik S, 2003 | UNCLEAR | UNCLEAR | UNCLEAR | LOW | LOW | LOW |  |  |  |  |
| Lumry W, 2003 | UNCLEAR | UNCLEAR | HIGH | UNCLEAR | HIGH | LOW |  |  | HIGH | HIGH |
| Noonan M, 2003 | UNCLEAR | UNCLEAR | UNCLEAR | UNCLEAR |  |  |  |  | LOW | LOW |
| Ratner PH, 2003 | LOW | HIGH | LOW | LOW | LOW | LOW |  |  |  |  |
| van Adelsberg J, 2003a | LOW | UNCLEAR | LOW | LOW | LOW | LOW | LOW | LOW | LOW | LOW |
| van Adelsberg J, 2003b | UNCLEAR | UNCLEAR | LOW | LOW | LOW | LOW | LOW | LOW | LOW | LOW |
| Bernstein DI, 2004 | LOW | LOW | LOW | LOW |  |  | LOW | LOW |  |  |
| Chervinsky P, 2004 | LOW | UNCLEAR | UNCLEAR | HIGH | LOW | LOW | LOW | LOW | LOW | LOW |
| Hampel F, 2004 | UNCLEAR | UNCLEAR | UNCLEAR | UNCLEAR | LOW | LOW |  |  |  |  |
| LaForce C, 2004 | UNCLEAR | UNCLEAR | LOW | LOW | LOW | LOW |  |  |  |  |
| Ratner P, 2004 | UNCLEAR | UNCLEAR | LOW | UNCLEAR | LOW | LOW |  |  |  |  |
| Bhatia S, 2005 | LOW | UNCLEAR | LOW | UNCLEAR |  |  |  |  | LOW | LOW |
| Corren J, 2005 | LOW | LOW | LOW | UNCLEAR | LOW | LOW |  |  | LOW | LOW |
| Meltzer EO, 2005 | UNCLEAR | UNCLEAR | UNCLEAR | UNCLEAR | LOW | UNCLEAR |  |  | LOW | UNCLEAR |
| Okubo K, 2005 | UNCLEAR | UNCLEAR | UNCLEAR | UNCLEAR |  |  |  |  | LOW | LOW |
| Ratner PH, 2005 | UNCLEAR | UNCLEAR | UNCLEAR | UNCLEAR | LOW | UNCLEAR |  |  | LOW | UNCLEAR |
| Berger W, 2006 | LOW | LOW | LOW | UNCLEAR | LOW | LOW |  |  | LOW | LOW |
| Martin BG, 2006 | UNCLEAR | UNCLEAR | LOW | UNCLEAR | LOW | LOW |  |  |  |  |
| Meltzer EO, 2006 | UNCLEAR | UNCLEAR | UNCLEAR | UNCLEAR | LOW | HIGH |  |  | LOW | HIGH |
| Ratner PH, 2006A | UNCLEAR | UNCLEAR | UNCLEAR | LOW | LOW | LOW |  |  |  |  |
| Ratner PH, 2006B | UNCLEAR | UNCLEAR | UNCLEAR | LOW | LOW | LOW |  |  | LOW | LOW |
| NCT00358527 | UNCLEAR | UNCLEAR | UNCLEAR | LOW | LOW | LOW |  |  |  |  |
| Fokkens WJ, 2007 | LOW | LOW | LOW | UNCLEAR | LOW | HIGH | LOW | HIGH | LOW | HIGH |
| Kaiser HB, 2007 | LOW | LOW | LOW | UNCLEAR | LOW | LOW | LOW | LOW | LOW | LOW |
| Lumry W, 2007A | LOW | LOW | UNCLEAR | LOW | LOW | LOW |  |  | LOW | HIGH |
| Lumry W, 2007B | LOW | LOW | UNCLEAR | LOW | LOW | LOW |  |  | LOW | HIGH |
| Martin BG, 2007 | LOW | LOW | LOW | LOW | LOW | LOW | LOW | LOW | LOW | LOW |
| Pradalier A, 2007 | LOW | UNCLEAR | UNCLEAR | UNCLEAR | LOW | UNCLEAR |  |  | LOW | UNCLEAR |
| NCT00453063 | UNCLEAR | UNCLEAR | UNCLEAR | LOW |  |  |  |  | LOW | HIGH |
| Anolik R, 2008 | LOW | LOW | UNCLEAR | LOW | LOW | LOW |  |  |  |  |
| Ratner PH, 2008 | LOW | LOW | LOW | UNCLEAR | LOW | LOW |  |  | LOW | LOW |
| NCT00733005 | UNCLEAR | UNCLEAR | UNCLEAR | LOW | LOW | LOW |  |  |  |  |
| EudraCT 2008-000133-22 | UNCLEAR | UNCLEAR | UNCLEAR | LOW | LOW | LOW |  |  | LOW | LOW |
| Andrews CP, 2009 [NCT00435461] | LOW | LOW | LOW | LOW | LOW | LOW | LOW | LOW |  |  |
| Andrews CP, 2009 [NCT00502775] | LOW | LOW | LOW | LOW | LOW | LOW | LOW | LOW |  |  |
| Bachert C, 2009 | LOW | LOW | LOW | UNCLEAR | LOW | LOW |  |  | LOW | LOW |
| Bernstein JA, 2009 | LOW | UNCLEAR | UNCLEAR | HIGH | LOW | LOW |  |  | LOW | UNCLEAR |
| Bousquet J, 2009 | LOW | UNCLEAR | LOW | LOW |  |  |  |  | LOW | LOW |
| Brandi V, 2009 | UNCLEAR | UNCLEAR | LOW | LOW | LOW | HIGH |  |  |  |  |
| Demoly P, 2009 | UNCLEAR | UNCLEAR | UNCLEAR | LOW | LOW | LOW |  |  | LOW | LOW |
| Jacobs R, 2009 | LOW | LOW | LOW | UNCLEAR | LOW | UNCLEAR | LOW | UNCLEAR | LOW | UNCLEAR |
| Kaliner MA, 2009 | UNCLEAR | UNCLEAR | LOW | LOW | LOW | LOW | LOW | LOW |  |  |
| Kuna P, 2014 | UNCLEAR | LOW | LOW | UNCLEAR | LOW | LOW |  |  |  |  |
| LaForce C, 2009 | LOW | LOW | LOW | UNCLEAR | LOW | LOW |  |  |  |  |
| Lu S, 2009a | LOW | LOW | UNCLEAR | HIGH | LOW | LOW |  |  |  |  |
| Lu S, 2009b | LOW | UNCLEAR | UNCLEAR | HIGH | LOW | LOW |  |  |  |  |
| Nathan R, 2009 | UNCLEAR | UNCLEAR | UNCLEAR | UNCLEAR | LOW | UNCLEAR | LOW | UNCLEAR |  |  |
| Okubo K, 2009 | UNCLEAR | UNCLEAR | HIGH | HIGH | LOW | LOW |  |  |  |  |
| Prenner B, 2009 | UNCLEAR | UNCLEAR | UNCLEAR | UNCLEAR |  |  |  |  | LOW | LOW |
| Shah S, 2009A | LOW | UNCLEAR | UNCLEAR | LOW | LOW | LOW |  |  | LOW | LOW |
| Shah S, 2009B | LOW | LOW | LOW | LOW | LOW | UNCLEAR |  |  | LOW | UNCLEAR |
| van Bavel J, 2009 | LOW | UNCLEAR | UNCLEAR | LOW | LOW | LOW |  |  | LOW | LOW |
| Hampel FC, 2010 | LOW | UNCLEAR | UNCLEAR | HIGH | LOW | LOW | LOW | LOW | LOW | LOW |
| Maiti R, 2010 | UNCLEAR | UNCLEAR | HIGH | UNCLEAR |  |  |  |  | HIGH | LOW |
| Mansfield L, 2010 | UNCLEAR | UNCLEAR | LOW | LOW |  |  |  |  | LOW | LOW |
| Prenner BM, 2010 | LOW | LOW | LOW | LOW | LOW | LOW | LOW | LOW | LOW | HIGH |
| Ratner P, 2010 | LOW | LOW | UNCLEAR | LOW | LOW | LOW | LOW | HIGH | LOW | HIGH |
| Segall N, 2010 | UNCLEAR | UNCLEAR | UNCLEAR | LOW | LOW | LOW | LOW | LOW | LOW | LOW |
| Han D, 2011 | LOW | LOW | LOW | LOW | LOW | UNCLEAR |  |  |  |  |
| Howland WC, 2011 | LOW | LOW | UNCLEAR | UNCLEAR | LOW | LOW | LOW | LOW | LOW | LOW |
| Maiti R, 2011 | LOW | UNCLEAR | HIGH | UNCLEAR | HIGH | LOW |  |  | HIGH | LOW |
| Meltzer EO, 2011 | LOW | UNCLEAR | UNCLEAR | LOW | LOW | LOW |  |  |  |  |
| Diaz A, 2012 | UNCLEAR | UNCLEAR | UNCLEAR | LOW | LOW | LOW |  |  |  |  |
| Ratner PH, 2012 | UNCLEAR | UNCLEAR | UNCLEAR | LOW | LOW | LOW | LOW | HIGH | LOW | HIGH |
| NCT01279057 | UNCLEAR | UNCLEAR | UNCLEAR | LOW | LOW | LOW | LOW | LOW |  |  |
| van Bavel JH, 2012 | UNCLEAR | UNCLEAR | UNCLEAR | LOW | LOW | LOW | LOW | LOW | LOW | HIGH |
| Carr W, 2012A | LOW | LOW | LOW | UNCLEAR | LOW | LOW | LOW | LOW | LOW | LOW |
| Carr W, 2012B | LOW | LOW | LOW | UNCLEAR | LOW | LOW | LOW | LOW | LOW | LOW |
| Carr W, 2012C | LOW | LOW | LOW | UNCLEAR | LOW | LOW | LOW | LOW | LOW | LOW |
| Lukat K, 2013 | UNCLEAR | UNCLEAR | UNCLEAR | UNCLEAR | LOW | LOW | LOW | LOW |  |  |
| Meltzer EO, 2013 | UNCLEAR | HIGH | LOW | HIGH |  |  |  |  | UNCLEAR | LOW |
| Raphael GD, 2013 | UNCLEAR | UNCLEAR | LOW | HIGH | LOW | LOW | LOW | HIGH | LOW | LOW |
| Schenkel E, 2013 | UNCLEAR | UNCLEAR | UNCLEAR | LOW | LOW | LOW |  |  |  |  |
| NCT00719862 | UNCLEAR | UNCLEAR | UNCLEAR | LOW | LOW | LOW |  |  | LOW | LOW |
| NCT00618332 | UNCLEAR | UNCLEAR | UNCLEAR | LOW |  |  |  |  | LOW | LOW |
| NCT01038427 | UNCLEAR | UNCLEAR | UNCLEAR | LOW | LOW | LOW |  |  |  |  |
| NCT02155881 | UNCLEAR | UNCLEAR | LOW | LOW | LOW | LOW | LOW | LOW | LOW | LOW |
| NCT02230696 | UNCLEAR | UNCLEAR | UNCLEAR | LOW | LOW | UNCLEAR |  |  |  |  |
| NCT01852513 | UNCLEAR | UNCLEAR | UNCLEAR | UNCLEAR | LOW | LOW |  |  | LOW | LOW |
| NCT01850823 | UNCLEAR | UNCLEAR | UNCLEAR | LOW | LOW | LOW |  |  |  |  |
| Kuna P, 2009 | LOW | UNCLEAR | LOW | UNCLEAR | LOW | LOW | LOW | LOW |  |  |
| Ford LB, 2015 | UNCLEAR | UNCLEAR | UNCLEAR | LOW | LOW | LOW | LOW | LOW |  |  |
| Kim CH, 2015 | LOW | HIGH | HIGH | LOW | HIGH | LOW |  |  |  |  |
| Ratner P, 2015 | UNCLEAR | UNCLEAR | UNCLEAR | LOW |  |  | LOW | LOW |  |  |
| NCT02246920 | UNCLEAR | UNCLEAR | UNCLEAR | LOW | LOW | LOW |  |  |  |  |
| Hisada S, 2016 | UNCLEAR | UNCLEAR | UNCLEAR | LOW | LOW | UNCLEAR |  |  |  |  |
| Ratner P, 2017 [NCT01241214] | LOW | LOW | LOW | LOW | LOW | LOW | LOW | LOW | LOW | LOW |
| Ratner P, 2017 [NCT01484119] | LOW | LOW | LOW | LOW | LOW | LOW | LOW | LOW | LOW | LOW |
| Okubo K, 2018 | LOW | LOW | UNCLEAR | LOW | UNCLEAR | LOW | UNCLEAR | LOW |  |  |
| Gross GN, 2019 | UNCLEAR | UNCLEAR | UNCLEAR | UNCLEAR | LOW | LOW | LOW | LOW | LOW | UNCLEAR |
| Hampel FC, 2019 | UNCLEAR | UNCLEAR | UNCLEAR | HIGH | LOW | LOW | LOW | LOW | LOW | LOW |
| Ilyina NI, 2019 | UNCLEAR | UNCLEAR | HIGH | UNCLEAR | HIGH | LOW | HIGH | LOW | HIGH | LOW |
| Andrews CP, 2020 | UNCLEAR | UNCLEAR | UNCLEAR | HIGH | LOW | LOW | LOW | HIGH | LOW | HIGH |
| Lavorini F, 2020 | UNCLEAR | UNCLEAR | LOW | LOW | LOW | LOW | LOW | LOW |  |  |
| Haruna T, 2023 | LOW | LOW | LOW | HIGH | LOW | LOW |  |  |  |  |
| Gendeh HS, 2024 | LOW | UNCLEAR | LOW | LOW |  |  |  |  | LOW | LOW |
| **Children with perennial allergic rhinitis** | | | | | | | | | | |
| Storms WW, 1996 | UNCLEAR | UNCLEAR | UNCLEAR | UNCLEAR | UNCLEAR | LOW |  |  |  |  |
| Fokkens WJ, 2002 | LOW | LOW | LOW | UNCLEAR | LOW | UNCLEAR |  |  | LOW | UNCLEAR |
| Lai DS, 2002 | LOW | UNCLEAR | UNCLEAR | UNCLEAR |  |  |  |  | LOW | LOW |
| Potter PC, 2005 | UNCLEAR | UNCLEAR | UNCLEAR | UNCLEAR |  |  |  |  | LOW | LOW |
| Chen ST, 2006 | UNCLEAR | UNCLEAR | UNCLEAR | UNCLEAR |  |  |  |  | LOW | LOW |
| Murphy K, 2006 | UNCLEAR | UNCLEAR | UNCLEAR | LOW |  |  |  |  | LOW | HIGH |
| Hung CH, 2007 | UNCLEAR | UNCLEAR | HIGH | UNCLEAR | HIGH | LOW |  |  |  |  |
| Kim K, 2007a | UNCLEAR | UNCLEAR | UNCLEAR | UNCLEAR | LOW | LOW |  |  |  |  |
| Kim K, 2007a | UNCLEAR | UNCLEAR | UNCLEAR | UNCLEAR | LOW | LOW |  |  |  |  |
| Berger WE, 2008 | UNCLEAR | UNCLEAR | UNCLEAR | HIGH | LOW | LOW |  |  |  |  |
| Lee CF, 2008 | UNCLEAR | UNCLEAR | UNCLEAR | UNCLEAR |  |  |  |  | LOW | LOW |
| Maspero JF, 2008 | UNCLEAR | UNCLEAR | UNCLEAR | LOW | UNCLEAR | HIGH |  |  |  |  |
| Tripathy I, 2008 | LOW | LOW | LOW | LOW | LOW | LOW |  |  |  |  |
| Li AM, 2009 | LOW | UNCLEAR | LOW | LOW | LOW | LOW |  |  |  |  |
| Weinstein S, 2009 | UNCLEAR | UNCLEAR | UNCLEAR | UNCLEAR | UNCLEAR | LOW |  |  |  |  |
| Baena-Cagnani CE, 2010 | UNCLEAR | UNCLEAR | UNCLEAR | UNCLEAR | UNCLEAR | LOW |  |  |  |  |
| Berman G, 2012 | UNCLEAR | UNCLEAR | LOW | LOW | LOW | LOW |  |  |  |  |
| Bernstein JA, 2014 | UNCLEAR | UNCLEAR | UNCLEAR | HIGH | UNCLEAR | LOW |  |  |  |  |
| Lee L, 2014 | UNCLEAR | UNCLEAR | UNCLEAR | LOW | LOW | HIGH |  |  |  |  |
| Berger WE, 2015 | UNCLEAR | UNCLEAR | UNCLEAR | LOW | LOW | LOW |  |  |  |  |
| NCT01451541 | UNCLEAR | UNCLEAR | UNCLEAR | LOW | LOW | LOW |  |  | LOW | LOW |
| Kim CK, 2024 | UNCLEAR | UNCLEAR | HIGH | HIGH | HIGH | LOW |  |  |  |  |
| **Children with seasonal allergic rhinitis** | | | | | | | | | | |
| Masi M, 1993 | UNCLEAR | UNCLEAR | UNCLEAR | UNCLEAR | LOW | LOW |  |  |  |  |
| Galant S, 1994 | UNCLEAR | UNCLEAR | UNCLEAR | LOW | UNCLEAR | LOW |  |  |  |  |
| Banov CH, 1996 | UNCLEAR | UNCLEAR | UNCLEAR | UNCLEAR | UNCLEAR | LOW |  |  |  |  |
| Schenkel EJ, 1997 | UNCLEAR | UNCLEAR | UNCLEAR | UNCLEAR | LOW | LOW | LOW | LOW |  |  |
| Meltzer EO, 1999 | UNCLEAR | UNCLEAR | LOW | LOW | LOW | LOW |  |  |  |  |
| de Blic J, 2005 | UNCLEAR | UNCLEAR | LOW | UNCLEAR |  |  |  |  | LOW | UNCLEAR |
| Berger WE, 2008 | UNCLEAR | UNCLEAR | UNCLEAR | LOW | LOW | UNCLEAR |  |  |  |  |
| Berger WE 2009 | UNCLEAR | UNCLEAR | LOW | LOW | LOW | LOW | LOW | LOW | LOW | HIGH |
| Meltzer E, 2009 | LOW | LOW | LOW | LOW | LOW | LOW | LOW | LOW |  |  |
| Storms WW, 2013 | UNCLEAR | UNCLEAR | UNCLEAR | LOW | LOW | LOW |  |  |  |  |
| NCT01458275 | UNCLEAR | UNCLEAR | UNCLEAR | LOW | LOW | LOW | LOW | LOW | LOW | LOW |
| Berger WE 2016 | LOW | LOW | LOW | LOW | LOW | LOW | LOW | LOW | LOW | LOW |
| Prenner B 2022 | LOW | LOW | LOW | LOW | LOW | LOW | LOW | LOW | LOW | LOW |

RQLQ=Rhinoconjunctivitis quality of life questionnaire; rTNSS=Reflective total nasal symptom score; rTOSS=Reflective total ocular symptom score

**Supplementary Table 6. Results of the direct comparisons between non-standard *versus* standard treatment doses of the same medication: Safety outcomes.** These results concern only direct evidence from head-to-head trials comparing, for a same medication, different doses *versus* the standard dose currently used in practice.

| **Medication** | **Dose comparison** | **Meta-analytical results - RR (95%CI) [*p*-value] [*I*^2^] [*N* studies; *N* participants]** | |
| --- | --- | --- | --- |
|  |  | **AE** | **Withdrawal due to AE** |
| Azelastine | 840 *vs* 560 μg | 1.15 (0.77;1.72) [0.498] [-] [1; 327] | 5.15 (0.25;106.4) [0.289] [-] [1; 327] |
| Azelastine | 1120 *vs* 560 μg | - | 2.92 (0.31;27.9) [0.353] [-] [1; 560] |
| Beclomethasone | 80 *vs* 320 μg | 1.03 (0.61;1.76) [0.903] [-] [1; 240] | 0.21 (0.01;4.26) [0.307] [-] [1; 240] |
| Beclomethasone | 160 *vs* 320 μg | 0.81 (0.46;1.44) [0.473] [-] [1; 245] | 0.60 (0.08;4.43) [0.613] [-] [1; 245] |
| Budesonide | 32 vs 256 μg | - | 3.80 (0.60;23.9) [0.359] [0%] [2; 357] |
| Budesonide | 64 vs 256 μg | - | 2.44 (0.36; 16.4) [0.797] [0%] [2; 352] |
| Budesonide | 128 vs 256 μg | 0.94 (0.70;1.26) [0.682] [-] [1; 363] | 1.25 (0.23; 6.70) [44%] [4; 937] |
| Budesonide | 400 vs 256 μg | - | 1.01 (0.09;11.0) [0.992] [-] [1; 238] |
| Ciclesonide | 25 *vs* 200 μg | 0.97 (0.85;1.11) [0.673] [0%] [3; 689] | 1.16 (0.33;4.08) [0.817] [0%] [2; 624] |
| Ciclesonide | 50 *vs* 200 μg | 1.22 (0.82;1.84) [0.323] [-] [1; 287] | 3.02 (0.12;73.5) [0.497] [-] [1; 287] |
| Ciclesonide | 100 *vs* 200 μg | 1.03 (0.91;1.17) [0.640] [0%] [4; 1099] | 1.73 (0.46;6.44) [0.415] [8%] [2; 619] |
| Ciclesonide | 160 *vs* 200 μg | 0.70 (0.46;1.08) [0.109] [-] [1; 108] | - |
| Ciclesonide | 320 *vs* 200 μg | 0.90 (0.61;1.35) [0.617] [-] [1; 99] | - |
| Ebastine | 20 *vs* 10 mg | 0.86 (0.61;1.22) [0.399] [-] [1; 214] | 1.34 (0.32;5.63) [0.692] [44%] [3; 758] |
| Fexofenadine | 120 *vs* 180 mg | 0.99 (0.77;1.27) [0.912] [-] [1; 570] | - |
| FF | 55 *vs* 110 μg | 0.96 (0.83;1.11) [0.574] [0%] [3; 992] | 2.70 (0.80;9.15) [0.111] [0%] [3; 992] |
| FF | 220 *vs* 110 μg | 0.93 (0.63;1.38) [0.722] [-] [1; 256] | 3.94 (0.45;34.7) [0.217] [-] [1; 256] |
| FF | 440 *vs* 110 μg | 0.82 (0.54;1.23) [0.338] [-] [1; 257] | 0.98 (0.06;15.5) [0.987] [-] [1; 257] |
| FP | 50 *vs* 200 μg | 1.23 (0.85;1.76) [0.275] [-] [1; 215] | - |
| FP | 100 *vs* 200 μg | 1.26 (0.84;1.90) [0.267] [-] [1; 165] | - |
| FP | 800 *vs* 200 μg | 1.30 (0.90;1.86) [0.160] [-] [1; 209] | - |
| Mometasone | 25 *vs* 200 μg | 1.23 (0.71;2.13) [0.469] [-] [1; 270] | 0.65 (0.11;3.81) [0.631] [-] [1; 270] |
| Mometasone | 100 *vs* 200 μg | 1.14 (0.82;1.59) [0.446] [0%] [2; 520] | 0.76 (0.28;2.08) [0.597] [0%] [2; 520] |
| Montelukast | 20 *vs* 10 mg | - | 5.28 (0.26;108.7) [0.281] [-] [1; 185] |
| Olopatadine | 2660 *vs* 5320 μg | 0.69 (0.39;1.22) [0.204] [-] [1; 318] | 1.65 (0.08;4.55) [0.627] [-] [1; 318] |
| OM | 2760 *vs* 5320 μg | 0.88 (0.45;1.69) [0.695] [-] [1; 315] | 0.99 (0.17;5.66) [0.994] [-] [1; 315] |
| Rupatadine | 20 *vs* 10 mg | 1.06 (0.72;1.57) [0.753] [-] [1; 598] | 1.03 (0.07;16.1) [0.984] [-] [1; 144] |
| Triamcinolone | 110 *vs* 220 μg | 0.70 (0.36;1.35) [0.286] [-] [1; 147] | - |

AE: Adverse events; CI: Confidence interval; FF: Fluticasone furoate; FP: Fluticasone propionate; OM: Olopatadine-mometasone; RR: Risk ratio

**Supplementary Table 7. Results of the direct comparisons between co-medication strategies and medications in monotherapy: Safety outcomes.** These results concern only direct evidence from head-to-head trials comparing, for a same medication, its use in co-medication *versus* in monotherapy in the standard dose currently used in practice.

| **Comparison** | **Meta-analytical results - RR (95%CI) [*p*-value] [*I*^2^] [*N* studies; *N* participants]** | |
| --- | --- | --- |
|  | **AE** | **Withdrawal due to AE** |
| Azelastine-fluticasone vs azelastine | 1.04 (0.73;1.48) [0.827] [50%] [5; 2149] | 1.19 (0.46;3.06) [0.723] [8%] [4; 2000] |
| Azelastine-fluticasone vs FP | 1.56 (1.07;2.26) [0.019] [45%] [4; 1998] | 1.75 (0.59;5.22) [0.314] [0%] [4; 2000] |
| Bilastine + montelukast vs bilastine | 1.96 (0.60;6.36) [0.263] [-] [1; 283] | 1.00 (0.04;24.4) [1.000] [-] [1; 283] |
| Bilastine + montelukast vs montelukast | 0.51 (0.22;1.16) [0.107] [-] [1; 279] | 0.32 (0.01;7.88) [0.488] [-] [1; 279] |
| Ciclesonide + levocetirizine vs ciclesonide | 1.00 (0.50;1.98) [0.991] [-] [1; 51] | - |
| Ciclesonide + levocetirizine vs levocetirizine | 0.68 (0.36;1.29) [0.239] [-] [1; 44] | - |
| FF + oxymetazoline vs FF | 0.50 (0.11; 2.35) [0.381] [-] [1; 32] | 3.00 (0.13; 69.0) [0.492] [-] [1; 32] |
| FF + oxymetazoline vs oxymetazoline | 0.94 (0.17; 6.67) [0.948] [-] [1; 33] | 3.00 (0.13; 69.0) [0.492] [-] [1; 33] |
| FP + loratadine vs FP | 0.85 (0.53;1.36) [0.489] [59%] [2; 611] | 0.13 (0.02; 1.05) [0.056] [0%] [2; 611] |
| FP + loratadine vs loratadine | 0.87 (0.66;1.16) [0.350] [0%] [2; 616] | 0.20 (0.02;1.72) [0.143] [0%] [2; 616] |
| Loratadine + montelukast vs loratadine | 0.88 (0.64;1.22) [0.435] [18%] [2; 943] | 0.79 (0.31;2.01) [0.623] [0%] [5; 1788] |
| Loratadine + montelukast vs montelukast | 1.01 (0.65;1.55) [0.976] [-] [1; 457] | 0.86 (0.21;3.51) [0.835] [0%] [4; 1240] |
| Mometasone + oxymetazoline vs mometasone | 0.78 (0.34;1.83) [0.575] [-] [1; 284] | 0.96 (0.10;9.11) [0.971] [-] [1; 284] |
| Mometasone + oxymetazoline vs oxymetazoline | 1.24 (0.48;3.24) [0.659] [-] [1; 285] | 0.97 (0.10;9.17) [0.976] [-] [1; 285] |
| Olopatadine-mometasone vs mometasone | 1.42 (1.08;1.88) [0.012] [0%] [3; 1660] | 0.55 (0.15;2.07) [0.377] [20%] [3; 1660] |
| Olopatadine-mometasone vs olopatadine | 1.08 (0.68;1.74) [0.734] [65%] [3; 1504] | 0.84 (0.25;2.78) [0.771] [0%] [2; 1504] |

AE: Adverse events; CI: Confidence interval; FF: Fluticasone furoate; FP: Fluticasone propionate; RR: Risk ratio

**Supplementary Table 8. Certainty of Evidence assessment for the comparisons on the total nasal symptom score**

| **Comparison** | **Direct CoE** | | | | | **Indirect CoE** | | | **NMA CoE** | | | **Best evidence** | **Final CoE** |
| --- | --- | --- | --- | --- | --- | --- | --- | --- | --- | --- | --- | --- | --- |
|  | **Risk of Bias** | **Inconsistency** | **Imprecision** | **Indirectness** | **Dissemination Bias** | **CoE first order loop** | **Intransitivity** | **Imprecision** | **Starting point for NMA evaluation** | **Incoherence** | **Imprecision** |  |  |
| Azelastine (double dose) vs azelastine + loratadine | Not serious | Not serious | Serious | Serious | Not serious | Moderate | Not serious | Serious | Moderate | Not serious | Serious | NMA | Low |
| Loratadine (double dose) vs azelastine + loratadine |  |  |  |  |  | Low | Not serious | Not serious | Low | - | Not serious | Indirect | Low |
| Azelastine (double dose) vs azelastine + fexofenadine | Not serious | Not serious | Serious | Serious | Not serious | Moderate | Not serious | Serious | Moderate | Not serious | Serious | NMA | Low |
| Fexofenadine (double dose) vs azelastine + fexofenadine |  |  |  |  |  | Moderate | Not serious | Serious | Moderate | - | Serious | Indirect | Low |
| Azelastine (double dose) vs azelastine-fluticasone | Not serious | Not serious | Not serious | Not serious | Not serious | High | Not serious | Not serious | High | Not serious | Not serious | NMA | High |
| FP (double dose) vs azelastine-fluticasone | Not serious | Not serious | Serious | Serious | Not serious | Low | Not serious | Not serious | Moderate | Not serious | Not serious | NMA | Moderate |
| Montelukast (double dose) vs loratadine + montelukast | Serious | Not serious | Serious | Not serious | Not serious | Moderate | Not serious | Not serious | Moderate | Not serious | Not serious | NMA | Moderate |
| Loratadine (double dose) vs loratadine + montelukast | Very serious | Serious | Not serious | Serious | Not serious | Low | Not serious | Not serious | Low | Not serious | Not serious | NMA | Low |
| FP (double dose) vs FP + loratadine | Very serious | Very serious | Serious | Serious | Not serious | Low | Not serious | Not serious | Low | Serious | Not serious | NMA | Low |
| Loratadine (double dose) vs FP + loratadine | Extremely serious | Serious | Serious | Serious | Not serious | Low | Not serious | Serious | Low | Not serious | Serious | NMA | Very low |
| Olopatadine (double dose) vs olopatadine-mometasone | Not serious | Not serious | Not serious | Serious | Not serious | Very low | Not serious | Serious | Moderate | Serious | Serious | Direct | Moderate |
| Mometasone (double dose) vs olopatadine-mometasone | Not serious | Not serious | Not serious | Serious | Not serious | Very low | Not serious | Not serious | Moderate | Serious | Not serious | Direct | Moderate |
| Montelukast (double dose) vs levocetirizine + montelukast | Very serious | Not serious | Serious | Very serious | Not serious |  |  |  | Very low | - | Extremely serious | NMA | Very low |
| Levocetirizine (double dose) vs levocetirizine + montelukast |  |  |  |  |  | Moderate | Not serious | Very serious | Moderate | - | Very serious | Indirect | Very low |
| Montelukast (double dose) vs bilastine + montelukast | Not serious | Not serious | Serious | Serious | Not serious | Low | Not serious | Not serious | Moderate | Serious | Not serious | Direct | Low |
| Bilastine (double dose) vs bilastine + montelukast | Not serious | Not serious | Not serious | Serious | Not serious | Low | Not serious | Not serious | Moderate | Serious | Not serious | Direct | Moderate |
| Budesonide (double dose) vs loratadine + budesonide |  |  |  |  |  | Very low | Not serious | Extremely serious | Very low | - | Extremely serious | Indirect | Very low |
| Loratadine (double dose) vs loratadine + budesonide | Very serious | Not serious | Extremely serious | Serious | Not serious | Very low | Not serious | Extremely serious | Very low | Not serious | Extremely serious | NMA | Very low |
| Montelukast (double dose) vs fexofenadine + montelukast |  |  |  |  |  | Moderate | Not serious | Extremely serious | Moderate | - | Extremely serious | Indirect | Very low |
| Fexofenadine (double dose) vs fexofenadine + montelukast | Not serious | Not serious | Extremely serious | Serious | Not serious |  |  |  | Moderate | - | Extremely serious | NMA | Very low |
| Ciclesonide (double dose) vs ciclesonide + levocetirizine | Very serious | Not serious | Very serious | Serious | Not serious | Very low | Not serious | Very serious | Very low | Serious | Very serious | NMA | Very low |
| Levocetirizine (double dose) vs ciclesonide + levocetirizine | Very serious | Not serious | Serious | Serious | Not serious | Very low | Not serious | Serious | Very low | Not serious | Serious | NMA | Very low |
| FP (double dose) vs FP + montelukast | Not serious | Not serious | Extremely serious | Very serious | Not serious |  |  |  | Low | - | Extremely serious | NMA | Very low |
| Montelukast (double dose) vs FP + montelukast |  |  |  |  |  | Moderate | Not serious | Extremely serious | Moderate | - | Extremely serious | Indirect | Very low |
| Azelastine (double dose) vs ciclesonide + azelastine |  |  |  |  |  | Very low | Not serious | Serious | Very low | - | Serious | Indirect | Very low |
| Ciclesonide (double dose) vs ciclesonide + azelastine | Not serious | Not serious | Not serious | Serious | Not serious |  |  |  | Moderate | - | Not serious | NMA | Moderate |
| Mometasone (double dose) vs mometasone + montelukast | Very serious | Not serious | Very serious | Serious | Not serious | Low | Not serious | Extremely serious | Low | Serious | Extremely serious | Indirect | Very low |
| Montelukast (double dose) vs mometasone + montelukast |  |  |  |  |  | Moderate | Not serious | Extremely serious | Moderate | - | Extremely serious | Indirect | Very low |

CoE: Certainty of Evidence; FP: Fluticasone propionate; NMA: Network meta-analysis

**Supplementary Table 9. Comparison between doubling the dose of the medication on each row to adding the medication of each column (sensitivity analysis: randomized controlled trials evaluating adult patients).** Results are expressed as mean differences (95% confidence intervals [CI]). Example interpretation of results: for the first non-empty cell in the first column, a double dose of ciclesonide is associated with a mean difference of -0.92 units (95%CI=-0.99;-0.85) of the Total Nasal Symptom Score compared to ciclesonide + azelastine.

| **A. Total Nasal Symptom Score** | | | | | | | | | | | | |
| --- | --- | --- | --- | --- | --- | --- | --- | --- | --- | --- | --- | --- |
|  | Azelastine | Olopatadine | Ciclesonide | | FP | | Mometasone | | Bilastine | Fexofenadine | Loratadine | Montelukast |
| Azelastine |  |  | **-0.38**  **(-0.64;-0.11)** | | **0.35**  **(0.28;0.41)** | |  | |  | **-0.45**  **(-0.67;-0.23)** | **-0.41**  **(-0.59;-0.23)** |  |
| Olopatadine |  |  |  | |  | | **-0.37**  **(-0.66;-0.09)** | |  |  |  |  |
| Ciclesonide | **-0.92**  **(-0.99;-0.85)** |  |  | |  | |  | |  |  |  |  |
| FP | **-0.52**  **(-0.55;-0.48)** |  |  | |  | |  | |  |  | 0.32  (-0.03;0.32) | **1.21**  **(0.49;1.94)** |
| Mometasone |  | **-0.56**  **(-0.57;-0.55)** |  | |  | |  | |  |  |  | -0.59  (-1.68;0.50) |
| Bilastine |  |  |  | |  | |  | |  |  |  | **-0.69**  **(-0.82;-0.56)** |
| Fexofenadine | 0.61  (-0.12;1.34) |  |  | |  | |  | |  |  |  |  |
| Loratadine | **-0.55**  **(-0.55;-0.54)** |  |  | | **1.07**  **(0.87;1.28)** | |  | |  |  |  | **0.11**  **(0.06;0.16)** |
| Montelukast |  |  |  | | **2.58**  **(2.04;3.11)** | | 0.82  (-0.23;1.87) | | -0.06  (-0.53;0.41) |  | **0.72**  **(0.63;0.81)** |  |
| **B. Total Ocular Symptom Score** | | | | | | | | | | | | |
|  | Azelastine | Olopatadine | FP | | | Mometasone | | | Bilastine | | | Montelukast |
| Azelastine |  |  | -0.09  (-0.23;0.04) | | |  | | |  | | |  |
| Olopatadine |  |  |  | | | -0.29  (-0.60;0.02) | | |  | | |  |
| FP | **-0.13**  **(-0.23;-0.03)** |  |  | | |  | | |  | | |  |
| Mometasone |  | -0.12  (-0.36;0.12) |  | | |  | | |  | | |  |
| Bilastine |  |  |  | | |  | | |  | | | **-0.65**  **(-0.85;-0.46)** |
| Montelukast |  |  |  | | |  | | | **-0.20**  **(-0.39;-0.02)** | | |  |
| **C. Rhinoconjunctivitis Quality of Life Questionnaire** | | | | | | | | | | | | |
|  | Azelastine | Olopatadine | FF | FP | | Mometasone | | Oxymetazoline | Loratadine | | | Montelukast |
| Azelastine |  |  |  | **0.27**  **(0.23;0.31)** | |  | |  |  | | |  |
| Olopatadine |  |  |  |  | | -0.11  (-0.28;0.06) | |  |  | | |  |
| FF |  |  |  |  | |  | | -0.49  (-1.91;0.92) |  | | |  |
| FP | **-0.51**  **(-0.68;-0.35)** |  |  |  | |  | |  | **-0.20**  **(-0.25;-0.14)** | | |  |
| Mometasone |  | **-0.27**  **(-0.38;-0.15)** |  |  | |  | | **-0.33**  **(-0.62;-0.04)** |  | | |  |
| Oxymetazoline |  |  | -0.26  (-0.71;0.19) |  | | 0.19  (-0.36;0.73) | |  |  | | |  |
| Loratadine |  |  |  | **0.61**  **(0.51;0.71)** | |  | |  |  | | | -0.01  (-0.07;0.06) |
| Montelukast |  |  |  |  | |  | |  | **0.13**  **(0.10;0.17)** | | |  |

FF=Fluticasone furoate; FP=Fluticasone propionate

**Supplementary Table 10. Comparison between doubling the dose of the medication on each row to adding the medication of each column (sensitivity analysis: randomized controlled trials differentiating nasal and oral placebo).** Results are expressed as mean differences (95% confidence intervals [CI]). Example interpretation of results: for the first non-empty cell in the first column, a double dose of ciclesonide is associated with a mean difference of -0.16 units (95%CI=-0.20;-0.12) of the Total Nasal Symptom Score compared to ciclesonide + azelastine.

| **A. Total Nasal Symptom Score** | | | | | | | | | | | |
| --- | --- | --- | --- | --- | --- | --- | --- | --- | --- | --- | --- |
|  | Azelastine | Olopatadine | Budesonide | Ciclesonide | FP | Mometasone | Bilastine | Fexofenadine | Levocetirizine | Loratadine | Montelukast |
| Azelastine |  |  |  | -0.14  (-0.33;0.05) | **0.41**  **(0.38;0.45)** |  |  | **-0.22**  **(-0.39;-0.05)** |  | -0.09  (-0.22;0.05) |  |
| Olopatadine |  |  |  |  |  | 0.14  (-0.12;0.40) |  |  |  |  |  |
| Budesonide |  |  |  |  |  |  |  |  |  | 0.11  (-0.80;1.02) |  |
| Ciclesonide | **-0.16**  **(-0.20;-0.12)** |  |  |  |  |  |  |  | 0.56  (-0.30;1.42) |  |  |
| FP | **-0.33**  **(-0.34;-0.33)** |  |  |  |  |  |  |  |  | **0.59**  **(0.33;0.86)** | **1.67**  **(1.11;2.22)** |
| Mometasone |  | **-0.12**  **(-0.14;-0.10)** |  |  |  |  |  |  |  |  | 0.01  (-1.03;1.04) |
| Bilastine |  |  |  |  |  |  |  |  |  |  | **-0.69**  **(-0.79;-0.58)** |
| Fexofenadine | **1.16**  **(0.58;1.75)** |  |  |  |  |  |  |  |  |  | **1.75**  **(0.38;3.12)** |
| Levocetirizine |  |  |  | **1.30**  **(0.78;1.82)** |  |  |  |  |  |  | **1.56**  **(0.18;2.94)** |
| Loratadine | **-0.43**  **(-0.43;-0.42)** |  | 0.29  (-0.77;1.35) |  | **1.04**  **(0.85;1.22)** |  |  |  |  |  | **0.18**  **(0.14;0.22)** |
| Montelukast |  |  |  |  | **2.65**  **(2.21;3.09)** | 0.69  (-0.36;1.74) | 0.09  (-0.30;0.48) | 0.56  (-1.41;2.53) | 1.03  (-0.77;2.83) | **0.71**  **(0.64;0.78)** |  |
| **B. Total Ocular Symptom Score** | | | | | | | | | | | |
|  | Azelastine | Olopatadine | FP | | Mometasone | | Bilastine | | | | Montelukast |
| Azelastine |  |  | 0.08  (-0.06;0.22) | |  | |  | | | |  |
| Olopatadine |  |  |  | | -0.17  (-0.49;0.14) | |  | | | |  |
| FP | **-0.08**  **(-0.24;0.07)** |  |  | |  | |  | | | |  |
| Mometasone |  | -0.01  (-0.27;0.26) |  | |  | |  | | | |  |
| Bilastine |  |  |  | |  | |  | | | | **-0.65**  **(-0.83;-0.48)** |
| Montelukast |  |  |  | |  | | **-0.17**  **(-0.35;0)** | | | |  |
| **C. Rhinoconjunctivitis Quality of Life Questionnaire** | | | | | | | | | | | |
|  | Azelastine | Olopatadine | FF | FP | Mometasone | Oxymetazoline | Loratadine | | | | Montelukast |
| Azelastine |  |  |  | **0.28 (0.26;0.29)** |  |  |  | | | |  |
| Olopatadine |  |  |  |  | 0.07  (-0.15;0.30) |  |  | | | |  |
| FF |  |  |  |  |  | -0.50  (-1.90;0.89) |  | | | |  |
| FP | **-0.40**  **(-0.60;-0.19)** |  |  |  |  |  | **-0.03**  **(-0.05;-0.01)** | | | |  |
| Mometasone |  | -0.09  (-0.26;0.08) |  |  |  | -0.15  (-0.37;0.06) |  | | | |  |
| Oxymetazoline |  |  | -0.06  (-0.47;0.34) |  | 0.35  (-0.22;0.91) |  |  | | | |  |
| Loratadine |  |  |  | **0.53**  **(0.42;0.64)** |  |  |  | | | | 0.02  (-0.04;0.09) |
| Montelukast |  |  |  |  |  |  | **0.13**  **(0.09;0.16)** | | | |  |

FF=Fluticasone furoate; FP=Fluticasone propionate

**Supplementary Table 11. Certainty of Evidence assessment for the comparisons on the total ocular symptom score**

| **Comparison** | **Direct CoE** | | | | | **Indirect CoE** | | | **NMA CoE** | | | **Best evidence** | **Final CoE** |
| --- | --- | --- | --- | --- | --- | --- | --- | --- | --- | --- | --- | --- | --- |
|  | **Risk of Bias** | **Inconsistency** | **Imprecision** | **Indirectness** | **Dissemination Bias** | **CoE first order loop** | **Intransitivity** | **Imprecision** | **Starting point for NMA evaluation** | **Incoherence** | **Imprecision** |  |  |
| Azelastine (double dose) vs azelastine + fluticasone | Not serious | Not serious | Serious | Serious | Not serious | High | Not serious | Not serious | High | Not serious | Not serious | NMA | High |
| FP (double dose) vs azelastine + fluticasone | Not serious | Not serious | Not serious | Serious | Not serious | Moderate | Not serious | Not serious | Moderate | Not serious | Not serious | NMA | Moderate |
| Mometasone (double dose) vs olopatadine + mometasone | Not serious | Not serious | Serious | Serious | Not serious | Very low | Not serious | Serious | Moderate | Not serious | Serious | NMA | Low |
| Olopatadine (double dose) vs olopatadine + mometasone | Not serious | Not serious | Serious | Serious | Not serious | Low | Not serious | Serious | Moderate | Not serious | Serious | NMA | Low |
| Montelukast (double dose) vs bilastine + montelukast | Not serious | Not serious | Serious | Serious | Not serious | Moderate | Not serious | Not serious | Moderate | Serious | Not serious | Indirect | Moderate |
| Bilastine (double dose) vs bilastine + montelukast | Not serious | Not serious | Not serious | Serious | Not serious | Moderate | Not serious | Not serious | Moderate | Not serious | Not serious | NMA | Moderate |

CoE: Certainty of Evidence; NMA: Network meta-analysis

**Supplementary Table 12. Certainty of Evidence assessment for the comparisons on the rhinoconjunctivitis quality-of-life questionnaire**

| **Comparison** | **Direct CoE** | | | | | **Indirect CoE** | | | **NMA CoE** | | | **Best evidence** | **Final CoE** |
| --- | --- | --- | --- | --- | --- | --- | --- | --- | --- | --- | --- | --- | --- |
|  | **Risk of Bias** | **Inconsistency** | **Imprecision** | **Indirectness** | **Dissemination Bias** | **CoE first order loop** | **Intransitivity** | **Imprecision** | **Starting point for NMA evaluation** | **Incoherence** | **Imprecision** |  |  |
| Azelastine (double dose) vs azelastine-fluticasone | Not serious | Not serious | Not serious | Not serious | Not serious | High | Not serious | Not serious | High | Not serious | Not serious | NMA | High |
| FP (double dose) vs azelastine-fluticasone | Not serious | Not serious | Serious | Not serious | Not serious | Moderate | Not serious | Serious | High | Not serious | Serious | NMA | Moderate |
| FP (double dose) vs FP + loratadine | Very serious | Not serious | Very serious | Serious | Not serious | Low | Not serious | Not serious | Low | Serious | Not serious | Indirect | Low |
| Loratadine (double dose) vs FP + loratadine | Very serious | Not serious | Serious | Serious | Not serious | Low | Not serious | Not serious | Low | Serious | Not serious | Indirect | Low |
| Mometasone (double dose) vs mometasone + oxymetazoline | Very serious | Not serious | Serious | Serious | Not serious | Low | Not serious | Not serious | Low | Not serious | Not serious | NMA | Low |
| Oxymetazoline (double dose) vs mometasone + oxymetazoline | Very serious | Not serious | Very serious | Serious | Not serious | Very low | Not serious | Serious | Very low | Not serious | Serious | NMA | Very low |
| Olopatadine (double dose) vs olopatadine-mometasone | Not serious | Not serious | Serious | Serious | Not serious | Very low | Not serious | Serious | Moderate | Not serious | Serious | NMA | Low |
| Mometasone (double dose) vs olopatadine-mometasone | Not serious | Not serious | Serious | Serious | Not serious | Very low | Not serious | Serious | Moderate | Not serious | Serious | NMA | Low |
| Loratadine (double dose) vs loratadine + montelukast | Not serious | Very serious | Serious | Serious | Not serious | Low | Not serious | Not serious | Low | Not serious | Not serious | NMA | Low |
| Montelukast (double dose) vs loratadine + montelukast | Not serious | Not serious | Serious | Not serious | Not serious | Low | Not serious | Not serious | High | Not serious | Not serious | NMA | High |
| FF (double dose) vs FF + oxymetazoline | Not serious | Not serious | Extremely serious | Serious | Not serious | Low | Not serious | Very serious | Moderate | Not serious | Very serious | NMA | Very low |
| Oxymetazoline (double dose) vs FF + oxymetazoline | Not serious | Not serious | Extremely serious | Serious | Not serious | Very low | Not serious | Serious | Moderate | Not serious | Serious | NMA | Low |

CoE: Certainty of Evidence; FF: Fluticasone furoate; FP: Fluticasone propionate; NMA: Network meta-analysis

**Supplementary Table 13. Comparison between doubling the dose of the medication on each row to adding the medication of each column on the occurrence of adverse events (main and sensitivity analyses).** Results are expressed as risk ratios (95% confidence intervals [CI]). Example interpretation of results: for the first non-empty cell in the first column, a double dose of ciclesonide is associated with 1.46 times (95%CI=1.37;1.56) increase in the risk of adverse events compared to ciclesonide + azelastine.

| **A. Main analysis: Patients of all ages and with seasonal or perennial allergic rhinitis** | | | | | | | | | | | | | |
| --- | --- | --- | --- | --- | --- | --- | --- | --- | --- | --- | --- | --- | --- |
|  | Azelastine | Olopatadine | Ciclesonide | FF | | FP | | Mometasone | Oxymetazoline | Bilastine | Levocetirizine | Loratadine | Montelukast |
| Azelastine |  |  | **1.91 (1.68;2.17)** |  | | **0.91 (0.86;0.95)** | |  |  |  |  |  |  |
| Olopatadine |  |  |  |  | |  | | **1.38 (1.03;1.85)** |  |  |  |  |  |
| Ciclesonide | **1.46 (1.37;1.56)** |  |  |  | |  | |  |  |  | 1.27 (0.83;1.94) |  |  |
| FF |  |  |  |  | |  | |  | 1.66 (0.47;5.92) |  |  |  |  |
| FP | **0.65 (0.58;0.72)** |  |  |  | |  | |  |  |  |  | 1.07 (0.88;1.31) |  |
| Mometasone |  | **0.74 (0.73;0.76)** |  |  | |  | |  | 1.12 (0.64;1.97) |  |  |  |  |
| Oxymetazoline |  |  |  | **0.63 (0.50;0.78)** | |  | | 0.48 (0.20;1.13) |  |  |  |  |  |
| Bilastine |  |  |  |  | |  | |  |  |  |  |  | **0.89 (0.85;0.93)** |
| Levocetirizine |  |  | 1.26 (0.94;1.69) |  | |  | |  |  |  |  |  | 0.97 (0.39;2.42) |
| Loratadine |  |  |  |  | | **1.13 (1.03;1.24)** | |  |  |  |  |  | **1.11 (1.07;1.16)** |
| Montelukast |  |  |  |  | |  | |  |  | **2.19 (1.39;3.44)** | 0.97 (0.39;2.40) | **1.13 (1.01;1.26)** |  |
| **B. Sensitivity analysis: Seasonal allergic rhinitis** | | | | | | | | | | | | | |
|  | Azelastine | Olopatadine | Ciclesonide | | FP | | Mometasone | | Oxymetazoline | Bilastine | Levocetirizine | Loratadine | Montelukast |
| Azelastine |  |  | **1.91**  **(1.68;2.17)** | | **1.39**  **(1.25;1.56)** | |  | |  |  |  |  |  |
| Olopatadine |  |  |  | |  | | **1.32**  **(1.02;1.70)** | |  |  |  |  |  |
| Ciclesonide | **1.46 (1.37;1.56)** |  |  | |  | |  | |  |  | 1.10 (0.83;1.44) |  |  |
| FP | **0.62 (0.56;0.69)** |  |  | |  | |  | |  |  |  | 1.14 (0.95;1.37) |  |
| Mometasone |  | **0.62 (0.62;0.62)** |  | |  | |  | | 1.05 (0.64;1.71) |  |  |  |  |
| Oxymetazoline |  |  |  | |  | | 0.51  (0.17;1.56) | |  |  |  |  |  |
| Bilastine |  |  |  | |  | |  | |  |  |  |  | **0.66 (0.59;0.73)** |
| Levocetirizine |  |  | **1.28**  **(1.08;1.52)** | |  | |  | |  |  |  |  | **0.36 (0.13;1.00)** |
| Loratadine |  |  |  | | **1.17**  **(1.08;1.26)** | |  | |  |  |  |  | **1.17 (1.14;1.20)** |
| Montelukast |  |  |  | |  | |  | |  | **1.68 (1.02;2.76)** | 0.39 (0.12;1.24) | **1.18 (1.06;1.31)** |  |
| **C. Sensitivity analysis: Adult patients** | | | | | | | | | | | | | |
|  | Azelastine | Olopatadine | Ciclesonide | FF | | FP | | Mometasone | Oxymetazoline | Bilastine | Levocetirizine | Loratadine | Montelukast |
| Azelastine |  |  | **1.95 (1.72;2.22)** |  | | **0.91 (0.86;0.97)** | |  |  |  |  |  |  |
| Olopatadine |  |  |  |  | |  | | **1.40 (1.02;1.91)** |  |  |  |  |  |
| Ciclesonide | **1.55 (1.53;1.56)** |  |  |  | |  | |  |  |  | 1.27 (0.83;1.94) |  |  |
| FF |  |  |  |  | |  | |  | 1.65 (0.47;5.79) |  |  |  |  |
| FP | **0.62 (0.55;0.70)** |  |  |  | |  | |  |  |  |  | 1.07 (0.87;1.32) |  |
| Mometasone |  | **0.76 (0.74;0.77)** |  |  | |  | |  | 1.14 (0.65;1.99) |  |  |  |  |
| Oxymetazoline |  |  |  | **0.63 (0.51;0.80)** | |  | | 0.48 (0.20;1.13) |  |  |  |  |  |
| Bilastine |  |  |  |  | |  | |  |  |  |  |  | **0.66 (0.62;0.70)** |
| Levocetirizine |  |  | 1.26 (0.94;1.69) |  | |  | |  |  |  |  |  | **0.32 (0.11;0.92)** |
| Loratadine |  |  |  |  | | **1.13 (1.03;1.24)** | |  |  |  |  |  | **1.11 (1.07;1.16)** |
| Montelukast |  |  |  |  | |  | |  |  | 1.61 (0.99;2.63) | 0.37 (0.12;1.19) | **1.13 (1.00;1.27)** |  |
| **D. Sensitivity analysis: Nasal and oral placebo assessed separately** | | | | | | | | | | | | | |
|  | Azelastine | Olopatadine | Ciclesonide | FF | | FP | | Mometasone | Oxymetazoline | Bilastine | Levocetirizine | Loratadine | Montelukast |
| Azelastine |  |  | **1.83 (1.62;2.07)** |  | | **0.88 (0.84;0.93)** | |  |  |  |  |  |  |
| Olopatadine |  |  |  |  | |  | | **1.35 (1.02;1.80)** |  |  |  |  |  |
| Ciclesonide | **1.43 (1.37;1.49)** |  |  |  | |  | |  |  |  | 1.24 (0.84;1.85) |  |  |
| FF |  |  |  |  | |  | |  | 1.65 (0.47;5.78) |  |  |  |  |
| FP | **0.64 (0.59;0.69)** |  |  |  | |  | |  |  |  |  | 1.05 (0.89;1.24) |  |
| Mometasone |  | **0.74 (0.74;0.74)** |  |  | |  | |  | 1.10 (0.65;1.88) |  |  |  |  |
| Oxymetazoline |  |  |  | **0.61 (0.50;0.76)** | |  | | 0.47 (0.20;1.11) |  |  |  |  |  |
| Bilastine |  |  |  |  | |  | |  |  |  |  |  | 0.93 (0.85;1.02) |
| Levocetirizine |  |  | 1.25 (0.92;1.71) |  | |  | |  |  |  |  |  | 0.96 (0.39;2.40) |
| Loratadine |  |  |  |  | | **1.12 (1.03;1.22)** | |  |  |  |  |  | **1.10 (1.06;1.13)** |
| Montelukast |  |  |  |  | |  | |  |  | **2.16 (1.36;3.44)** | 0.96 (0.39;2.40) | **1.12 (1.02;1.24)** |  |

FF=Fluticasone furoate; FP=Fluticasone propionate

**Supplementary Table 14. Certainty of Evidence assessment for the comparisons on adverse events**

| **Comparison** | **Direct CoE** | | | | | **Indirect CoE** | | | **NMA CoE** | | | **Best evidence** | **Final CoE** |
| --- | --- | --- | --- | --- | --- | --- | --- | --- | --- | --- | --- | --- | --- |
|  | **Risk of Bias** | **Inconsistency** | **Imprecision** | **Indirectness** | **Dissemination Bias** | **CoE first order loop** | **Intransitivity** | **Imprecision** | **Starting point for NMA evaluation** | **Incoherence** | **Imprecision** |  |  |
| Azelastine (double dose) vs azelastine-fluticasone | Not serious | Not serious | Serious | Serious | Not serious | Low | Not serious | Not serious | Moderate | Serious | Not serious | NMA | Moderate |
| FP (double dose) vs azelastine-fluticasone | Not serious | Not serious | Not serious | Serious | Not serious | Low | Not serious | Not serious | Moderate | Not serious | Not serious | NMA | Moderate |
| FF (double dose) vs FF + oxymetazoline | Not serious | Not serious | Extremely serious | Serious | Not serious | Moderate | Not serious | Extremely serious | Moderate | Not serious | Extremely serious | NMA | Very low |
| Oxymetazoline (double dose) vs FF + oxymetazoline | Not serious | Not serious | Serious | Serious | Not serious | Moderate | Not serious | Not serious | Moderate | Not serious | Not serious | NMA | Moderate |
| FP (double dose) vs FP + loratadine | Serious | Not serious | Serious | Serious | Not serious | Low | Not serious | Not serious | Low | Not serious | Not serious | NMA | Low |
| Loratadine (double dose) vs FP + loratadine | Not serious | Serious | Serious | Serious | Not serious | Low | Not serious | Not serious | Low | Not serious | Not serious | NMA | Low |
| Loratadine (double dose) vs loratadine + montelukast | Not serious | Serious | Serious | Serious | Not serious | Moderate | Not serious | Not serious | Moderate | Not serious | Not serious | NMA | Moderate |
| Montelukast (double dose) vs loratadine + montelukast | Not serious | Not serious | Not serious | Serious | Not serious | Low | Not serious | Not serious | Moderate | Not serious | Not serious | NMA | Moderate |
| Mometasone (double dose) vs mometasone + oxymetazoline | Very serious | Not serious | Not serious | Serious | Not serious | Low | Not serious | Not serious | Low | Not serious | Not serious | NMA | Low |
| Oxymetazoline (double dose) vs mometasone + oxymetazoline | Very serious | Not serious | Not serious | Serious | Not serious | Low | Not serious | Not serious | Low | Not serious | Not serious | NMA | Low |
| Mometasone (double dose) vs olopatadine-mometasone | Not serious | Not serious | Not serious | Serious | Not serious | Moderate | Not serious | Not serious | Moderate | Not serious | Not serious | NMA | Moderate |
| Olopatadine (double dose) vs olopatadine-mometasone | Not serious | Not serious | Not serious | Serious | Not serious | Moderate | Not serious | Not serious | Moderate | Not serious | Not serious | NMA | Moderate |
| Montelukast (double dose) vs levocetirizine + montelukast | Very serious | Not serious | Extremely serious | Serious | Not serious |  | Not serious | Serious | Low | Serious | Serious | NMA | Very low |
| Levocetirizine (double dose) vs levocetirizine + montelukast |  |  |  |  |  |  |  |  | Very low | - | Extremely serious | NMA | Very low |
| Bilastine (double dose) vs bilastine + montelukast | Not serious | Not serious | Not serious | Serious | Not serious | Moderate | Not serious | Not serious | Moderate | Not serious | Not serious | NMA | Moderate |
| Montelukast (double dose) vs bilastine + montelukast | Not serious | Not serious | Very serious | Serious | Not serious | Moderate | Not serious | Serious | Moderate | Serious | Serious | Indirect | Low |
| Ciclesonide (double dose) vs ciclesonide + levocetirizine | Very serious | Not serious | Not serious | Serious | Not serious | Very low | Not serious | Very serious | Very low | Not serious | Very serious | NMA | Very low |
| Levocetirizine (double dose) vs ciclesonide + levocetirizine | Very serious | Not serious | Extremely serious | Serious | Not serious | Very low | Not serious | Extremely serious | Very low | Serious | Extremely serious | Indirect | Very low |
| Ciclesonide (double dose) vs ciclesonide + azelastine | Very serious | Not serious | Not serious | Serious | Not serious |  |  |  | Very low | - | Not serious | Direct | Very low |
| Azelastine (double dose) vs ciclesonide + azelastine |  |  |  |  |  |  |  |  | Very low | - | Not serious | NMA | Very low |

CoE: Certainty of Evidence; FF: Fluticasone furoate; FP: Fluticasone propionate; NMA: Network meta-analysis

**Supplementary Table 15. Comparison between doubling the dose of the medication on each row to adding the medication of each column on the occurrence of withdrawal due to adverse events (main and sensitivity analyses).** Results are expressed as risk ratios (95% confidence intervals [CI]). Example interpretation of results: for the first non-empty cell in the first column, a double dose of ciclesonide is associated with 1.20 times (95%CI=0.72;2.02) increase in the risk of adverse events compared to ciclesonide + azelastine.

| **A. Main analysis: Patients of all ages and with seasonal or perennial allergic rhinitis** | | | | | | | | | | | |
| --- | --- | --- | --- | --- | --- | --- | --- | --- | --- | --- | --- |
|  | Azelastine | Olopatadine | Ciclesonide | FF | FP | Mometasone | Oxymetazoline | Bilastine | Fexofenadine | Loratadine | Montelukast |
| Azelastine |  |  | **2.06 (1.28;3.34)** |  | 0.90 (0.59;1.36) |  |  |  | 2.02 (0.83;4.92) | 2.29 (0.82;6.42) |  |
| Olopatadine |  |  |  |  |  | 1.73 (0.85;3.50) |  |  |  |  |  |
| Ciclesonide | 1.20 (0.72;2.02) |  |  |  |  |  |  |  |  |  |  |
| FF |  |  |  |  |  |  | 0.24 (0.03;1.87) |  |  |  |  |
| FP | **0.60 (0.49;0.73)** |  |  |  |  |  |  |  |  | **3.87 (1.02;14.72)** |  |
| Mometasone |  | **1.19 (1.03;1.37)** |  |  |  |  | 1.18 (0.28;4.89) |  |  |  |  |
| Oxymetazoline |  |  |  | **0.48 (0.26;0.87)** |  | 1.37 (0.43;4.40) |  |  |  |  |  |
| Bilastine |  |  |  |  |  |  |  |  |  |  | 0.59 (0.27;1.29) |
| Fexofenadine | 2.04 (0.48;8.75) |  |  |  |  |  |  |  |  |  |  |
| Loratadine | **1.96 (1.01;3.81)** |  |  |  | **4.98 (1.52;16.28)** |  |  |  |  |  | **2.34 (2.15;2.55)** |
| Montelukast |  |  |  |  |  |  |  | 2.96 (0.32;27.30) |  | **2.81 (2.20;3.61)** |  |
| **B. Sensitivity analysis: Seasonal allergic rhinitis** | | | | | | | | | | | |
|  | Azelastine | Olopatadine | FP | | Mometasone | | Oxymetazoline | Bilastine | Fexofenadine | Loratadine | Montelukast |
| Azelastine |  |  | 0.80  (0.62;1.04) | |  | |  |  | 1.82 (0.89;3.69) | 2.01 (0.88;4.57) |  |
| Olopatadine |  |  |  | | 2.03  (0.83;4.93) | |  |  |  |  |  |
| FP | **0.54 (0.47;0.62)** |  |  | |  | |  |  |  | **3.62 (1.00;13.05)** |  |
| Mometasone |  | **1.35 (1.11;1.64)** |  | |  | | 1.05 (0.23;4.77) |  |  |  |  |
| Oxymetazoline |  |  |  | | 1.72  (0.16;18.91) | |  |  |  |  |  |
| Bilastine |  |  |  | |  | |  |  |  |  | 0.56 (0.20;1.59) |
| Fexofenadine | 1.93 (0.30;12.53) |  |  | |  | |  |  |  |  |  |
| Loratadine | 1.55 (0.93;2.59) |  | **4.15**  **(1.38;12.46)** | |  | |  |  |  |  | **1.63 (1.39;1.91)** |
| Montelukast |  |  |  | |  | |  | 2.68 (0.29;24.67) |  | **2.09 (1.43;3.07)** |  |
| **C. Sensitivity analysis: Adult patients** | | | | | | | | | | | |
|  | Azelastine | Olopatadine | Ciclesonide | FF | FP | Mometasone | Oxymetazoline | Bilastine | Fexofenadine | Loratadine | Montelukast |
| Azelastine |  |  | **2.16 (1.29;3.61)** |  | 0.92 (0.58;1.47) |  |  |  | 2.14 (0.89;5.15) | 2.43 (0.88;6.70) |  |
| Olopatadine |  |  |  |  |  | 1.66 (0.77;3.60) |  |  |  |  |  |
| Ciclesonide | 1.31 (0.60;2.84) |  |  |  |  |  |  |  |  |  |  |
| FF |  |  |  |  |  |  | 0.25 (0.03;1.89) |  |  |  |  |
| FP | **0.57 (0.44;0.74)** |  |  |  |  |  |  |  |  | **3.89 (1.02;14.86)** |  |
| Mometasone |  | **1.31 (1.17;1.47)** |  |  |  |  | 1.20 (0.29;4.91) |  |  |  |  |
| Oxymetazoline |  |  |  | **0.48 (0.26;0.91)** |  | 1.39 (0.43;4.56) |  |  |  |  |  |
| Bilastine |  |  |  |  |  |  |  |  |  |  | 0.59 (0.27;1.33) |
| Fexofenadine | 2.10 (0.47;9.42) |  |  |  |  |  |  |  |  |  |  |
| Loratadine | **1.92 (1.00;3.70)** |  |  |  | **4.97 (1.53;16.18)** |  |  |  |  |  | **2.27 (2.09;2.47)** |
| Montelukast |  |  |  |  |  |  |  | 2.92 (0.32;27.11) |  | **2.70 (2.10;3.48)** |  |
| **D. Sensitivity analysis: Nasal and oral placebo assessed separately** | | | | | | | | | | | |
|  | Azelastine | Olopatadine | Ciclesonide | FF | FP | Mometasone | Oxymetazoline | Bilastine | Fexofenadine | Loratadine | Montelukast |
| Azelastine |  |  | **2.21 (1.59;3.07)** |  | 0.92 (0.64;1.31) |  |  |  | 2.27 (1.00;5.16) | **2.67 (1.01;7.07)** |  |
| Olopatadine |  |  |  |  |  | **1.79 (1.26;2.54)** |  |  |  |  |  |
| Ciclesonide | **2.16 (1.13;4.13)** |  |  |  |  |  |  |  |  |  |  |
| FF |  |  |  |  |  |  | 0.28 (0.04;1.90) |  |  |  |  |
| FP | **0.78 (0.75;0.81)** |  |  |  |  |  |  |  |  | **5.03 (1.60;15.80)** |  |
| Mometasone |  | **2.01 (1.97;2.05)** |  |  |  |  | 1.45 (0.44;4.73) |  |  |  |  |
| Oxymetazoline |  |  |  | **0.54 (0.35;0.84)** |  | 1.85 (0.63;5.45) |  |  |  |  |  |
| Bilastine |  |  |  |  |  |  |  |  |  |  | 0.58 (0.31;1.08) |
| Fexofenadine | 2.73 (0.77;9.63) |  |  |  |  |  |  |  |  |  |  |
| Loratadine | **2.37 (1.19;4.73)** |  |  |  | **5.23 (1.59;17.18)** |  |  |  |  |  | **3.77 (3.22;4.41)** |
| Montelukast |  |  |  |  |  |  |  | 3.78 (0.42;34.32) |  | **4.80 (3.71;6.20)** |  |

FF=Fluticasone furoate; FP=Fluticasone propionate

**Supplementary Table 16. Certainty of Evidence assessment for the comparisons on withdrawals due to adverse events**

| **Comparison** | **Direct CoE** | | | | | **Indirect CoE** | | | **NMA CoE** | | | **Best evidence** | **Final CoE** |
| --- | --- | --- | --- | --- | --- | --- | --- | --- | --- | --- | --- | --- | --- |
|  | **Risk of Bias** | **Inconsistency** | **Imprecision** | **Indirectness** | **Dissemination Bias** | **CoE first order loop** | **Intransitivity** | **Imprecision** | **Starting point for NMA evaluation** | **Incoherence** | **Imprecision** |  |  |
| Azelastine (double dose) vs azelastine-fluticasone | Not serious | Not serious | Not serious | Serious | Not serious | High | Not serious | Not serious | High | Not serious | Not serious | NMA | High |
| FP (double dose) vs azelastine-fluticasone | Not serious | Not serious | Not serious | Serious | Not serious | Moderate | Not serious | Not serious | Moderate | Not serious | Not serious | NMA | Moderate |
| Azelastine (double dose) vs azelastine + fexofenadine | Not serious | Not serious | Not serious | Serious | Not serious | Moderate | Not serious | Not serious | Moderate | Not serious | Not serious | NMA | Moderate |
| Fexofenadine (double dose) vs azelastine + fexofenadine |  |  |  |  |  |  |  |  | Very low | - | Not serious | NMA | Very low |
| Azelastine (double dose) vs azelastine + loratadine | Not serious | Not serious | Not serious | Serious | Not serious | Moderate | Not serious | Not serious | Moderate | Not serious | Not serious | NMA | Moderate |
| Loratadine (double dose) vs azelastine + loratadine |  |  |  |  |  |  |  |  | Very low | - | Not serious | NMA | Very low |
| Bilastine (double dose) vs bilastine + montelukast | Not serious | Not serious | Not serious | Serious | Not serious | Moderate | Not serious | Not serious | Moderate | Not serious | Not serious | NMA | Moderate |
| Montelukast (double dose) vs bilastine + montelukast | Not serious | Not serious | Very serious | Serious | Not serious | Moderate | Not serious | Very serious | Moderate | Not serious | Very serious | NMA | Very low |
| Azelastine (double dose) vs ciclesonide + azelastine |  |  |  |  |  |  |  |  | Very low | - | Not serious | NMA | Very low |
| Ciclesonide (double dose) vs ciclesonide + azelastine | Very serious | Not serious | Not serious | Serious | Not serious |  |  |  | Very low | - | Not serious | Direct | Very low |
| FF (double dose) vs FF + oxymetazoline | Not serious | Not serious | Not serious | Serious | Not serious | High | Not serious | Not serious | High | Not serious | Not serious | NMA | High |
| Oxymetazoline (double dose) vs FF + oxymetazoline | Not serious | Not serious | Not serious | Serious | Not serious | Very low | Not serious | Not serious | Moderate | Not serious | Not serious | NMA | Moderate |
| FP (double dose) vs FP + loratadine | Very serious | Not serious | Not serious | Serious | Not serious | Moderate | Not serious | Not serious | Moderate | Not serious | Not serious | NMA | Moderate |
| Loratadine (double dose) vs FP + loratadine | Serious | Not serious | Not serious | Serious | Not serious | Low | Not serious | Not serious | Low | Not serious | Not serious | NMA | Low |
| Loratadine (double dose) vs loratadine + montelukast | Not serious | Not serious | Not serious | Serious | Not serious | Low | Not serious | Not serious | Moderate | Not serious | Not serious | NMA | Moderate |
| Montelukast (double dose) vs loratadine + montelukast | Not serious | Not serious | Not serious | Not serious | Not serious | High | Not serious | Not serious | High | Not serious | Not serious | NMA | High |
| Mometasone (double dose) vs mometasone + oxymetazoline | Very serious | Not serious | Serious | Serious | Not serious | Low | Not serious | Not serious | Low | Not serious | Not serious | NMA | Low |
| Oxymetazoline (double dose) vs mometasone + oxymetazoline | Very serious | Not serious | Serious | Serious | Not serious | Very low | Not serious | Not serious | Very low | Not serious | Not serious | NMA | Very low |
| Mometasone (double dose) vs olopatadine-mometasone | Extremely serious | Not serious | Serious | Serious | Not serious | Very low | Not serious | Not serious | Very low | Not serious | Not serious | NMA | Very low |
| Olopatadine (double dose) vs olopatadine-mometasone | Not serious | Not serious | Serious | Serious | Not serious | Very low | Not serious | Extremely serious | Moderate | Not serious | Extremely serious | NMA | Very low |

CoE: Certainty of Evidence; FF: Fluticasone furoate; FP: Fluticasone propionate; NMA: Network meta-analysis

**Supplementary Table 17. Full description of the research implications of the systematic reviews**

The table can be found at:

https://docs.google.com/spreadsheets/d/1IL9Kr8FtKPJM0riEW0FPzGweH1N_7FmA/edit?usp=sharing&ouid=111541831006622640146&rtpof=true&sd=true

**Supplementary Figure 1. Flow diagram for primary study selection**

**Supplementary Figure 2. Risk of bias graph considering all included randomized controlled trials**

RQLQ=Rhinoconjunctivitis quality of life questionnaire; rTNSS=Reflective total nasal symptom score; rTOSS=Reflective total ocular symptom score

**Supplementary Figure 3. Risk of bias graphs for studies in (A) adults with perennial allergic rhinitis (*N*=62), (B) adults with seasonal allergic rhinitis (*N*=162), (C) children with perennial allergic rhinitis (*N*=22), and (D) children with seasonal allergic rhinitis (*N*=13)**

RQLQ=Rhinoconjunctivitis quality of life questionnaire; rTNSS=Reflective total nasal symptom score; rTOSS=Reflective total ocular symptom score

**Supplementary Figure 4. Comparison between doubling the dose of the medication on each row to adding the medication of each column on the occurrence of withdrawal due to adverse events (main analysis: randomized controlled trials evaluating patients of all ages and with either seasonal or perennial allergic rhinitis).** Example interpretation of results: for the first non-empty cell in the first column, a double dose of ciclesonide is associated with 2 more withdrawals due to adverse events per 1000 individuals (from 3 fewer to 11 more per 1000) compared to ciclesonide + azelastine.

|  | Azelastine | Olopatadine | Ciclesonide | FF | FP | Mometasone | Oxymetazoline | Bilastine | Fexofenadine | Loratadine | Montelukast |
| --- | --- | --- | --- | --- | --- | --- | --- | --- | --- | --- | --- |
| Azelastine |  |  | **12 more per 1000 (from 3 more to 26 more)** |  | 1 fewer per 1000 (from 4 fewer to 4 more) |  |  |  | 3 more per 1000 (from 1 fewer to 11 more) | 4 more per 1000 (from 1 fewer to 15 more) |  |
| Olopatadine |  |  |  |  |  | 8 more per 1000 (from 2 fewer to 27 more) |  |  |  |  |  |
| Ciclesonide | 2 more per 1000 (from 3 fewer to 11 more) |  |  |  |  |  |  |  |  |  |  |
| FF |  |  |  |  |  |  | 9 fewer per 1000 (from 12 fewer to 11 more) |  |  |  |  |
| FP | **4 fewer per 1000 (from 6 fewer to 3 fewer)** |  |  |  |  |  |  |  |  | **2 more per 1000 (from 0 more to 8 more)** |  |
| Mometasone |  | **2 more per 1000 (from 1 more to 4 more)** |  |  |  |  | 2 more per 1000 (from 9 fewer to 48 more) |  |  |  |  |
| Oxymetazoline |  |  |  | **6 fewer per 1000 (from 9 fewer to 2 fewer)** |  | 5 more per 1000 (from 7 fewer to 42 more) |  |  |  |  |  |
| Bilastine |  |  |  |  |  |  |  |  |  |  | 2 fewer per 1000 (from 4 fewer to 2 more) |
| Fexofenadine | 3 more per 1000 (from 1 fewer to 22 more) |  |  |  |  |  |  |  |  |  |  |
| Loratadine | **3 more per 1000 (from 0 more to 8 more)** |  |  |  | **2 more per 1000 (from 0 more to 9 more)** |  |  |  |  |  | **8 more per 1000 (from 7 more to 9 more)** |
| Montelukast |  |  |  |  |  |  |  | 11 more per 1000 (from 4 fewer to 149 more) |  | **10 more per 1000 (from 7 more to 15 more)** |  |

FF=Fluticasone furoate; FP=Fluticasone propionate

| Cell shade (effect size) | | | | | | |
| --- | --- | --- | --- | --- | --- | --- |
| Large effect  (favouring double dose) | Moderate effect  (favouring double dose) | Small effect  (favouring double dose) | Trivial effect | Small effect  (favouring co-medication) | Moderate effect  (favouring co-medication) | Large effect  (favouring co-medication) |

| Text colour (certainty of evidence) | | | |
| --- | --- | --- | --- |
| Very Low | Low | Moderate | High |

**Supplementary Box 1. PRISMA NMA Checklist of Items to Include When Reporting A Systematic Review Involving a Network Meta-analysis**

| **Section/Topic** | **Item #** | **Checklist Item** | **Reported on Page #** |
| --- | --- | --- | --- |
| **TITLE** |  |  |  |
| Title | 1 | Identify the report as a systematic review *incorporating a network meta-analysis (or related form of meta-analysis).* | 1 |
|  |  |  |  |
| **ABSTRACT** |  |  | 2 |
| Structured summary | 2 | Provide a structured summary including, as applicable:  **Background:** main objectives  **Methods:** data sources; study eligibility criteria, participants, and interventions; study appraisal; and *synthesis methods, such as network meta-analysis.*  **Results:** number of studies and participants identified; summary estimates with corresponding confidence/credible intervals; *treatment rankings may also be discussed. Authors may choose to summarize pairwise comparisons against a chosen treatment included in their analyses for brevity.*  **Discussion/Conclusions:** limitations; conclusions and implications of findings.  **Other:** primary source of funding; systematic review registration number with registry name. |  |
|  |  |  |  |
| **INTRODUCTION** |  |  |  |
| Rationale | 3 | Describe the rationale for the review in the context of what is already known*, including mention of why a network meta-analysis has been conducted.* | 4 |
| Objectives | 4 | Provide an explicit statement of questions being addressed, with reference to participants, interventions, comparisons, outcomes, and study design (PICOS). | 4 |
|  |  |  |  |
| **METHODS** |  |  |  |
| Protocol and registration | 5 | Indicate whether a review protocol exists and if and where it can be accessed (e.g., Web address); and, if available, provide registration information, including registration number. | 5 |
| Eligibility criteria | 6 | Specify study characteristics (e.g., PICOS, length of follow-up) and report characteristics (e.g., years considered, language, publication status) used as criteria for eligibility, giving rationale. *Clearly describe eligible treatments included in the treatment network, and note whether any have been clustered or merged into the same node (with justification).* | 5; Supplementary Methods (page 1) |
| Information sources | 7 | Describe all information sources (e.g., databases with dates of coverage, contact with study authors to identify additional studies) in the search and date last searched. | 5; Supplementary Methods (page 2) |
| Search | 8 | Present full electronic search strategy for at least one database, including any limits used, such that it could be repeated. | 5, Supplementary Table 1 |
| Study selection | 9 | State the process for selecting studies (i.e., screening, eligibility, included in systematic review, and, if applicable, included in the meta-analysis). | 5, 6; Supplementary Methods (page 2) |
| Data collection process | 10 | Describe method of data extraction from reports (e.g., piloted forms, independently, in duplicate) and any processes for obtaining and confirming data from investigators. | 6 |
| Data items | 11 | List and define all variables for which data were sought (e.g., PICOS, funding sources) and any assumptions and simplifications made. | 5, 6, Supplementary Methods (page 2) |
| Geometry of the network | S1 | Describe methods used to explore the geometry of the treatment network under study and potential biases related to it. This should include how the evidence base has been graphically summarized for presentation, and what characteristics were compiled and used to describe the evidence base to readers. | 6, 7 |
| Risk of bias within individual studies | 12 | Describe methods used for assessing risk of bias of individual studies (including specification of whether this was done at the study or outcome level), and how this information is to be used in any data synthesis. | 6; Supplementary Methods (page 2) |
| Summary measures | 13 | State the principal summary measures (e.g., risk ratio, difference in means). *Also describe the use of additional summary measures assessed, such as treatment rankings and surface under the cumulative ranking curve (SUCRA) values, as well as modified approaches used to present summary findings from meta-analyses.* | 6, 7; Supplementary Methods (page 3) |
| Planned methods of analysis | 14 | Describe the methods of handling data and combining results of studies for each network meta-analysis. This should include, but not be limited to:   - *Handling of multi-arm trials;* - *Selection of variance structure;* - *Selection of prior distributions in Bayesian analyses; and* - *Assessment of model fit.* | 6, 7, Supplementary Methods (pages 3-5) |
| Assessment of Inconsistency | S2 | Describe the statistical methods used to evaluate the agreement of direct and indirect evidence in the treatment network(s) studied. Describe efforts taken to address its presence when found. | 6; Supplementary Methods (page 5) |
| Risk of bias across studies | 15 | Specify any assessment of risk of bias that may affect the cumulative evidence (e.g., publication bias, selective reporting within studies). | 6; Supplementary Methods (pages 2-3) |
| Additional analyses | 16 | Describe methods of additional analyses if done, indicating which were pre-specified. This may include, but not be limited to, the following:   - Sensitivity or subgroup analyses; - Meta-regression analyses; - *Alternative formulations of the treatment network; and* - *Use of alternative prior distributions for Bayesian analyses (if applicable).* | 7; Supplementary Methods (page 4) |
|  |  |  |  |
| **RESULTS†** |  |  |  |
| Study selection | 17 | Give numbers of studies screened, assessed for eligibility, and included in the review, with reasons for exclusions at each stage, ideally with a flow diagram. | 8, Supplementary Figure 1 |
| Presentation of network structure | S3 | Provide a network graph of the included studies to enable visualization of the geometry of the treatment network. | Not applicable |
| Summary of network geometry | S4 | Provide a brief overview of characteristics of the treatment network. This may include commentary on the abundance of trials and randomized patients for the different interventions and pairwise comparisons in the network, gaps of evidence in the treatment network, and potential biases reflected by the network structure. | 9, 10, 11 |
| Study characteristics | 18 | For each study, present characteristics for which data were extracted (e.g., study size, PICOS, follow-up period) and provide the citations. | Supplementary Table 4 |
| Risk of bias within studies | 19 | Present data on risk of bias of each study and, if available, any outcome level assessment. | Figure 1, Supplementary Table 5 |
| Results of individual studies | 20 | For all outcomes considered (benefits or harms), present, for each study: 1) simple summary data for each intervention group, and 2) effect estimates and confidence intervals. *Modified approaches may be needed to deal with information from larger networks.* | Described in previous reviews |
| Synthesis of results | 21 | Present results of each meta-analysis done, including confidence/credible intervals. *In larger networks, authors may focus on comparisons versus a particular comparator (e.g. placebo or standard care), with full findings presented in an appendix. League tables and forest plots may be considered to summarize pairwise comparisons.* If additional summary measures were explored (such as treatment rankings), these should also be presented. | Table 1, Table 2 |
| Exploration for inconsistency | S5 | Describe results from investigations of inconsistency. This may include such information as measures of model fit to compare consistency and inconsistency models, *P* values from statistical tests, or summary of inconsistency estimates from different parts of the treatment network. | Table 4 |
| Risk of bias across studies | 22 | Present results of any assessment of risk of bias across studies for the evidence base being studied. | Table 4 |
| Results of additional analyses | 23 | Give results of additional analyses, if done (e.g., sensitivity or subgroup analyses, meta-regression analyses*, alternative network geometries studied, alternative choice of prior distributions for Bayesian analyses,* and so forth). | Figure 3, Supplementary Table 9, Supplementary Table 10, Supplementary Table 13, Supplementary Table 14, Supplementary Table 15 |
| **DISCUSSION** |  |  |  |
| Summary of evidence | 24 | Summarize the main findings, including the strength of evidence for each main outcome; consider their relevance to key groups (e.g., healthcare providers, users, and policy-makers). | 12 |
| Limitations | 25 | Discuss limitations at study and outcome level (e.g., risk of bias), and at review level (e.g., incomplete retrieval of identified research, reporting bias). *Comment on the validity of the assumptions, such as transitivity and consistency. Comment on any concerns regarding network geometry (e.g., avoidance of certain comparisons).* | 13 |
| Conclusions | 26 | Provide a general interpretation of the results in the context of other evidence, and implications for future research. | 14 |
|  |  |  |  |
| **FUNDING** |  |  | 2 |
| Funding | 27 | Describe sources of funding for the systematic review and other support (e.g., supply of data); role of funders for the systematic review. This should also include information regarding whether funding has been received from manufacturers of treatments in the network and/or whether some of the authors are content experts with professional conflicts of interest that could affect use of treatments in the network. |  |

**References**

1.	Hutton B, Salanti G, Caldwell DM, et al. The PRISMA extension statement for reporting of systematic reviews incorporating network meta-analyses of health care interventions: checklist and explanations. *Ann Intern Med*. Jun 2 2015;162(11):777-84. doi:10.7326/M14-2385

2.	Sousa-Pinto B, Vieira RJ, Gil-Mata S, et al. Treatment dose increase versus comedication in allergic rhinitis: Systematic review with dose-response network meta-analysis protocol. *Rev Port Imunoalergologia* 2025;33:1-9

3.	Bousquet J, Schunemann HJ, Sousa-Pinto B, et al. Concepts for the Development of Person-Centered, Digitally Enabled, Artificial Intelligence-Assisted ARIA Care Pathways (ARIA 2024). *J Allergy Clin Immunol Pract*. Oct 2024;12(10):2648-2668 e2. doi:10.1016/j.jaip.2024.06.040

4.	Bousquet J, Sousa-Pinto B, Vieira RJ, Schunemann H, Zuberbier T, Bognanni A. Methodology for the development of the Allergic Rhinitis and its Impact on Asthma (ARIA) 2024-2025 guidelines: From Evidence-to-Decision frameworks to digitalised shared decision-making algorithms

5.	Food and Drug Administration. *Allergic Rhinitis: Developing Drug Products for Treatment - Guidance for Industry*. 2018. <https://www.fda.gov/files/drugs/published/Allergic-Rhinitis--Developing-Drug-Products-for-Treatment-Guidance-for-Industry.pdf>

6.	Gil-Mata S, Vieira RJ, Borowiack E, et al. Intranasal treatments for allergic rhinitis in preschool- and school-aged children: network meta-analysis. *J Allergy Clin Immunol Pract*. Jul 15 2025;doi:10.1016/j.jaip.2025.07.004

7.	Sousa-Pinto B, Vieira RJ, Bognanni A, et al. Efficacy and safety of intranasal medications for allergic rhinitis: Network meta-analysis. *Allergy*. 2025;80(1):94-105. doi:10.1111/all.16384

8.	Sousa-Pinto B, Vieira RJ, Brozek J, et al. Intranasal antihistamines and corticosteroids in allergic rhinitis: A systematic review and meta-analysis. *J Allergy Clin Immunol*. 2024;154(2):340-354. doi:10.1016/j.jaci.2024.04.016

9.	Thomander T, Gil-Mata S, Riera-Serra P, et al. Efficacy and safety of oral treatments for allergic rhinitis in children: A systematic review and network meta-analysis. (Under review).

10.	Torres MI, Gil-Mata S, Bognanni A, et al. Intranasal Versus Oral Treatments for Allergic Rhinitis: A Systematic Review With Meta-Analysis. *J Allergy Clin Immunol Pract*. 2024;12(12):3404-3418. doi:10.1016/j.jaip.2024.09.001

11.	Vieira RJ, Gil-Mata S, Ferreira A, et al. Efficacy and safety of oral antihistamines for allergic rhinitis: Network meta-analysis. *J Allergy Clin Immunol Pract*. 2026:S2213-2198(26)00140-6. doi: 10.1016/j.jaip.2025.12.034.

12.	Higgins JP, Altman DG, Gotzsche PC, et al. The Cochrane Collaboration's tool for assessing risk of bias in randomised trials. *BMJ*. Oct 18 2011;343:d5928. doi:10.1136/bmj.d5928

13.	Brignardello-Petersen R, Florez ID, Izcovich A, et al. GRADE approach to drawing conclusions from a network meta-analysis using a minimally contextualised framework. *BMJ*. 2020;371:m3900. doi:10.1136/bmj.m3900

14.	Brignardello-Petersen R, Izcovich A, Rochwerg B, et al. GRADE approach to drawing conclusions from a network meta-analysis using a partially contextualised framework. *BMJ*. 2020;371:m3907. doi:10.1136/bmj.m3907

15.	The GRADE Working Group. *The GRADE Book version 1.0 (updated September 2024).*

16.	Cohen J. *Statistical power analysis for the behavioral sciences*. routledge; 2013.

17.	Sousa-Pinto B, Bognanni A, Gil-Mata S, et al. Empirical estimation of disutilities and decision thresholds for composite endpoints. *J Clin Epidemiol*. 2025;179:111638. doi:10.1016/j.jclinepi.2024.111638

18.	Weir CJ, Butcher I, Assi V, et al. Dealing with missing standard deviation and mean values in meta-analysis of continuous outcomes: a systematic review. *BMC Med Res Methodol*. 2018;18(1):25. doi:10.1186/s12874-018-0483-0

19.	Petropoulou M, Rucker G, Schwarzer G. Network meta-analysis with dose-response relationships. *BMC Med Res Methodol*. 2026;doi:10.1186/s12874-025-02754-4

20.	Pedder H, Dias S, Bennetts M, Boucher M, Welton NJ. Joining the Dots: Linking Disconnected Networks of Evidence Using Dose-Response Model-Based Network Meta-Analysis. *Med Decis Making*. 2021;41(2):194-208. doi:10.1177/0272989X20983315

21.	Spector S, Bronsky E, Chervinsky P, et al. Multicenter, double-blind, placebo-controlled trial of triamcinolone acetonide nasal aerosol in the treatment of perennial allergic rhinitis. *Ann Allergy*. 1990;64(3):300‐305.

22.	Day JH, Andersson CB, Briscoe MP. Efficacy and safety of intranasal budesonide in the treatment of perennial rhinitis in adults and children. *Ann Allergy*. 1990;64(5):445‐450.

23.	Storms W, Bronsky E, Findlay S, et al. Once daily triamcinolone acetonide nasal spray is effective for the treatment of perennial allergic rhinitis. *Annals of allergy*. 1991;66(4):329‐334.

24.	van As A, Bronsky EA, Dockhorn RJ, et al. Once daily fluticasone propionate is as effective for perennial allergic rhinitis as twice daily beclomethasone diproprionate. *J Allergy Clin Immunol*. 1993;91(6):1146-54. doi:10.1016/0091-6749(93)90317-9

25.	Banov CH, Woehler TR, LaForce CF, et al. Once daily intranasal fluticasone propionate is effective for perennial allergic rhinitis. *Ann Allergy*. 1994;73(3):240-6.

26.	Kobayashi RH, Beaucher WN, Koepke JW, et al. Triamcinolone acetonide aqueous nasal spray for the treatment of patients with perennial allergic rhinitis: a multicenter, randomized, double-blind, placebo-controlled study. *Clin Ther*. 1995;17(3):503‐513.

27.	Drouin M, Yang WH, Bertrand B, et al. Once daily mometasone furoate aqueous nasal spray is as effective as twice daily beclomethasone dipropionate for treating perennial allergic rhinitis patients. *Ann Allergy Asthma Immunol*. 1996;77(2):153-60. doi:10.1016/s1081-1206(10)63502-8

28.	Mandl M, Nolop K, Lutsky BN. Comparison of once daily mometasone furoate (Nasonex) and fluticasone propionate aqueous nasal sprays for the treatment of perennial rhinitis. The 194-079 Study Group. *Ann Allergy Asthma Immunol*. Sep 1997;79(3):237-45.

29.	Bachert C, Brostoff J, Scadding GK, Tasman J, Stalla-Bourdillon A, Murrieta M. Mizolastine therapy also has an effect on nasal blockade in perennial allergic rhinoconjunctivitis. *Allergy*. 1998;53(10):969--975. doi:10.1111/j.1398-9995.1998.tb03798.x

30.	Davies RJ. Efficacy and tolerability comparison of ebastine 10 and 20 mg with loratadine 10 mg. A double-blind, randomised study in patients with perennial allergic rhinitis. *Clinical Drug Investigation*. 1998;16(6):413-420. doi:10.2165/00044011-199816060-00001

31.	Day J, Carrillo T. Comparison of the efficacy of budesonide and fluticasone propionate aqueous nasal spray for once daily treatment of perennial allergic rhinitis. *J Allergy Clin Immunol*. 1998;102(6 Pt 1):902-8. doi:10.1016/s0091-6749(98)70326-4

32.	Meltzer EO. Clinical and antiinflammatory effects of intranasal budesonide aqueous pump spray in the treatment of perennial allergic rhinitis. *Annals Allergy Asthma Immunol*. 1998;81(2):128‐134.

33.	Murris-Espin M, Melac M, Charpentier JC, Didier A. Comparison of efficacy and safety of cetirizine and ebastine in patients with perennial allergic rhinitis. *Ann Allergy Asthma Immunol*. 1998;80(5):399-403. doi:10.1016/S1081-1206(10)62991-2

34.	Stern MA, Wade AG, Ridout SM, Cambell LM. Nasal budesonide offers superior symptom relief in perennial allergic rhinitis in comparison to nasal azelastine. *Ann Allergy Asthma Immunol*. 1998;81(4):354-8. doi:10.1016/s1081-1206(10)63128-6

35.	Bousquet J, Gaudaño EM, Carlos AGP, Staudinger H. A 12-week, placebo-controlled study of the efficacy and safety of ebastine, 10 and 20 mg once daily, in the treatment of perennial allergic rhinitis. *Allergy*. 1999;54(6):562-568. doi:10.1034/j.1398-9995.1999.00984.x

36.	Holm AF, Godthelp T, Fokkens WJ, et al. Long-term effects of corticosteroid nasal spray on nasal inflammatory cells in patients with perennial allergic rhinitis. *Clin Exp Allergy*. 1999;29(10):1356-66. doi:10.1046/j.1365-2222.1999.00665.x

37.	Kivisaari E, Baker RC, Price MJ. Comparison of once daily fluticasone propionate aqueous nasal spray with once daily budesonide reservoir powder device in patients with perennial rhinitis. *Clin Exp Allergy*. 2001;31(6):855-63. doi:10.1046/j.1365-2222.2001.01097.x

38.	Weckx L, Fujita R, Pignatari S, et al. Randomized open-label comparative study between triamcinolone acetonide nasal spray versus beclometasone dipropionate in perennial rhinitis in adults. *Revista Brasileira de Medicina*. 2001;58(10):774-782.

39.	Bende M, Carrillo T, Vona I, da Castel-Branco MG, Arheden L. A randomized comparison of the effects of budesonide and mometasone furoate aqueous nasal sprays on nasal peak flow rate and symptoms in perennial allergic rhinitis. *Ann Allergy Asthma Immunol*. 2002;88(6):617-23. doi:10.1016/s1081-1206(10)61894-7

40.	Frèche C, Leynadier F, Horak F, et al. Mizolastine provides effective symptom relief in patients suffering from perennial allergic rhinitis: A double-blind, placebo-controlled study versus loratadine. *Ann Allergy Asthma Immunol*. 2002;89(3):304-310. doi:10.1016/S1081-1206(10)61959-X

41.	*R1810220 - An efficacy and safety study of fluticasone propionate (FP) aqueous nasal spray in subjects with perennial allergic rhinitis (PAR)*

42.	Simons FER, Prenner BM, Finn A. Efficacy and safety of desloratadine in the treatment of perennial allergic rhinitis. *Journal of Allergy and Clinical Immunology*. 2003;111(3):617--622. doi:10.1067/mai.2003.168

43.	Bachert C, Bousquet J, Canonica GW, et al. Levocetirizine improves quality of life and reduces costs in long-term management of persistent allergic rhinitis. *J Allergy Clin Immunol*. 2004;114(4):838-844. doi:10.1016/j.jaci.2004.05.070

44.	Aguiar FAB, Campos CRP. Comparative, prospective, placebo controlled study of mometasone and triamcinolone for the treatment of allergic rhinitis. *Revista brasileira de medicina*. 2006;63(5):211‐214.

45.	Chervinsky P, Kunjibettu S, Miller DL, et al. Long-term safety and efficacy of intranasal ciclesonide in adult and adolescent patients with perennial allergic rhinitis. *Ann Allergy Asthma Immunol*. 2007;99(1):69‐76.

46.	Meltzer EO, Kunjibettu S, Hall N, et al. Efficacy and safety of ciclesonide, 200 microg once daily, for the treatment of perennial allergic rhinitis. *Ann Allergy Asthma Immunol*. 2007;98(2):175‐181.

47.	Philip G, Williams-Herman D, Patel P, et al. Efficacy of montelukast for treating perennial allergic rhinitis. *Allergy Asthma Proc*. 2007;28(3):296--304. doi:10.2500/aap.2007.28.3000

48.	Rosenblut A, Bardin PG, Muller B, et al. Long-term safety of fluticasone furoate nasal spray in adults and adolescents with perennial allergic rhinitis. *Allergy*. 2007;62(9):1071-7. doi:10.1111/j.1398-9995.2007.01521.x

49.	Fantin S, Maspero J, Bisbal C, et al. A 12-week placebo-controlled study of rupatadine 10 mg once daily compared with cetirizine 10 mg once daily, in the treatment of persistent allergic rhinitis. *Allergy*. 2008;63(7):924-931. doi:10.1111/j.1398-9995.2008.01668.x

50.	Nathan RA, Berger W, Yang W, et al. Effect of once-daily fluticasone furoate nasal spray on nasal symptoms in adults and adolescents with perennial allergic rhinitis. *Ann Allergy Asthma Immunol*. 2008;100(5):497-505. doi:10.1016/S1081-1206(10)60477-2

51.	Patel D, Ratner P, Clements D, Wu W, Faris M, Philpot E. Lack of effect on adult and adolescent hypothalamic-pituitary-adrenal axis function with use of fluticasone furoate nasal spray. *Ann Allergy Asthma Immunol*. May 2008;100(5):490-6. doi:10.1016/S1081-1206(10)60476-0

52.	Vasar M, Houle PA, Douglass JA, et al. Fluticasone furoate nasal spray: effective monotherapy for symptoms of perennial allergic rhinitis in adults/adolescents. *Allergy Asthma Proc*. 2008;29(3):313-21. doi:10.2500/aap.2008.29.3126

53.	Holmberg K, Tonnel AB, Dreyfus I, et al. Desloratadine relieves nasal congestion and improves quality-of-life in persistent allergic rhinitis. *Allergy*. 2009 2009;64(11):1663‐1670. doi:doi:10.1111/j.1398-9995.2009.02096.x

54.	Kowalski ML, Jurkiewicz D, Kruszewski J, Nowak D, Zietkowski Z, piakov. Rupatadine 10 and 20 mg are effective and safe in the treatment of perennial allergic rhinitis after 4 weeks of treatment: a randomized, double-blind, controlled trial with loratadine and placebo. *Therapy*. 2009;6(3):417--425. doi:10.2217/thy.09.1

55.	Bernstein J, Jacobs J, Wheeler W, Sacks H. Efficacy of Azelastine 0.15% Nasal Spray in Treating Nasal Symptoms of Perennial Allergic Rhinitis. *Journal of Allergy and Clinical Immunology*. 2010;125(2):AB176.

56.	Bousquet J, Bachert C, Canonica GW, et al. Efficacy of desloratadine in persistent allergic rhinitis - a GA(2)LEN study. *Int Arch Allergy Immunol*. 2010;153(4):395-402. doi:10.1159/00031635110.1159/000316351.

57.	Given JT, Cheema AS, Dreykluft T, et al. Fluticasone furoate nasal spray is effective and well tolerated for perennial allergic rhinitis in adolescents and adults. *Am J Rhinol Allergy*. 2010;24(6):444-50. doi:10.2500/ajra.2010.24.3534

58.	Grossman J, Gates D. Mometasone furoate nasal spray for the treatment of elderly patients with perennial allergic rhinitis. *Ann Allergy Asthma Immunol*. May 2010;104(5):452-3. doi:10.1016/j.anai.2010.03.007

59.	Meltzer EO, Munafo DA, Chung W, Gopalan G, Varghese ST. Intranasal mometasone furoate therapy for allergic rhinitis symptoms and rhinitis-disturbed sleep. *Ann Allergy Asthma Immunol*. 2010;105(1):65-74. doi:10.1016/j.anai.2010.04.020

60.	Molina M, Cister. Rupatadine 10 mg in adolescent and adult symptom relief of perennial allergic rhinitis. *Therapy*. 2010;7(4):429.

61.	*NCT01033825 - A 6-Week Randomized, Double-Blind, Placebo-Controlled, Parallel Group, Safety and Efficacy Study of the Potential Inhibitory Effects on the Hypothalamic-Pituitary-Adrenal Axis of Ciclesonide HFA Nasal Aerosol and Ciclesonide Aqueous Nasal Spray in Subjects 12 Years and Older With Perennial Allergic Rhinitis*.

62.	Baroody FM, Brown D, Gavanescu L, DeTineo M, Naclerio RM. Oxymetazoline adds to the effectiveness of fluticasone furoate in the treatment of perennial allergic rhinitis. *J Allergy Clin Immunol*. 2011;127(4):927-34. doi:10.1016/j.jaci.2011.01.037

63.	Marmouz F, Giralt J, Izquierdo I. Morning and evening efficacy evaluation of rupatadine (10 and 20 mg), compared with cetirizine 10 mg in perennial allergic rhinitis: A randomized, double-blind, placebo-controlled trial. *J Asthma Allergy*. 2011;4:27--35. doi:10.2147/JAA.S18265

64.	Berger WE, Mohar DE, LaForce C, et al. A 26-week tolerability study of ciclesonide nasal aerosol in patients with perennial allergic rhinitis. *Am J Rhinol Allergy*. 2012;26(4):302‐307.

65.	Fokkens WJ, Rinia B, van Drunen CM, et al. No mucosal atrophy and reduced inflammatory cells: active-controlled trial with yearlong fluticasone furoate nasal spray. *Am J Rhinol Allergy*. 2012;26(1):36-44. doi:10.2500/ajra.2012.26.3675

66.	Guilemany JM, García-Piñero A, Alobid I, et al. The loss of smell in persistent allergic rhinitis is improved by levocetirizine due to reduction of nasal inflammation but not nasal congestion (the CIRANO study). *International Archives of Allergy and Immunology*. 2012;158(2):184-190. doi:10.1159/000331329

67.	Meltzer EO, Jacobs RL, LaForce CF, Kelley CL, Dunbar SA, Tantry SK. Safety and efficacy of once-daily treatment with beclomethasone dipropionate nasal aerosol in subjects with perennial allergic rhinitis. *Allergy Asthma Proc*. 2012;33(3):249‐257.

68.	Aneeza WH, Husain S, Rahman RA, Van Dort D, Abdullah A, Gendeh BS. Efficacy of mometasone furoate and fluticasone furoate on persistent allergic rhinoconjunctivitis. *Allergy Rhinol (Providence)*. 2013;4(3):e120-6. doi:10.2500/ar.2013.4.0065

69.	*2013-000654-22 - Randomised, double-blind, double-dummy, vehicle controlled parallel trial comparing a novel mometasone furoate nasal spray vs. Nasonex® nasal spray vs. vehicle nasal spray in patients with allergic rhinitis*

70.	LaForce C, Journeay GE, Miller SD, et al. Ocular safety of fluticasone furoate nasal spray in patients with perennial allergic rhinitis: a 2-year study. *Ann Allergy Asthma Immunol*. 2013;111(1):45-50. doi:10.1016/j.anai.2013.04.013

71.	Goh BS, Ismail MI, Husain S. Quality of life assessment in patients with moderate to severe allergic rhinitis treated with montelukast and/or intranasal steroids: a randomised, double-blind, placebo-controlled study. *J Laryngol Otol*. Mar 2014;128(3):242-8. doi:10.1017/S002221511400036X

72.	Skoner DP, LaForce CF, Nathan RA, et al. Effect of cetirizine on symptom severity and quality of life in perennial allergic rhinitis. *Allergy Asthma Proc* 2014;35(4):338-345. doi:<https://dx.doi.org/10.2500/aap.2014.35.3760>

73.	Weinstein SF, Andrews CP, Shah SR, et al. Long-term efficacy and safety of once-daily treatment with beclomethasone dipropionate nasal aerosol. *Allergy and asthma proceedings*. 2014 2014;35(4):323‐331. doi:doi:

74.	Jia MH, Chen XY, Zhang Y, Liao ZS. [Effect of nasal glucocorticoid combined with loratadine or montelukast on allergic rhinitis]. *Lin Chuang Er Bi Yan Hou Tou Jing Wai Ke Za Zhi*. 2017;31(5):369-373. doi:10.13201/j.issn.1001-1781.2017.05.010

75.	Lv C, Zhang Y, Shen L. Preliminary Clinical Effect Evaluation of Resveratrol in Adults with Allergic Rhinitis. *International archives of allergy and immunology*. 2018;175(4):231‐236.

76.	Karaulov AV, Vylegzhanina T, Ovchinnikov A, Chernikova M, Nenasheva N. Triamcinolone Acetonide versus Fluticasone Propionate in the Treatment of Perennial Allergic Rhinitis: A Randomized, Parallel-Group Trial. *Int Arch Allergy Immunol*. 2019;179(2):142-151. doi:10.1159/000497160

77.	Segall N, Prenner B, Lumry W, Caracta CF, Tantry SK. Long-term safety and efficacy of olopatadine-mometasone combination nasal spray in patients with perennial allergic rhinitis. *Allergy Asthma Proc*. 2019;40(5):301-310. doi:10.2500/aap.2019.40.4233

78.	Mo JH, Jeong JH, Kim TH, et al. Double-Blind Placebo-Controlled Trial of Bepotastine Salicylate in Patients With Allergic Rhinitis. *The Laryngoscope*. 2020;131(3):E702--E709. doi:10.1002/lary.28906

79.	Wagner K, Franklin K, Larona A, Zhang L, Tian X. Budesonide 128ug and 256ug significantly relieves moderate-to-severe par nasal symptoms and congestion. *Ann Allergy Asthma Immunol*. 2020;125(5):S47‐.

80.	Won TB, Kim HG, Kim JW, et al. Efficacy and safety of rupatadine fumarate in the treatment of perennial allergic rhinitis: a multicenter, double-blinded, randomized, placebo-controlled, bridging study in Koreans. *Asian pacific journal of allergy and immunology*. 2021;doi:10.12932/ap-201220-1019

81.	*NCT00720382 - A Study to Evaluate the Safety and Tolerability of a Nasal Spray to Treat Perennial Allergic Rhinitis*.

82.	Andersen JB, Halberg P, Mygind N. Beclomethasone dipropionate aerosol treatment of hay fever. A dose-response investigation. *Acta allergologica*. 1975;30(5):316‐325.

83.	Ep M, Søborg M, Nørreslet TT, Mygind N. A Comparative Study of Dexchlorpheniramine Maleate Sustained Release Tablets and Budesonide Nasal Spray in Seasonal Allergic Rhinitis. *Allergy*. 1983;38(7):517-524. doi:10.1111/j.1398-9995.1983.tb02361.x

84.	Gutkowski A, Carpio JD, Gelinas B, Schulz J, Turenne Y. Comparative Study of the Efficacy, Tolerance and Side-Effects of Dexchlorpheniramine Maleate 6 Mg B.I.D. with Terfenadine 60 Mg B.I.D. *Journal of International Medical Research*. 1985;13(5):284-288. doi:10.1177/030006058501300507

85.	Skassa-Brociek W, Bousquet J, Montes F, et al. Double-blind placebo-controlled study of loratadine, mequitazine, and placebo in the symptomatic treatment of seasonal allergic rhinitis. *J Allergy Clin Immunol*. 1988;81(4):725-730.

86.	Tinkelman D, Falliers C, Gross G, et al. Multicenter evaluation of triamcinolone acetonide nasal aerosol in the treatment of adult patients with seasonal allergic rhinitis. *Ann Allergy*. 1990;64(2):234‐240.

87.	Meltzer EO, Orgel HA, Bronsky EA, et al. A dose-ranging study of fluticasone propionate aqueous nasal spray for seasonal allergic rhinitis assessed by symptoms, rhinomanometry, and nasal cytology. *J Allergy Clin Immunol*. Aug 1990;86(2):221-30. doi:10.1016/s0091-6749(05)80069-7

88.	Nathan RA, Bronsky EA, Fireman P, et al. Once daily fluticasone propionate aqueous nasal spray is an effective treatment for seasonal allergic rhinitis. *Ann Allergy*. 1991;67(3):332-8.

89.	van As A, Bronsky E, Grossman J, Meltzer E, Ratner P, Reed C. Dose tolerance study of fluticasone propionate aqueous nasal spray in patients with seasonal allergic rhinitis. *Ann Allergy*. 1991;67(2):156‐162.

90.	*FLN-270 - A double-blind, randomized, placebo-controlled, parallel group study comparing fluticasone propionate aqueous nasal spray 200µg QD manufactured with two different procedures in patients with seasonal allergic rhinitis*.

91.	Findlay S, Huber F, Garcia J, Lee HA. Efficacy of once-a-day intranasal administration of triamcinolone acetonide in patients with seasonal allergic rhinitis. *Ann Allergy*. 1992;68(3):228-232.

92.	Norman PS, Creticos PS, Tobey R, et al. Budesonide in grass pollen rhinitis. *Ann Allergy*. 1992;69(4):309‐316. doi:doi:

93.	Ratner PH, Paull BR, Findlay SR, et al. Fluticasone propionate given once daily is as effective for seasonal allergic rhinitis as beclomethasone dipropionate given twice daily. *J Allergy Clin Immunol*. 1992;90(3 Pt 1):285-91. doi:10.1016/s0091-6749(05)80005-3

94.	Dockhorn RJ, Paull BR, Meltzer EO, et al. Once-versus twice-daily fluticasone propionate aqueous nasal spray for seasonal allergic rhinitis. *Am J Rhinol*. 1993;7(2):77-83.

95.	*NCT03855189 - Safety and Efficacy of Mometasone Furoate (SCH 032088) vs Beclomethasone Dipropionate or Placebo in Seasonal Allergic Rhinitis (C93-013)*.

96.	Dahl R, Pedersen B, Larsen B. Intranasal levocabastine for the treatment of seasonal allergic rhinitis: a multicentre, double-blind, placebo-controlled trial. *Rhinology*. 1995;33(3):121‐125.

97.	LaForce CF, Dockhorn RJ, Findlay SR, et al. Fluticasone propionate: an effective alternative treatment for seasonal allergic rhinitis in adults and adolescents. *J Fam Pract*. Feb 1994;38(2):145-52.

98.	Mackowiak J. Fluticasone propionate aqueous nasal spray (FP) improves rhinitis quality of life and reduces lost labor cost. *Ann Allergy*. 1994;72:99.

99.	McArthur JG. A comparison of budesonide and beclomethasone dipropionate sprays in the treatment of seasonal allergic rhinitis. *Clin Otolaryngol Allied Sci*. 1994;19(6):537-42. doi:10.1111/j.1365-2273.1994.tb01285.x

100.	Munk Z, Pearlman D, Graft D, et al. Intranasal fluticasone propionate is effective and well-tolerated in adolescents with seasonal allergic rhinitis. *Pediatric asthma, allergy & immunology*. 1994;8(1):39-46.

101.	van Bavel J, Findlay SR, Hampel FC, Jr., Martin BG, Ratner P, Field E. Intranasal fluticasone propionate is more effective than terfenadine tablets for seasonal allergic rhinitis. *Arch Intern Med*. 1994;154(23):2699-704. doi:10.1001/archinte.1994.00420230086010

102.	*NCT03882047 - Mometasone Furoate Aqueous (MK-0887/SCH 032088) Nasal Spray vs Placebo and FLONASE® in Seasonal Allergic Rhinitis Patients (I94-001)*.

103.	Bronsky E, Boggs P, Findlay S, et al. Comparative efficacy and safety of a once-daily loratadine-pseudoephedrine combination versus its components alone and placebo in the management of seasonal allergic rhinitis. *The Journal of Allergy and Clinical Immunology*. 1995;96(2):139-147. doi:10.1016/S0091-6749(95)70001-3

104.	Pelucchi A, Chiapparino A, Mastropasqua B, Marazzini L, Hernandez A, Foresi A. Effect of intranasal azelastine and beclomethasone dipropionate on nasal symptoms, nasal cytology, and bronchial responsiveness to methacholine in allergic rhinitis in response to grass pollens. *J Allergy Clin Immunol*. 1995;95(2):515-23. doi:10.1016/s0091-6749(95)70313-6

105.	Prenner BM, Chervinsky P, Hampel FC, et al. Double-strength beclomethasone dipropionate (84 micrograms/spray) aqueous nasal spray in the treatment of seasonal allergic rhinitis. *J Allergy Clin Immunol*. 1996;98(2):302‐308.

106.	Settipane G, Korenblat PE, Winder J, et al. Triamcinolone acetonide Aqueous nasal spray in patients with seasonal ragweed allergic rhinitis: a placebo-controlled, double-blind study. *Clin Ther*. 1995;17(2):252‐263.

107.	Bernstein DI, Creticos PS, Busse WW, et al. Comparison of triamcinolone acetonide nasal inhaler with astemizole in the treatment of ragweed-induced allergic rhinitis. *J Allergy Clin Immunol*. 1996;97(3):749-755. doi:10.1016/s0091-6749(96)80151-5

108.	Bronsky EA, Dockhorn RJ, Meltzer EO, et al. Fluticasone propionate aqueous nasal spray compared with terfenadine tablets in the treatment of seasonal allergic rhinitis. *J Allergy Clin Immunol*. 1996;97(4):915-21. doi:10.1016/s0091-6749(96)80065-0

109.	*FLTA4004 - Double-Blind, Double-Dummy, Randomized, Parallel-Group Comparison of the Efficacy and Safety Outcomes of Fluticasone Propionate Aqueous Nasal Spray Versus Encapsulated Loratadine Tablets Versus Placebo for Four Weeks in Subjects with Seasonal Allergic Rhinitis*.

110.	Harvey RP, Comer C, Sanders B, et al. Model for outcomes assessment of antihistamine use for seasonal allergic rhinitis. *J Allergy Clin Immunol*. 1996;97(6):1233-1241. doi:10.1016/S0091-6749(96)70190-2

111.	Hebert JR, Nolop K, Lutsky BN. Once-daily mometasone furoate aqueous nasal spray (Nasonex) in seasonal allergic rhinitis: an active- and placebo-controlled study. *Allergy*. 1996;51(8):569-76. doi:10.1111/j.1398-9995.1996.tb04670.x

112.	Howland WC, 3rd. Fluticasone propionate: topical or systemic effects? *Clin Exp Allergy*. 1996;26 Suppl 3:18-22. doi:10.1111/j.1365-2222.1996.tb00654.x

113.	Howland WC, 3rd, Hampel FC, Jr., Martin BG, Ratner PH, van Bavel JH, Field EA. The efficacy of fluticasone propionate aqueous nasal spray for allergic rhinitis and its relationship to topical effects. *Clin Ther*. 1996;18(6):1106-17. doi:10.1016/s0149-2918(96)80065-8

114.	Munk ZM, LaForce C, Furst JA, Simpson B, Feiss G, Smith JA. Efficacy and safety of triamcinolone acetonide aqueous nasal spray in patients with seasonal allergic rhinitis. *Ann Allergy Asthma Immunol*. 1996;77(4):277‐281.

115.	Schoenwetter W, Lim J. Comparison of intranasal triamcinolone acetonide with oral loratadine for the treatment of patients with seasonal allergic rhinitis. *Clin Ther*. 1995;17(3):479-492. doi:10.1016/0149-2918(95)80113-8

116.	Alvarado-Valdes CA, Blomgren J, Weiler D, et al. The effect of fluticasone propionate aqueous nasal spray on eosinophils and cytokines in nasal secretions of patients with ragweed allergic rhinitis. *Clin Ther*. 1997;19(2):273-81. doi:10.1016/s0149-2918(97)80115-4

117.	Day J, Alexander M, Drouin M, et al. Budesonide aqueous nasal spray and pressurized metered dose inhaler in the treatment of adult patients with seasonal allergic rhinitis. *Am J Rhinol*. 1997;11(1):77‐83.

118.	Gawchik SM, Lim J. Comparison of intranasal triamcinolone acetonide with oral loratadine in the treatment of seasonal ragweed-induced allergic rhinitis. *Am J Manag Care*. 1997;3(7):1052-8.

119.	Géhanno P, Desfougeres JL. Fluticasone propionate aqueous nasal spray compared with oral loratadine in patients with seasonal allergic rhinitis. *Allergy*. 1997;52(4):445-450. doi:10.1111/j.1398-9995.1997.tb01027.x

120.	Small P, Houle PA, Day JH, et al. A comparison of triamcinolone acetonide nasal aerosol spray and fluticasone propionate aqueous solution spray in the treatment of spring allergic rhinitis. *J Allergy Clin Immunol*. 1997;100(5):592-5. doi:10.1016/s0091-6749(97)70160-x

121.	Stern MA, Dahl R, Nielsen LP, Pedersen B, Schrewelius C. A comparison of aqueous suspensions of budesonide nasal spray (128 micrograms and 256 micrograms once daily) and fluticasone propionate nasal spray (200 micrograms once daily) in the treatment of adult patients with seasonal allergic rhinitis. *Am J Rhinol*. 1997;11(4):323-30. doi:10.2500/105065897781446658

122.	Creticos P, Fireman P, Settipane G, et al. Intranasal budesonide aqueous pump spray (Rhinocort (R) Aqua) for the treatment of seasonal allergic rhinitis. *Allergy Asthma Proc*. 1998;19(5):285-294.

123.	Meltzer EO, Jalowayski AA, Orgel HA, Harris AG. Subjective and objective assessments in patients with seasonal allergic rhinitis: effects of therapy with mometasone furoate nasal spray. *J Allergy Clin Immunol*. 1998;102(1):39-49. doi:10.1016/s0091-6749(98)70053-3

124.	Ratner PH, van Bavel JH, Martin BG, et al. A comparison of the efficacy of fluticasone propionate aqueous nasal spray and loratadine, alone and in combination, for the treatment of seasonal allergic rhinitis. *J Fam Pract*. 1998;47(2):118-25.

125.	Rosenthal R, Berger W, Bronsky E, et al. Tri-Nasal triamcinolone acetonide nasal spray 200 and 400 micrograms qd versus placebo and Nasacort triamcinolone acetonide nasal aerosol 440 micrograms qd in patients suffering from seasonal allergic rhinitis during the grass season. *Am J Rhinol*. 1998;12(6):427‐433.

126.	Stricker W, Nathan R, Sinder J. Fluticasone propionate aqueous nasal spray and loratadine, alone and in combination, in the treatment of fall seasonal allergic rhinitis. *Ann Allergy Asthma Immunol*. 1998;80:115.

127.	Berkowitz RB, Bernstein DI, LaForce C, et al. Onset of action of mometasone furoate nasal spray (NASONEX) in seasonal allergic rhinitis. *Allergy*. 1999;54(1):64-9. doi:10.1034/j.1398-9995.1999.00713.x

128.	Casale TB, Andrade C, Qu R. Safety and efficacy of once-daily fexofenadine HCI in the treatment of autumn seasonal allergic rhinitis. *Allergy Asthma Proc*. 1999-5 1999;20(3):193-198. doi:doi:10.2500/108854199778553046

129.	Di Lorenzo G, Gervasi F, Drago A, et al. Comparison of the effects of fluticasone propionate, aqueous nasal spray and levocabastine on inflammatory cells in nasal lavage and clinical activity during the pollen season in seasonal rhinitics. *Clin Exp Allergy*. 1999;29(10):1367-77. doi:10.1046/j.1365-2222.1999.00643.x

130.	Hampel FC, Martin BG, Dolen J, Travers S, Karcher K, Holton D. Efficacy and safety of levocabastine nasal spray for seasonal allergic rhinitis. *Am J Rhinol*. 1999;13(1):55‐62.

131.	Kremer B, Klimek L, Gulicher D, Degen M, Mosges R. Sequential therapy with azelastine in seasonal allergic rhinitis. Deutsche Rhinitis Studiengruppe (German Rhinitis Study Group). *Arzneimittelforschung*. 1999;49(11):912-9. doi:10.1055/s-0031-1300526

132.	Tanner LA, Reilly M, Meltzer EO, Bradford JE, Mason J. Effect of fexofenadine HCl on quality of life and work, classroom, and daily activity impairment in patients with seasonal allergic rhinitis. *American journal of managed care*. 1999;5(4):S235‐S247.

133.	Condemi JJ, Schulz RM, Lim J. Triamcinolone acetonide aqueous nasal spray versus loratadine in seasonal allergic rhinitis: efficacy and quality of life. *Ann Allergy Asthma Immunol*. 2000;84(5):533-538. doi:10.1016/s1081-1206(10)62518-5

134.	Meltzer EO, Malmstrom K, Lu S, et al. Concomitant montelukast and loratadine as treatment for seasonal allergic rhinitis: a randomized, placebo-controlled clinical trial. *J Allergy Clin Immunol*. 2000;105(5):917-22. doi:10.1067/mai.2000.106040

135.	Van Cauwenberge P, Juniper EF. Comparison of the efficacy, safety and quality of life provided by fexofenadine hydrochloride 120 mg, loratadine 10 mg and placebo administered once daily for the treatment of seasonal allergic rhinitis. *Clin Exp Allergy*. 2000;30(6):891-899. doi:10.1046/j.1365-2222.2000.00914.x

136.	Falser N, Wober W, Rahlfs VW, Baehre M. Comparative efficacy and safety of azelastine and levocabastine nasal sprays in patients with seasonal allergic rhinitis. *Arzneimittelforschung*. 2001;51(5):387-93. doi:10.1055/s-0031-1300052

137.	*FNM30034 - A Multi-Center, Randomized, Double-Blind, Placebo-Controlled, Parallel-Group Study to Assess the Efficacy of a Four-Week Course of Fluticasone Propionate Aqueous Nasal Spray (200mcg QD) on Ocular Symptoms Commonly Associated with Allergic Rhinitis*.

138.	*R1810221 - An efficacy and safety study of fluticasone propionate (FP) aqueous nasal spray in subjects with seasonal allergic rhinitis (SAR)*.

139.	*NCT00641680 - Rhinocort Aqua Versus Placebo and Fluticasone Propionate*.

140.	Berger WE, Schenkel EJ, Mansfield LE. Safety and efficacy of desloratadine 5 mg in asthma patients with seasonal allergic rhinitis and nasal congestion. *Ann Allergy Asthma Immunol*. 2002;89(5):485-491. doi:10.1016/S1081-1206(10)62086-8

141.	Ciprandi G, Canonica WG, Grosclaude M, Ostinelli J, Brazzola GG, Bousquet J. Effects of budesonide and fluticasone propionate in a placebo-controlled study on symptoms and quality of life in seasonal allergic rhinitis. *Allergy*. 2002;57(7):586-91. doi:10.1034/j.1398-9995.2002.03228.x

142.	Gross G, Jacobs RL, Woodworth TH, Georges GC, Lim JC. Comparative efficacy, safety, and effect on quality of life of triamcinolone acetonide and fluticasone propionate aqueous nasal sprays in patients with fall seasonal allergic rhinitis. *Ann Allergy Asthma Immunol*. 2002;89(1):56-62. doi:10.1016/S1081-1206(10)61911-4

143.	Nayak AS, Philip G, Lu S, Malice M-P, Reiss TF, Group MFRI. Efficacy and tolerability of montelukast alone or in combination with loratadine in seasonal allergic rhinitis: a multicenter, randomized, double-blind, placebo-controlled trial performed in the fall. *Ann Allergy Asthma Immunol*. 2002;88(6):592-600.

144.	Philip G, Malmstrom K, Hampel FC, et al. Montelukast for treating seasonal allergic rhinitis: A randomized, double-blind, placebo-controlled trial performed in the spring. *Clin Exp Allergy*. 2002;32(7):1020-1028. doi:10.1046/j.1365-2222.2002.01422.x

145.	Salmun LM, Lorber R. 24-hour efficacy of once-daily desloratadine therapy in patients with seasonal allergic rhinitis [ISRCTN32042139]. *BMC Family Practice*. 2002;3:1-6.

146.	Berger WE, Kaiser H, Gawchik SM, et al. Triamcinolone acetonide aqueous nasal spray and fluticasone propionate are equally effective for relief of nasal symptoms in patients with seasonal allergic rhinitis. *Otolaryngol Head Neck Surg*. Jul 2003;129(1):16-23. doi:10.1016/S0194-59980300526-6

147.	Berger WE, White MV, Rhinitis Study G. Efficacy of azelastine nasal spray in patients with an unsatisfactory response to loratadine. *Ann Allergy Asthma Immunol*. 2003;91(2):205-11. doi:10.1016/S1081-1206(10)62179-5

148.	Dykewicz MS, Kaiser HB, Nathan RA, et al. Fluticasone propionate aqueous nasal spray improves nasal symptoms of seasonal allergic rhinitis when used as needed (prn). *Ann Allergy Asthma Immunol*. 2003;91(1):44-8. doi:10.1016/S1081-1206(10)62057-1

149.	Gawchik S, Goldstein S, Prenner B, John A. Relief of cough and nasal symptoms associated with allergic rhinitis by mometasone furoate nasal spray. *Ann Allergy Asthma Immunol*. 2003;90(4):416-21. doi:10.1016/S1081-1206(10)61826-1

150.	Lumry W, Hampel F, LaForce C, Kiechel F, el-Akkad T, Murray JJ. A comparison of once-daily triamcinolone acetonide aqueous and twice-daily beclomethasone dipropionate aqueous nasal sprays in the treatment of seasonal allergic rhinitis. *Allergy Asthma Proc*. 2003;24(3):203-10.

151.	Noonan MJ, Raphael GD, Nayak A, et al. The health-related quality of life effects of once-daily cetirizine HCl in patients with seasonal allergic rhinitis: A randomized double-blind, placebo-controlled trial. *Clin Exp Allergy*. 2003;33(3):351-358. doi:10.1046/j.1365-2222.2003.01596.x

152.	Ratner PH, Howland WC, Arastu R, et al. Fluticasone propionate aqueous nasal spray provided significantly greater improvement in daytime and nighttime nasal symptoms of seasonal allergic rhinitis compared with montelukast. *Ann Allergy Asthma Immunol*. 2003;90(5):536-542. doi:10.1016/s1081-1206(10)61847-9

153.	Van Adelsberg J, Philip G, LaForce CF, et al. Randomized controlled trial evaluating the clinical benefit of montelukast for treating spring seasonal allergic rhinitis. *Ann Allergy Asthma Immunol* 2003;90(2):214-222. doi:10.1016/S1081-1206(10)62144-8

154.	Van Adelsberg J, Philip G, Pedinoff AJ, et al. Montelukast improves symptoms of seasonal allergic rhinitis over a 4-week treatment period. *Allergy*. 2003;58(12):1268-1276. doi:10.1046/j.1398-9995.2003.00261.x

155.	Bernstein DI, Levy AL, Hampel FC, et al. Treatment with intranasal fluticasone propionate significantly improves ocular symptoms in patients with seasonal allergic rhinitis. *Clin Exp Allergy*. 2004;34(6):952-7. doi:10.1111/j.1365-2222.2004.01952.x

156.	Chervinsky P, Philip G, Malice MP, et al. Montelukast for treating fall allergic rhinitis: Effect of pollen exposure in 3 studies. *Ann Allergy Asthma Immunol*. 2004;92(3):367-373. doi:10.1016/S1081-1206(10)61576-1

157.	Hampel F, Howland W, Van Bavel J, Ratner P. A randomized, double-blind, placebo-controlled study comparing the efficacy and safety of ebastine (20 mg and 10 mg) to loratadine 10 mg once daily in the treatment of seasonal allergic rhinitis. *Journal of Investigational Allergology and Clinical Immunology*. 2004;14(1):56-63.

158.	LaForce CF, Corren J, Wheeler WJ, Berger WE, Rhinitis Study G. Efficacy of azelastine nasal spray in seasonal allergic rhinitis patients who remain symptomatic after treatment with fexofenadine. *Ann Allergy Asthma Immunol*. 2004;93(2):154-9. doi:10.1016/S1081-1206(10)61468-8

159.	Ratner P, Hampel F, Jr., Van Bavel J, Howland W, 3rd. Efficacy and safety of ebastine 20 mg compared to loratadine 10 mg once daily in the treatment of seasonal allergic rhinitis: a randomized, double-blind, placebo-controlled study. *Int Arch Allergy Immunol*. 2004;133(4):371-9. doi:10.1159/000077357

160.	Bhatia S, Baroody FM, deTineo M, Naclerio RM. Increased nasal airflow with budesonide compared with desloratadine during the allergy season. *Arch Otolaryngol*. 2005;131(3):223-228. doi:10.1001/archotol.131.3.223

161.	Corren J, Storms WW, Bernstein JA, Berger WE, Nayak A, Sacks H. Effectiveness of Azelastine Nasal Spray Compared with Oral Cetirizine in Patients with Seasonal Allergic Rhinitis. *Clinical therapeutics*. 2005;27(5):543-553. doi:10.1016/j.clinthera.2005.04.012

162.	Meltzer EO, Hampel FC, Ratner PH, et al. Safety and efficacy of olopatadine hydrochloride nasal spray for the treatment of seasonal allergic rhinitis. *Ann Allergy Asthma Immunol*. 2005;95(6):600‐606. doi:doi:

163.	Okubo K, Gotoh M, Shimada K, Ritsu M, Okuda M, Crawford B. Fexofenadine improves the quality of life and work productivity in Japanese patients with seasonal allergic rhinitis during the peak cedar pollinosis season. *Int Arch Allergy Immunol*. 2005;136(2):148-54. doi:10.1159/000083322

164.	Ratner PH, Hampel FC, Amar NJ, et al. Safety and efficacy of olopatadine hydrochloride nasal spray for the treatment of seasonal allergic rhinitis to mountain cedar. *Ann Allergy Asthma Immunol*. 2005;95(5):474-479.

165.	Berger WE, Hampel FC, Bernstein JA, Shah S, Sacks H, Meltzer EO. Impact of azelastine nasal spray on symptoms and quality of life compared with cetirizine oral tablets in patients with seasonal allergic rhinitis. *Ann Allergy Asthma Immunol*. 2006;97(3):375-381. doi:10.1016/s1081-1206(10)60804-6

166.	Martin BG, Andrews CP, van Bavel J, et al. Comparison of fluticasone propionate aqueous nasal spray and oral montelukast for the treatment of seasonal allergic rhinitis symptoms. *Ann Allergy Asthma Immunol*. 2006;96(6):851-857. doi:10.1016/s1081-1206(10)61349-x

167.	Meltzer EO, Jalowayski AA, Vogt K, Iezzoni D, Harris AG. Effect of desloratadine therapy on symptom scores and measures of nasal patency in seasonal allergic rhinitis: Results of a single-center, placebo-controlled trial. *Ann Allergy Asthma Immunol*. 2006;96(2):363-368. doi:10.1016/S1081-1206(10)61249-5

168.	Ratner PH, Wingertzahn MA, van Bavel JH, et al. Effectiveness of ciclesonide nasal spray in the treatment of seasonal allergic rhinitis. *Ann Allergy Asthma Immunol.* 2006;97(5):657‐663.

169.	Ratner PH, Wingertzahn MA, van Bavel JH, Hampel F, Darken PF, Shah T. Efficacy and safety of ciclesonide nasal spray for the treatment of seasonal allergic rhinitis. *J Allergy Clin Immunol*. 2006 2006;118(5):1142‐1148.

170.	*NCT00358527 - Mometasone Furoate on Sleep Disturbances in Subjects With Seasonal Allergic Rhinitis (Study P04608)*.

171.	Fokkens WJ, Jogi R, Reinartz S, et al. Once daily fluticasone furoate nasal spray is effective in seasonal allergic rhinitis caused by grass pollen. *Allergy*. 2007;62(9):1078-84. doi:10.1111/j.1398-9995.2007.01522.x

172.	Kaiser HB, Naclerio RM, Given J, Toler TN, Ellsworth A, Philpot EE. Fluticasone furoate nasal spray: a single treatment option for the symptoms of seasonal allergic rhinitis. *J Allergy Clin Immunol*. 2007;119(6):1430-7. doi:10.1016/j.jaci.2007.02.022

173.	Lumry W, Prenner B, Corren J, Wheeler W. Efficacy and safety of azelastine nasal spray at a dose of 1 spray per nostril twice daily. *Ann Allergy Asthma Immunol*. 2007;99(3):267-72. doi:10.1016/S1081-1206(10)60663-1

174.	Martin BG, Ratner PH, Hampel FC, et al. Optimal dose selection of fluticasone furoate nasal spray for the treatment of seasonal allergic rhinitis in adults and adolescents. *Allergy Asthma Proc*. 2007;28(2):216-25. doi:10.2500/aap.2007.28.2983

175.	Pradalier A, Neukirch C, Dreyfus I, Devillier P. Desloratadine improves quality of life and symptom severity in patients with allergic rhinitis. *Allergy*. 2007;62(11):1331-4. doi:10.1111/j.1398-9995.2007.01374.x

176.	*NCT00453063 - Placebo-Controlled Study of Mometasone Furoate Nasal Spray (MFNS) 200 mcg Once Daily (QD) in the Treatment of Seasonal Allergic Rhinitis (Study P05067)*.

177.	Anolik R, Mometasone Furoate Nasal Spray With Loratadine Study G. Clinical benefits of combination treatment with mometasone furoate nasal spray and loratadine vs monotherapy with mometasone furoate in the treatment of seasonal allergic rhinitis. *Ann Allergy Asthma Immunol*. 2008;100(3):264-71. doi:10.1016/S1081-1206(10)60452-8

178.	Ratner PH, Hampel F, Van Bavel J, et al. Combination therapy with azelastine hydrochloride nasal spray and fluticasone propionate nasal spray in the treatment of patients with seasonal allergic rhinitis. *Ann Allergy Asthma Immunol*. 2008;100(1):74-81. doi:10.1016/S1081-1206(10)60408-5

179.	*NCT00733005 - Study of Nasonex in the Relief of Nasal Congestion in Patients With Seasonal Allergic Rhinitis (Study P05528)*.

180.	Andrews CP, Martin BG, Jacobs RL, et al. Fluticasone furoate nasal spray is more effective than fexofenadine for nighttime symptoms of seasonal allergy. *Allergy Asthma Proc*. 2009;30(2):128-38. doi:10.2500/aap.2009.30.3204

181.	Bachert C, Kuna P, Sanquer F, et al. Comparison of the efficacy and safety of bilastine 20 mg vs desloratadine 5 mg in seasonal allergic rhinitis patients. *Allergy*. 2009;64(1):158-165. doi:10.1111/j.1398-9995.2008.01813.x

182.	Bernstein JA, Prenner B, Ferguson BJ, Portnoy J, Wheeler WJ, Sacks HJ. Double-blind, placebo-controlled trial of reformulated azelastine nasal spray in patients with seasonal allergic rhinitis. *Am J Rhinol Allergy*. 2009;23(5):512-7. doi:10.2500/ajra.2009.23.3396

183.	Bousquet J, Bindslev-Jensen C, Sanquer F, Zuberbier T. Symptom improvement correlates with improvement in daily functioning in subjects with persistent allergic rhinitis treated with desloratadine 5 mg daily: results from the aerius control: clinical and evaluative profile of treatment-2 (ACCEPT2) trial in collaboration with global allergy and asthma European network. *Allergy*. 2009 2009;64:596. doi:doi:10.1111/j.1398-9995.2009.02078.x

184.	Brandi V, Stahl EG. Clinical equivalence between generic and branded fluticasone propionate nasal spray in patients with allergic rhinitis. 2009;

185.	Demoly P, Dreyfus I, Dhivert-Donnadieu H, Mesbah K. Desloratadine for the treatment of cypress pollen-induced allergic rhinitis. *Ann Allergy Asthma Immunol*. 2009;103(3):260-266. doi:10.1016/S1081-1206(10)60191-3

186.	Jacobs R, Martin B, Hampel F, Toler W, Ellsworth A, Philpot E. Effectiveness of fluticasone furoate 110 microg once daily in the treatment of nasal and ocular symptoms of seasonal allergic rhinitis in adults and adolescents sensitized to mountain cedar pollen. *Curr Med Res Opin*. 2009;25(6):1393-401. doi:10.1185/03007990902890512

187.	Kaliner MA, Storms W, Tilles S, et al. Comparison of olopatadine 0.6% nasal spray versus fluticasone propionate 50 µg in the treatment of seasonal allergic rhinitis. 2009:255.

188.	Kuna P, Bachert C, Nowacki Z, et al. Efficacy and safety of bilastine 20 mg compared with cetirizine 10 mg and placebo for the symptomatic treatment of seasonal allergic rhinitis: A randomized, double-blind, parallel-group study. *Clin Exp Allergy*. 2009;39(9):1338-1347. doi:10.1111/j.1365-2222.2009.03257.x

189.	LaForce C, van Bavel J, Meltzer EO, Wingertzahn MA. Efficacy and safety of ciclesonide hydrofluoroalkane nasal aerosol once daily for the treatment of seasonal allergic rhinitis. *Ann Allergy Asthma Immunol*. 2009 2009;103(2):166‐173.

190.	Lu S, Malice MP, Dass SB, Reiss TF. Clinical studies of combination montelukast and loratadine in patients with seasonal allergic rhinitis. *J Asthma*. 2009;46(9):878‐883.

191.	Lu S, Malice M-P, Dass SB, Reiss TF. Clinical Studies of Combination Montelukast and Loratadine in Patients with Seasonal Allergic Rhinitis. *J Asthma*. 2009;46(9):878-883. doi:10.3109/02770900903104540

192.	Nathan R, Gopalan G, Gates D. Mometasone furoate nasal spray monotherapy compared with loradatine in subjects with seasonal allergic rhinitis provides superior relief of nasal symptoms and comparable relief of ocular symptoms. 2009:70-71.

193.	Okubo K, Nakashima M, Miyake N, Komatsubara M, Okuda M. Comparison of fluticasone furoate and fluticasone propionate for the treatment of Japanese cedar pollinosis. *Allergy Asthma Proc*. 2009;30(1):84-94. doi:10.2500/aap.2009.30.3182

194.	Prenner B, Anolik R, Danzig M, Yao R. Efficacy and safety of fixed-dose loratadine/montelukast in seasonal allergic rhinitis: effects on nasal congestion. *Allergy Asthma Proc*. 2009;30(3):263-9. doi:10.2500/aap.2009.30.3228

195.	Shah S, Berger W, Lumry W, La Force C, Wheeler W, Sacks H. Efficacy and safety of azelastine 0.15% nasal spray and azelastine 0.10% nasal spray in patients with seasonal allergic rhinitis. *Allergy Asthma Proc*. 2009;30(6):628-33. doi:10.2500/aap.2009.30.3296

196.	Shah SR, Nayak A, Ratner P, Roland P, Michael Wall G. Effects of olopatadine hydrochloride nasal spray 0.6% in the treatment of seasonal allergic rhinitis: a phase III, multicenter, randomized, double-blind, active- and placebo-controlled study in adolescents and adults. *Clin Ther*. 2009;31(1):99-107. doi:10.1016/j.clinthera.2009.01.016

197.	van Bavel J, Howland WC, Amar NJ, Wheeler W, Sacks H. Efficacy and safety of azelastine 0.15% nasal spray administered once daily in subjects with seasonal allergic rhinitis. *Allergy Asthma Proc*. 2009;30(5):512-8. doi:10.2500/aap.2009.30.3284

198.	Hampel FC, Ratner PH, Van Bavel J, et al. Double-blind, placebo-controlled study of azelastine and fluticasone in a single nasal spray delivery device. *Ann Allergy Asthma Immunol*. 2010;105(2):168-73. doi:10.1016/j.anai.2010.06.008

199.	Maiti R, Rahman J, Jaida J, Allala U, Palani A. Rupatadine and Levocetirizine for Seasonal Allergic Rhinitis. *Archives of Otolaryngology–Head & Neck Surgery*. 2010;136(8):796-796. doi:10.1001/archoto.2010.128

200.	Mansfield LE, Hampel F, Haeusler JM, Georges G. Study of levocetirizine in seasonal allergic rhinitis. *Curr Med Res Opin*. Jun 2010;26(6):1269-75. doi:10.1185/03007991003745233

201.	Prenner BM, Lanier BQ, Bernstein DI, Shekar T, Teper A. Mometasone furoate nasal spray reduces the ocular symptoms of seasonal allergic rhinitis. *J Allergy Clin Immunol*. 2010;125(6):1247-1253 e5. doi:10.1016/j.jaci.2010.03.004

202.	Ratner P, Jacobs R, Mohar D, Huang H, Desai SY, Hinkle J. Evaluation of the efficacy and safety of ciclesonide hydrofluoroalkane nasal aerosol, 80 or 160 μg once daily, for the treatment of seasonal allergic rhinitis. *Ann Allergy Asthma Immunol*. 2010;105(6):471‐479.

203.	Segall N, Gawchik S, Georges G, Haeusler JMC. Efficacy and safety of levocetirizine in improving symptoms and health-related quality of life in US adults with seasonal allergic rhinitis: a randomized, placebo-controlled study. *Ann Allergy Asthma Immunol*. 2010;104(3):259-267. doi:10.1016/j.anai.2009.12.003

204.	Han D, Liu S, Zhang Y, et al. Efficacy and safety of fluticasone furoate nasal spray in Chinese adult and adolescent subjects with intermittent or persistent allergic rhinitis. *Allergy Asthma Proc*. 2011;32(6):472-81. doi:10.2500/aap.2011.32.3474

205.	Howland WC, Amar NJ, Wheeler W, Sacks H. Efficacy and safety of azelastine 0.15% nasal spray administered once daily in patients with allergy to Texas mountain cedar pollen. *Int Forum Allergy Rhinol*. 2011;1(4):275-9. doi:10.1002/alr.20065

206.	Maiti R, Jaida J, Rahman J, Gaddam R, Palani A. Olopatadine hydrochloride and rupatadine fumarate in seasonal allergic rhinitis: A comparative study of efficacy and safety. *Journal of pharmacology & pharmacotherapeutics*. 2011;2(4):270-276. doi:<https://dx.doi.org/10.4103/0976-500X.85958>

207.	Meltzer EO, Shekar T, Teper AA. Mometasone furoate nasal spray for moderate-to-severe nasal congestion in subjects with seasonal allergic rhinitis. *Allergy Asthma Proc*. 2011;32(2):159-67. doi:10.2500/aap.2011.32.3424

208.	Diaz A. Rhinitis Treatment 1: 149 Once Daily Intranasal Fluticasone Furoate Decreases Daytime Sleepiness and Improves Cognitive Performance in Symptomatic Seasonal Allergic Rhinitis. *The World Allergy Organization Journal*. 2012;5(Suppl 2):S66.

209.	Ratner PH, Andrews C, Martin B, et al. A study of the efficacy and safety of ciclesonide hydrofluoroalkane nasal aerosol in patients with seasonal allergic rhinitis from mountain cedar pollen. *Allergy Asthma Proc*. 2012 2012;33(1):27‐35.

210.	NCT01279057 - A Study Comparing Two Fluticasone Furoate Nasal Sprays in the Relief of the Signs and Symptoms of Seasonal Allergic Rhinitis.

211.	van Bavel JH, Ratner PH, Amar NJ, et al. Efficacy and safety of once-daily treatment with beclomethasone dipropionate nasal aerosol in subjects with seasonal allergic rhinitis. *Allergy Asthma Proc*. 2012;33(5):386‐396.

212.	Carr W, Bernstein J, Lieberman P, et al. A novel intranasal therapy of azelastine with fluticasone for the treatment of allergic rhinitis. *J Allergy Clin Immunol*. 2012;129(5):1282-1289 e10. doi:10.1016/j.jaci.2012.01.077

213.	Lukat K, Rivas P, Roger A, et al. A direct comparison of efficacy between desloratadine and rupatadine in seasonal allergic rhinoconjunctivitis: a randomized, double-blind, placebo-controlled study. *J Asthma Allergy*. 2013;6:31‐39. doi:10.2147/jaa.S39496

214.	Meltzer EO, Bernstein DI, Prenner BM, Berger WE, Shekar T, Teper AA. Mometasone furoate nasal spray plus oxymetazoline nasal spray: short-term efficacy and safety in seasonal allergic rhinitis. *Am J Rhinol Allergy*. Mar-Apr 2013;27(2):102-8. doi:10.2500/ajra.2013.27.3864

215.	Raphael GD, Berger WE, Prenner BM, Finn AF, Kelley L, Tantry SK. Efficacy, safety, and optimal dose selection of beclomethasone dipropionate nasal aerosol for seasonal allergic rhinitis in adolescents and adults. *Current medical research and opinion*. 2013;29(10):1329‐1340.:

216.	Schenkel E, Newman KB. Therapeutic equivalence of triamcinolone acetonide hydrofluoroalkane and chlorofluorocarbon nasal inhalers in patients with seasonal allergic rhinitis. *American journal of rhinology & allergy*. 2013 2013;27(2):109‐112.

217.	*NCT00719862 - A Study to Evaluate the Safety & Effectiveness of a Nasal Spray to Treat Seasonal Allergies*.

218.	*NCT00618332 - Can a Nasal Decongestant Test Predict Treatment Outcomes in Seasonal Allergic Rhinitis?*

219.	*NCT01038427 - A Study Comparing the Clinical Equivalence of Two Mometasone Nasal Sprays in the Relief of the Signs and Symptoms of Seasonal Allergic Rhinitis*.

220.	NCT02155881 - A Multi-Center, Double-Blind, Placebo-Controlled, Two-Arm, Parallel-Group, Phase III (Registration) Study to Assess the Efficacy and Safety of Ciclesonide Nasal Spray (Omnaris®) 200 mcg Once Daily in the Treatment of the Patients With Seasonal Allergic Rhinitis (SAR) in Russia.

221.	NCT02230696 - Comparative Safety and Efficacy of Two Steroid Treatments in the Relief of the Symptoms of Seasonal Allergic Rhinitis.

222.	*NCT01852513 - Allergic Rhinitis Changes the Sinus Microbiome*.

223.	*NCT01850823 - Clinical Equivalence Study of Mometasone Nasal Spray*.

224.	Kuna P, Wasiak W, Jones S, Kreft KZ. Comparative safety and efficacy of two formulations of mometasone nasal spray in adult seasonal allergic rhinitis. *Allergy Asthma Proc*. 2014;35(4):332-7. doi:10.2500/aap.2014.35.3770

225.	Ford LB, Matz J, Hankinson T, Prillaman B, Georges G. A comparison of fluticasone propionate nasal spray and cetirizine in ragweed fall seasonal allergic rhinitis. *Allergy Asthma Proc*. 2015;36(4):313-9. doi:10.2500/aap.2015.36.3860

226.	Kim CH, Kim JK, Kim HJ, et al. Comparison of Intranasal Ciclesonide, Oral Levocetirizine, and Combination Treatment for Allergic Rhinitis. *Allergy Asthma Immunol Res*. 2014;7(2):158-166. doi:10.4168/aair.2015.7.2.158

227.	Ratner P, Van Bavel J, Mohar D, et al. Efficacy of daily intranasal fluticasone propionate on ocular symptoms associated with seasonal allergic rhinitis. *Ann Allergy Asthma Immunol*. 2015;114(2):141-7. doi:10.1016/j.anai.2014.11.012

228.	*NCT02246920 - Clinical Equivalence Study of Fluticasone Propionate Nasal Spray, 50 mcg/Actuation vs. Flonase in Allergic Rhinitis Patients*.

229.	Hisada S Oshima N Weinstein Ci OKMYTY. A phase III multi-center, randomized, placebo-controlled, double-blind clinical trial to evaluate the efficacy and safety of desloratadine in Japanese patients with seasonal allergic rhinitis. *American journal of respiratory and critical care medicine*. 2016;193:A1393-A1393.

230.	Ratner P, Andrews CP, Hampel FC, et al. Efficacy and safety of setipiprant in seasonal allergic rhinitis: results from Phase 2 and Phase 3 randomized, double-blind, placebo- and active-referenced studies. *Allergy Asthma and Clinical Immunology*. 2017-4-4 2017;13doi:doi:10.1186/s13223-017-0183-z

231.	Okubo K, Suzuki T, Tanaka A, Aoki H. Efficacy and safety of rupatadine in Japanese patients with seasonal allergic rhinitis: A double-blind, randomized, multicenter, placebo-controlled clinical trial. *Allergol Int*. 2019;68(2):207-215. doi:10.1016/j.alit.2018.08.011

232.	Gross GN, Berman G, Amar NJ, Caracta CF, Tantry SK. Efficacy and safety of olopatadine-mometasone combination nasal spray for the treatment of seasonal allergic rhinitis. *Ann Allergy Asthma Immunol*. 2019;122(6):630-638 e3. doi:10.1016/j.anai.2019.03.017

233.	Hampel FC, Pedinoff AJ, Jacobs RL, Caracta CF, Tantry SK. Olopatadine-mometasone combination nasal spray: Evaluation of efficacy and safety in patients with seasonal allergic rhinitis. *Allergy Asthma Proc*. 2019;40(4):261-272. doi:10.2500/aap.2019.40.4223

234.	Ilyina NI, Edin AS, Astafieva NG, et al. Efficacy of a Novel Intranasal Formulation of Azelastine Hydrochloride and Fluticasone Propionate, Delivered in a Single Spray, for the Treatment of Seasonal Allergic Rhinitis: Results from Russia. *Int Arch Allergy Immunol*. 2019;178(3):255-263. doi:10.1159/000494507

235.	Andrews CP, Mohar D, Salhi Y, Tantry SK. Efficacy and safety of twice-daily and once-daily olopatadine-mometasone combination nasal spray for seasonal allergic rhinitis. *Ann Allergy Asthma Immunol*. 2020;124(2):171-178 e2. doi:10.1016/j.anai.2019.11.007

236.	Lavorini F, Matucci A, Rossi O, Pistolesi M, investigators Ss. Concomitant bilastine and montelukast as additive therapy for seasonal allergic rhinoconjunctivits and mild‐to‐moderate asthma. The SKY study. *Allergy*. 2019;75(3):675.

237.	Haruna T, Kariya S, Higaki T, et al. The add-on effect of an intranasal antihistamine with an intranasal corticosteroid in Japanese cedar pollinosis. *Auris Nasus Larynx*. Feb 2023;50(1):81-86. doi:10.1016/j.anl.2022.05.007

238.	Gendeh HS, Hamizan AW, Husain S, et al. The efficacy of elonide nasal corticosteroids in managing allergic rhinitis: a randomized, double-blinded trial. *J Clin Med*. 2024;13(7):1883.

239.	Storms WW, Southern DL, Feiss G, Simpson B, Furst JA, Smith JA. Efficacy of Triamcinolone Acetonide Aerosol Nasal Inhaler in Children with Perennial Allergic Rhinitis. *Pediatr Asthma Allergy Immunol*. 1996;10(2):59-64. doi:10.1089/pai.1996.10.59

240.	Fokkens WJ, Cserháti E, dos Santos JML, et al. Budesonide aqueous nasal spray is an effective treatment in children with perennial allergic rhinitis, with an onset of action within 12 hours. *Ann Allergy Asthma Immunol*. 2002;89(3):279-284. doi:10.1016/s1081-1206(10)61955-2

241.	Lai DS, Lue KH, Hsieh JC, Lin KL, Lee HS. The comparison of the efficacy and safety of cetirizine, oxatomide, ketotifen, and a placebo for the treatment of childhood perennial allergic rhinitis. *Ann Allergy Asthma Immunol*. 2002;89(6):589-98. doi:10.1016/S1081-1206(10)62107-2

242.	Potter PC, Paediatric Levocetirizine Study G. Efficacy and safety of levocetirizine on symptoms and health-related quality of life of children with perennial allergic rhinitis: a double-blind, placebo-controlled randomized clinical trial. *Ann Allergy Asthma Immunol*. 2005;95(2):175-80. doi:10.1016/S1081-1206(10)61208-2

243.	Chen ST, Lu KH, Sun HL, Chang WT, Lue KH, Chou MC. Randomized placebo-controlled trial comparing montelukast and cetirizine for treating perennial allergic rhinitis in children aged 2-6 yr. *Pediatr Allergy Immunol*. 2006;17(1):49-54. doi:10.1111/j.1399-3038.2005.00351.x

244.	Murphy KR, Uryniak T, Simpson B, O'Dowd L. Growth velocity in children with perennial allergic rhinitis treated with budesonide aqueous nasal spray. *Ann Allergy Asthma Immunol*. 2006;96(5):723-730. doi:10.1016/s1081-1206(10)61072-1

245.	Hung CH, Hua YM, Hsu WT, et al. Montelukast decreased exhaled nitric oxide in children with perennial allergic rhinitis. *Pediatr Int*. 2007;49(3):322-7. doi:10.1111/j.1442-200X.2007.02375.x

246.	Kim K, Weiswasser M, Nave R, et al. Safety of Once-Daily Ciclesonide Nasal Spray in Children 2 to 5 Years of Age with Perennial Allergic Rhinitis. *Pediatr Asthma Allergy Immunol*. 2007;20(4):229-242. doi:10.1089/pai.2007.002

247.	Berger WE, Nayak A, Lanier BQ, et al. Efficacy and Safety of Once-Daily Ciclesonide Nasal Spray in Children With Allergic Rhinitis. *Pediatr Asthma Allergy Immunol*. 2008;21(2):73-82. doi:10.1089/pai.2007.0022.73

248.	Lee CF, Sun HL, Lu KH, Ku MS, Lue KH. The comparison of cetirizine, levocetirizine and placebo for the treatment of childhood perennial allergic rhinitis. *Pediatr Allergy Immunol*. 2009;20(5):493-9. doi:10.1111/j.1399-3038.2008.00816.x

249.	Maspero J, Rosenblut A, Finn A, Lim J, Wu W, Philpot EE. Safety and efficacy of fluticasone furoate in pediatric patients with perennial allergic rhinitis. *Otolaryngol Head Neck Surg*. 2008;138(1):30-37. doi:10.1016/j.otohns.2007.10.023

250.	Tripathy I, Levy AL, Ratner PH, Clements D, Wu W, Philpot EE. HPA axis safety of fluticasone furoate nasal spray once daily in children with perennial allergic rhinitis. *Pediatr Allergy Immunol*. 2008;20(3):287-294. doi:10.1111/j.1399-3038.2008.00775.x

251.	Li AM, Abdullah VJ, Tsen CS, et al. Leukotriene receptor antagonist in the treatment of childhood allergic rhinitis--a randomized placebo-controlled study. *Pediatr Pulmonol*. 2009;44(11):1085-92. doi:10.1002/ppul.21102

252.	Weinstein S, Qaqundah P, Georges G, Nayak A. Efficacy and safety of triamcinolone acetonide aqueous nasal spray in children aged 2 to 5 years with perennial allergic rhinitis: a randomized, double-blind, placebo-controlled study with an open-label extension. *Ann Allergy Asthma Immunol*. 2009;102(4):339-347. doi:10.1016/s1081-1206(10)60340-7

253.	Baena-Cagnani CE, Patel P. Efficacy and long-term safety of mometasone furoate nasal spray in children with perennial allergic rhinitis. *Current medical research and opinion*. 2010;26(9):2047-2055. doi:10.1185/03007995.2010.487661

254.	Berman G, Hampel F, Wang B, Edwards M. An evaluation of the effect of ciclesonide hydrofluoroalkane nasal aerosol on hypothalamic pituitary adrenal axis in pediatric patients with perennial allergic rhinitis:A133-A134.

255.	Bernstein J, Meltzer S, Gever L, Ruiz N, Blessing-Moore J. Randomized, double-blind, placebo-controlled trial of the safety and efficacy of astepro (azelastine 0.15% solution and 0.10% solution) in children ages>= 6 to< 12 with perennial allergic rhinitis. 2014:A109-A109.

256.	Lee LA, Sterling R, Maspero J, Clements D, Ellsworth A, Pedersen S. Growth velocity reduced with once-daily fluticasone furoate nasal spray in prepubescent children with perennial allergic rhinitis. *J Allergy Clin Immunol Pract*. 2014;2(4):421-427. doi:10.1016/j.jaip.2014.04.008

257.	Berger WE, Jacobs RL, Amar NJ, Tantry SK, Li J, Small CJ. Efficacy and safety of beclomethasone dipropionate nasal aerosol in children with perennial allergic rhinitis. *Ann Allergy Asthma Immunol*. 2015;115(2):130-136. doi:10.1016/j.anai.2015.05.012

258.	Kim C-K, Hwang Y, Song DJ, et al. Efficacy and safety of montelukast+ levocetirizine combination therapy compared to montelukast monotherapy for allergic rhinitis in children. *Allergy Asthma Immunol Res*. 2024;16(6):652.

259.	Masi M, Candiani R, van de Venne H. A placebo-controlled trial of cetirizine in seasonal allergic rhino-conjunctivitis in children aged 6 to 12 years. *Pediatr Allergy Immunol*. 1993;4(4 Suppl):47-52. doi:10.1111/j.1399-3038.1993.tb00339.x

260.	Treatment of seasonal allergic rhinitis with once-daily intranasal fluticasone propionate therapy in children. Fluticasone Propionate Collaborative Pediatric Working Group. *The Journal of pediatrics*. 1994;125(4):628-634. doi:10.1016/s0022-3476(94)70025-7

261.	Banov CH, Silvers WS, Green AW, et al. Placebo-controlled, double-blind study of the efficacy and safety of triamcinolone acetonide aerosol nasal inhaler in pediatric patients with seasonal allergic rhinitis. *Clinical therapeutics*. 1996;18(2):265-272. doi:10.1016/s0149-2918(96)80007-5

262.	Schenkel EJ, Gross G, Jacobson K, et al. Triamcinolone Acetonide Aqueous Nasal Inhaler for the Treatment of Spring Grass Seasonal Allergic Rhinitis in Children. *Pediatr Asthma Allergy Immunol*. 1997;11(2):129-136. doi:10.1089/pai.1997.11.129

263.	Meltzer EO, Berger WE, Berkowitz RB, et al. A dose-ranging study of mometasone furoate aqueous nasal spray in children with seasonal allergic rhinitis. *J Allergy Clin Immunol*. 1999;104(1):107-114. doi:10.1016/s0091-6749(99)70121-1

264.	de Blic J, Wahn U, Billard E, Alt R, Pujazon MC. Levocetirizine in children: evidenced efficacy and safety in a 6-week randomized seasonal allergic rhinitis trial. *Pediatr Allergy Immunol*. 2005;16(3):267-75. doi:10.1111/j.1399-3038.2005.00216.x

265.	Berger WE, Ratner PH, Casale TB, Meltzer EO, Wall GM. Safety and efficacy of olopatadine hydrochloride nasal spray 0.6% in pediatric subjects with allergic rhinitis. *Allergy Asthma Proc*. 2009;30(6):612-623. doi:10.2500/aap.2009.30.3298

266.	Meltzer EO, Lee J, Tripathy I, Lim J, Ellsworth A, Philpot EE. Efficacy and safety of once-daily fluticasone furoate nasal spray in children with seasonal allergic rhinitis treated for 2 wk. *Pediatr Allergy Immunol* 2008;20(3):279-286. doi:10.1111/j.1399-3038.2008.00773.x

267.	Storms WW, Segall N, Mansfield LE, et al. Efficacy and safety of beclomethasone dipropionate nasal aerosol in pediatric patients with seasonal allergic rhinitis. *Ann Allergy Asthma Immunol*. 2013;111(5):408-414. doi:10.1016/j.anai.2013.07.033

268.	Berger WE, Meltzer EO, Amar N, et al. Efficacy of MP-AzeFlu in children with seasonal allergic rhinitis: Importance of paediatric symptom assessment. *Pediatr Allergy Immunol*. 2016;27(2):126-133. doi:10.1111/pai.12540

269.	Prenner BM, Amar NJ, Hampel FC, Caracta CF, Wu W. Efficacy and safety of GSP301 nasal spray in children aged 6 to 11 years with seasonal allergic rhinitis. *Ann Allergy Asthma Immunol*. 2022;129(5):618-626.e2. doi:10.1016/j.anai.2022.07.029
